# From pathogens to partners: temporal and biogeographical patterns in fungal associations of alien trees

**DOI:** 10.1111/nph.71094

**Published:** 2026-03-26

**Authors:** Lukáš Vlk, Iñaki Odriozola, Jan Pergl, Tomáš Větrovský, Jana Kvasničková, Claudia Krüger, Markéta Petružálková, Petr Baldrian, Martin Vojík, Jiří Sádlo, Petr Petřík, Petr Pyšek, Petr Kohout

**Affiliations:** ^1^ Institute of Botany of the Czech Academy of Sciences Zámek 1 252 43 Průhonice Czechia; ^2^ Institute of Microbiology of the Czech Academy of Sciences Vídeňská 1083 142 20 Prague Czechia; ^3^ Faculty of Environmental Sciences Czech University of Life Sciences Prague Kamýcká 129 Praha – Suchdol 165 00 Czech Republic; ^4^ Nature Conservation Agency of the Czech Republic Kaplanova 1931/1 148 00 Prague Czech Republic; ^5^ Faculty of Science Charles University in Prague Albertov 6 128 00 Prague Czechia

**Keywords:** alien trees, mycorrhizal fungi, pathogen spillover, plant–soil feedback, residence time, soil microbiome

## Abstract

- Alien trees reshape belowground fungal communities, but the factors governing the balance between mutualists and pathogens remain unclear. We tested whether residence time, mycorrhizal type, and biogeographical origin shape this balance, and whether alien stands differ from native vegetation.
- We sampled soils beneath 73 alien tree species in 48 chateau parks and native stands. Using ITS2 metabarcoding with guild assignment, we quantified ectomycorrhizal (ECM) and pathogen fungi and analysed predictors with multivariate models and binomial GLMMs, accounting for spatial structure and covariates.
- Symbiotic fungal community composition varied with origin, phylogenetic group and mycorrhizal type. With increasing residence time, ECM alien trees showed higher ECM fungal richness and relative abundance; whereas, contrary to enemy accumulation expectations, pathogen richness and relative abundance declined. Alien arbuscular mycorrhizal (AM) trees harboured more pathogens than ECM trees. Alien tree assemblages had a lower ECM fungal share, twice the pathogen relative richness, and threefold higher pathogen relative abundance than native assemblages.
- Residence time and mycorrhizal type are primary filters shaping belowground trajectories of alien trees, with biogeographical origin patterning community composition. Elevated pathogen loads in alien stands highlight spillover risks to neighbouring vegetation, informing risk assessment and monitoring of alien tree plantings.

## Introduction

Over the past several centuries, human activities have increasingly facilitated the introduction of thousands of alien plant species world‐wide, fundamentally transforming ecosystems and threatening biodiversity on an unprecedented scale (van Kleunen *et al*., 2015; Pyšek *et al*., 2017, 2020b; IPBES, 2023). Among them, alien trees exert disproportionate ecological effects due to their long lifespans and ability to dominate ecosystems, often with far‐reaching consequences (Richardson & Rejmánek, 2011; Simberloff *et al*., 2013; Richardson *et al*., 2014; Castro‐Díez *et al*., 2019). In Europe, various alien tree taxa were historically introduced into managed historical landscapes (e.g. chateau parks) as forestry or ornamental species, and some have established beyond cultivation in surrounding ecosystems (Mack, 2005; Křivánek & Pyšek, 2006; Hulme, 2011, 2015). Understanding how alien trees affect native ecosystems is critical for managing alien tree introductions, assessing invasion risks, and mitigating one of the important impacts of global environmental change. Note that we use the term ‘alien’ for taxa introduced beyond their native range by humans, irrespective of impact or spread (Pyšek *et al*., 2004), and ‘invasive’ for alien taxa that establish self‐sustaining populations and spread (Richardson *et al*., 2000; Blackburn *et al*., 2011) and have documented impacts (IUCN, 2000). Here, we focus on alien trees planted in chateau parks (introduction sites), irrespective of whether or not they currently spread beyond cultivation; thus, we evaluate belowground associations during long‐term residence following introduction rather than invasion success *per se*.

While the aboveground impacts of alien plant species on native ecosystems are well documented, their belowground effects – particularly on soil microbial communities and symbiotic fungi – remain poorly understood (Wardle *et al*., 2011; van der Putten *et al*., 2013; Novoa *et al*., 2021). The effect of alien plants on soil microbes might not only have consequences on microbial‐associated processes, such as carbon sequestration (Hawkins *et al*., 2023) or soil stability (Guo *et al*., 2025), but it can also directly affect the fate of the alien plant species population through microbial biotic interactions. Mycorrhizal fungi, including ectomycorrhizal (ECM) and arbuscular mycorrhizal (AM), form mutualistic associations with roots, being essential for tree nutrient acquisition and overall tree health (Smith & Read, 2008; van der Heijden *et al*., 2015). Conversely, pathogenic fungi may impair tree health and alter the stability of native ecosystems (Winder & Shamoun, 2006). In this study, the term ‘pathogens’ refers to plant‐pathogenic fungi detected in soil; we do not distinguish whether taxa primarily infect above‐ or belowground tissues. Importantly, alien trees can disrupt plant–fungal symbioses by altering the composition, structure, and function of native fungal communities (Gazol *et al*., 2016; Dickie *et al*., 2017). It is critically important to identify the drivers of these disruptions, as they can lead to cascading effects on native vegetation, soil health, and ecosystem resilience, ultimately altering the structure and function of entire ecosystems.

The biogeographical origin and mycorrhizal type of alien trees play a central role in shaping the composition and diversity of symbiotic fungi associated with host trees (Brundrett & Tedersoo, 2018; Tedersoo *et al*., 2020; Eagar *et al*., 2022). Alien trees introduced from distant regions may associate with fungal communities that differ significantly from those in their native range (Reinhart & Callaway, 2006; Dickie *et al*., 2010). At the same time, their mycorrhizal type (ECM vs AM) plays a pivotal role in determining how effectively they form mutualisms in novel environments (Nuñez *et al*., 2009; Moeller *et al*., 2015). A recent study found that ECM fungal communities associated with alien pines (*Pinus*) differed among regions lacking native ECM Pinaceae, reflecting the distinct biogeographic origins of host trees (Vlk *et al*., 2020).

In addition to the above traits, other factors such as propagule pressure, environmental context (e.g. climatic variability and elevation), and most notably residence time were often discussed as correlates of alien tree naturalization (Kowarik, 1995; Lockwood *et al*., 2005; Mitchell *et al*., 2010; Dawson *et al*., 2017; Pyšek *et al*., 2020a); these factors, by affecting host trees even in the period between introduction and escape from cultivation followed by establishment, are expected to shape the composition of associated symbiotic fungal communities. As its residence time increases, an alien tree can accumulate pathogens that are relatively benign to the host itself – likely due to a lack of coevolved host‐specific adaptations – yet damaging to co‐occurring native trees (Parker & Gilbert, 2004; Mitchell *et al*., 2010). Additionally, even at short residence times, alien trees may serve as reservoirs for local generalist pathogens, thereby amplifying spillover or spillback onto native host trees (Reinhart & Callaway, 2006; Flory & Clay, 2013; Purse *et al*., 2013). At the same time, theory and case studies suggest an alternative trajectory: progressive integration with local mutualists – especially incorporation into ECM fungal networks – can enhance mutualist representation and offset pathogen impacts as residence time increases (Nuñez *et al*., 2009; Moeller *et al*., 2015). Together, these contrasting pathways provide competing expectations for how the host tree's residence time interacts with environmental context to structure its belowground symbioses. However, evidence for these dynamics remains poorly resolved across systems, motivating the targeted tests we outline below.

Motivated by these contrasting expectations, we tested three hypotheses using a uniquely structured system – alien trees of diverse origins, mycorrhizal types, and residence times planted in Central European chateau parks – to investigate how host‐associated traits and environment shape symbiotic fungal communities. We hypothesize that (1) fungal communities associated with alien trees differ according to plant biogeographical origin; (2) residence time is a dominant driver of ECM fungal relative richness and abundance (estimated for ECM trees) and of pathogen relative richness and abundance (estimated across all alien trees), to test competing predictions for pathogens derived from enemy accumulation vs mutualist‐integration frameworks; and (3) alien and native trees harbour distinct symbiotic fungal communities, with alien vegetation more strongly associated with pathogens and native trees supporting a greater overall share of ECM fungi. By addressing these hypotheses, our study aims to advance understanding of the ecological processes underpinning plant–fungal symbiotic interactions and provide a basis for risk assessment and targeted monitoring of alien tree plantings, particularly with respect to soil‐detected plant‐pathogenic fungi and potential spillover to neighbouring vegetation.

## Materials and Methods

### Study sites and soil sampling

**Fig. 1:**
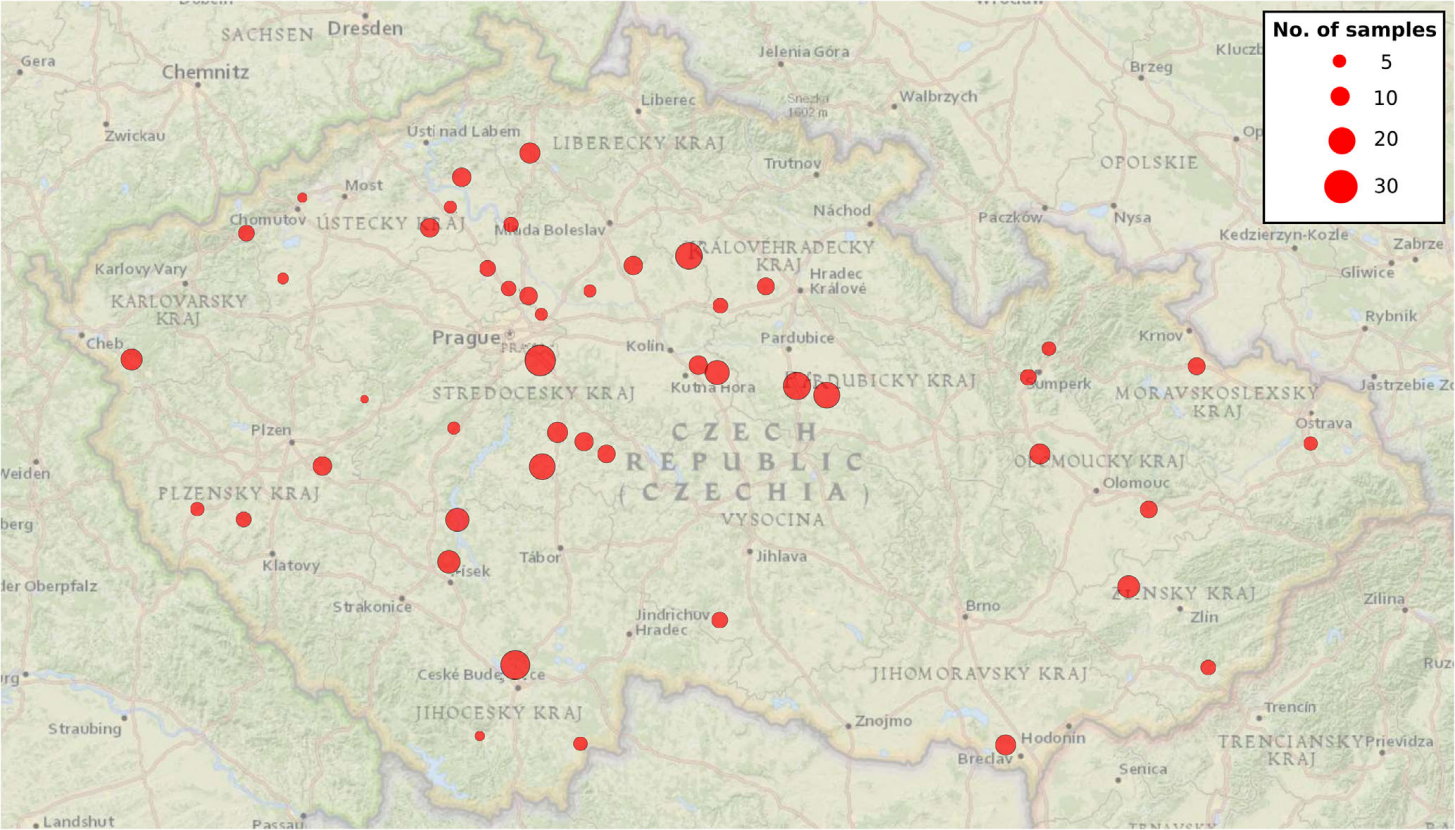
Geographic distribution of the 48 sampled parks in the Czech Republic. Circle size is proportional to the number of sampled alien trees (see legend). The number of individual samples per park is listed in Supporting Information Table S2.

**Fig. 2:**
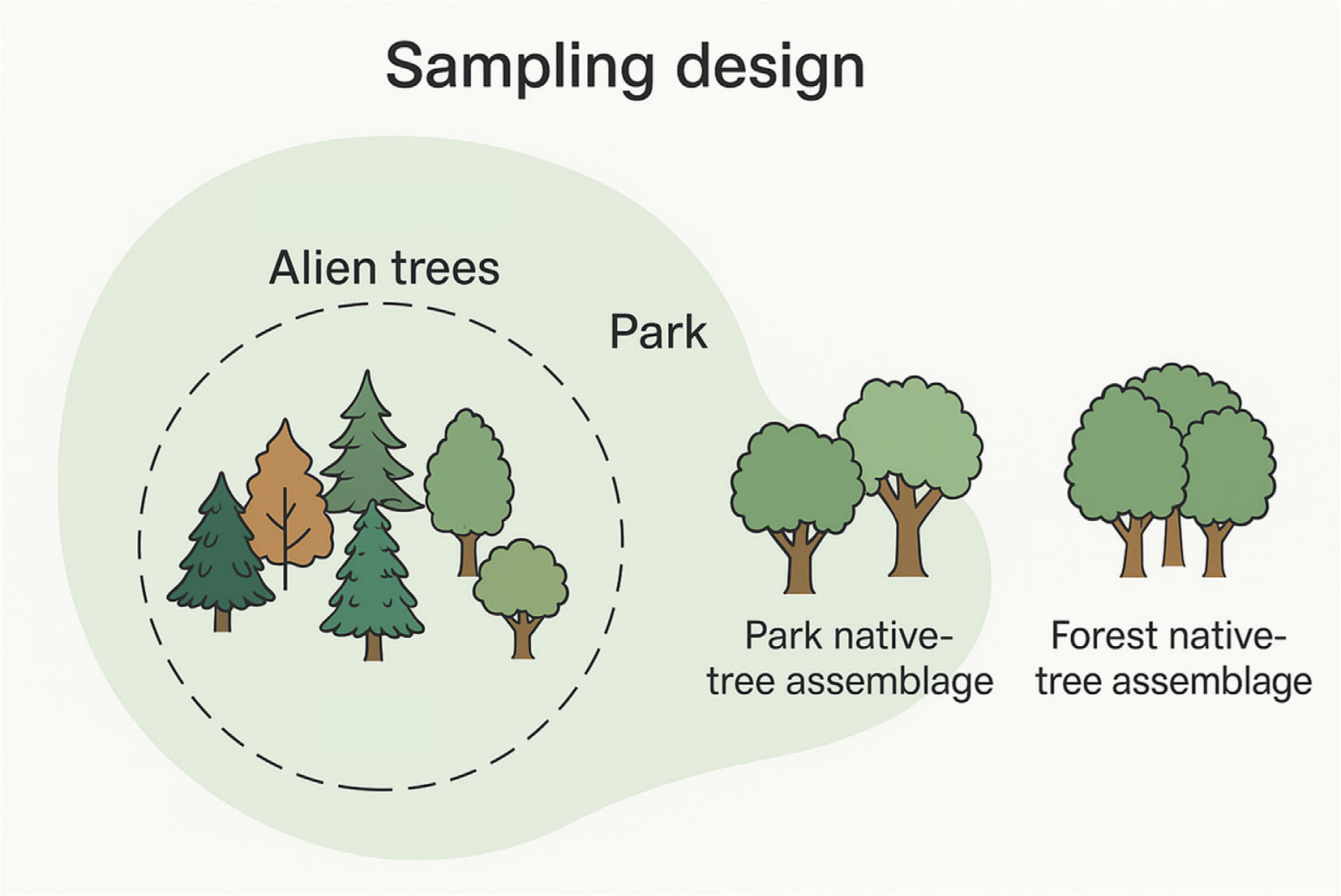
Schematic overview of the sampling design. We sampled alien trees – representing taxa of diverse biogeographical origins, including both gymnosperms and angiosperms – as individuals across 48 chateau parks throughout the Czech Republic. In 38 of these parks, we additionally sampled two native tree assemblages: one located within the same park (‘park‐native tree assemblage’) and one at a nearby forest site (‘forest‐native tree assemblage’). This triad design enabled comparisons between alien and native tree assemblages across spatial and environmental gradients, while broader alien tree sampling ensured robust site‐level coverage.

The study was conducted in 48 historical chateau parks located in the Czech Republic at elevations from 150 to 600 m above sea level (Fig. 1; Supporting Information Table S1; Fig. S1). Parks were selected for their long tradition of cultivating alien tree taxa and for spanning a diverse range of environmental conditions (e.g. elevation, precipitation, and park size). They also encompass a wide range of alien tree taxa deliberately selected to capture variation in biogeographical origin, mycorrhizal type, and residence time. Across all parks, we sampled over 500 individuals representing 73 alien tree species. In each park, we collected soil samples associated with three different types of tree assemblages (Fig. 2). (1) For the first type (further referred to as alien tree assemblage), we identified all alien trees in each park (Vojík *et al*., 2020). Then, we sampled fully grown and healthy individuals to cover the set of alien tree species present in the park, their assignment to host groups (AM/ECM; angiosperm/gymnosperm) and biogeographical origins. Most species were represented by one individual per park in sampling (or two to three where the tree species was frequent or to balance host/origin strata). We provide totals by species and group in Table 1, and per park sample sizes in Table S2. Under each tree, we collected four 4‐cm diameter soil cores at a depth of 10 cm placed evenly at least 1 m from the tree trunk and pooled them, resulting in 520 samples of soil associated with alien trees (four subsamples per tree), in total.

**Table 1:** Replication summary of alien trees by biogeographical origin, plant group and mycorrhizal type.

Biogeographical origin	Plant group	Mycorrhizal type	Species	Individuals	Parks
Asia	Angiosperm	AM	12	46	22
Asia	Gymnosperm	AM	5	33	22
Asia	Gymnosperm	ECM	6	8	8
Europe	Angiosperm	AM	2	6	4
Europe	Angiosperm	ECM	1	20	14
Europe	Gymnosperm	ECM	4	6	6
Hybrid	Angiosperm	AM	3	38	27
North America	Angiosperm	AM	15	112	39
North America	Angiosperm	ECM	7	16	12
North America	Gymnosperm	AM	8	62	27
North America	Gymnosperm	ECM	10	173	43

*Species*, unique alien tree species sampled in each category across all parks; *Individuals*, number of tree‐level soil samples (alien trees only; *n* = 520); *Parks*, number of parks where those species were sampled.

Then, in a subset of the studied parks (38 out of 48), we established two sampling plots in (2) native tree assemblages within parks (‘park‐native’) and (3) nearby forests outside parks (‘forest‐native’) as native reference assemblages for comparison with park‐alien‐tree assemblages (Fig. 2). These circular plots, *c*. 50 m in diameter (i.e. *c*. 1963 m^2^), were located to contain at least 20 individual trees (all of them native to Central Europe). The plots established outside the parks were in relatively close proximity to the park plots (2–15 km from the park boundary, depending on the location of the nearest seminatural or natural tree patch). Representative photographs of the sampling locations and tree assemblage types are provided in Fig. S2. One composite soil sample, pooled from 20 randomly distributed 4‐cm soil cores, was collected in each of the two plots (park and forest), resulting in a total of 76 soil samples from the native park and forest vegetation (Table S3). We stored the soil for a maximum of 2 d at a temperature of 4°C. Large roots, litter, and stones were removed from the samples that were stored at −20°C before further analyses. The soil was then sieved through a 2‐mm diameter mesh and mixed. We recorded the GPS coordinates of all samples. We recorded the tree species present in each plot and estimated their canopy covers (in %). Data on the geographic origin of alien tree species were obtained from the Plants of the World Online database (POWO, 2020). To determine the mycorrhizal type of alien trees, we used the FungalRoot database of plant mycorrhizal associations (Soudzilovskaia *et al*., 2020). Although this database – like most large compilations drawing on many sources – has some coverage and consistency limitations (Bueno *et al*., 2021), the majority of the sampled tree species are well‐studied taxa for which mycorrhizal status is relatively well known. FungalRoot also includes a mixed/dual ECM–AM category; however, none of the host taxa sampled in our study was classified as such, and they were therefore treated as AM or ECM.

### Molecular analysis: DNA extraction and sequencing of PCR amplicons

DNA was extracted in triplicate for each sample (3 × 250 mg of frozen soil) using the DNeasy PowerSoil Pro Kit (Qiagen) according to the manufacturer's standard protocol and eluted in 50 μl of elution buffer. Respective replicates were further pooled before subsequent PCRs. Extracted DNA was inspected using a NanoDrop ND‐1000 Spectrophotometer (NanoDrop, Wilmington, DE, USA). We amplified the ITS2 region of the rDNA from soil samples using the primers gITS7 and ITS4 (Ihrmark *et al*., 2012). Both primers were tagged with the same sample‐specific molecular identifiers to decrease potential mistakes caused by tag switching. Each PCR reaction was performed in a total volume of 30 μl and contained 0.7 U Taq DNA Polymerase (Fermentas, St. Leon‐Rot, Germany), 10× PCR Blue Buffer (without MgCl_2_) (Top‐Bio, Vestec, Czech Republic), 0.25 mM each dNTP, 2.5 mM MgCl_2_, 20 μg bovine serum albumin (Thermo Fisher Scientific, Waltham, MA, USA), 0.2 μM each primer, and 1 μl of the DNA extract. We used the following thermal cycling conditions: initial denaturation for 5 min at 94°C; 35 cycles of 94°C for 30 s, 45°C for 30 s, and 72°C for 45 s, followed by an extension of 20 min at 72°C. Each DNA extract was amplified in triplicate. PCR products were visualized on a 1% agarose gel. The pooled triplicates were purified through columns with the QIAquick PCR Purification Kit (Qiagen) according to the manufacturer's protocol and eluted into 20 μl of ddH_2_O. DNA concentrations of the amplicon pools were quantified using a Qubit 2.0 Fluorometer (Thermo Fisher Scientific) with High Sensitivity Assay Kit. Duplicates of negative PCR controls (with ddH_2_O instead of a template) were processed in the same way as the experimental samples and included in each sequencing library. Purified samples were pooled into sequencing libraries in equimolar ratios. Libraries were produced using the TruSeq PCR‐free Kit (Illumina, San Diego, CA, USA) and sequenced on an Illumina MiSeq (2 × 250 bp at the Laboratory of Environmental Microbiology, Institute of Microbiology of the Czech Academy of Sciences).

### Bioinformatics

Paired‐end Illumina reads (*c*. 10 million in total) were joined to generate single reads using fastq‐join (Aronesty, 2011) within the Seed 2 v.2.1.05 pipeline (Větrovský *et al*., 2018). The joined sequences were then demultiplexed and trimmed; all sequences with a mean quality Phred score < 30 and/or barcode mismatches were discarded. ITSx v.1.0.8 (Bengtsson‐Palme *et al*., 2013) was used to extract the ITS2 region and detect chimeric sequences. To recognize fungal taxa, we used ‘Species Hypothesis’ (SH; Kõljalg *et al*., 2013) as a molecular analogue of the classical biological species concept (Kõljalg *et al*., 2005). Compared to the *de novo* creation of OTU or ASV, SH are predefined molecular taxonomic units curated by fungal taxonomists and compiled within the UNITE database (Abarenkov *et al*., 2024a). Therefore, representative sequences of SH represent a suitable reference dataset for the identification of fungal taxa from sequencing data. Using BLASTn (Altschul *et al*., 1990), ITS2 reads were assigned directly (binned) to the best matching SH (general ‘dynamic’ release 04.04.2024), requiring ≥ 98.5% sequence similarity and ≥ 95% alignment coverage (Abarenkov *et al*., 2024b). In cases where a sequence matched more than one SH, following the above‐mentioned criteria, a priority was given to the SH with a higher similarity score. No *de novo* OTU clustering or ASV inference was performed; per‐sample read counts were summarized at the SH level for all downstream analyses. The same methodology of sequence assignment to fungal SH is used in the GlobalFungi database (for more details, see Větrovský *et al*., 2020). Reads failing the thresholds remained unassigned and were excluded from the SH‐level community matrices. Each fungal SH was assigned to a trophic guild at the genus level using FungalTraits (Põlme *et al*., 2020). Accordingly, ‘pathogens’ refers to taxa assigned as plant pathogens in FungalTraits and detected in belowground samples. Taxa for which assignment was not well supported at the genus level were left unassigned. We used UNITE Species Hypotheses (SH) as the operational taxonomic unit for all downstream analyses because SHs provide curated, reproducible species‐level groupings across datasets (Kõljalg *et al*., 2013). Using SHs also avoids artificial inflation of fungal richness caused by intragenomic ITS variability that can inflate *de novo* OTU/ASV counts, and therefore makes comparisons across many host tree species and sites more robust (Abarenkov *et al*., 2024a).

### Statistical analyses

All statistical analyses were conducted in R 4.4.0 (R Core Team, 2024), with full scripts and diagnostic plots provided in the Supporting Information. ITS2 sequence reads were assigned to UNITE SHs following the similarity thresholds described above. Read counts for each sample were Hellinger‐transformed to reduce the influence of dominant taxa while retaining relative species representation, and Bray–Curtis dissimilarities computed from the Hellinger‐transformed data were used in all multivariate analyses. Because bulk‐soil ITS2 metabarcoding, using general fungal or eukaryotic primers, under‐represents Glomeromycota (Stockinger *et al*., 2010), we pre‐specified a robustness analysis in which AM fungal taxa were excluded from proportional denominators; model formulas and outputs for this sensitivity check are provided in Table S4. In this study, we refer to ‘community structure’ as the proportional representation of fungal trophic guilds, quantified by the relative richness and abundance of ECM and pathogenic fungi within each soil sample. To account for phylogenetic relationships among alien tree species, we constructed a tree using PhyloT (Letunic, 2015). The first axis of the resulting phylogenetic PCNM explained 92% of the variation and primarily distinguished angiosperms from gymnosperms. It was also the only axis retained during the forward selection procedure across all response variables analysed in this study. Accordingly, we defined the categorical variable plant group (angiosperm vs gymnosperm) for use in subsequent analyses. Residence time was taken from DAWIS – Database of Alien Woody Species in the Czech Republic (Křivánek & Pyšek, 2008) as the number of years since the first verified record of introduction of an alien tree species to the Czech Republic. This metric provided a better model fit than the year of introduction to Europe, so we used it in all main analyses.

### Multivariate analyses

Differences in overall symbiotic fungal community composition among individual alien trees were examined with PERMANOVA (adonis2, vegan; Oksanen *et al*., 2012) on Bray–Curtis dissimilarities of Hellinger‐transformed SH tables, using 999 permutations blocked by park identity. Explanatory variables included host‐associated factors (plant group: angiosperm vs gymnosperm; mycorrhizal type: ECM vs AM; continent of origin; residence time) and environmental/stand covariates (mean annual temperature, MAT; mean annual precipitation, MAP; park area; ECM tree proportion; angiosperm‐tree proportion; alien tree richness). Because biogeographical origin, mycorrhizal type, and plant group were not independent in our data, we assessed potential confounding. We additionally performed a sensitivity analysis excluding hybrid‐origin trees to verify that partial dependence among host traits did not drive key inferences (Table S5). We evaluated interaction terms among these host traits and retained the origin × mycorrhizal type interaction because it was significant and improved model fit; all sums‐of‐squares are marginal (order‐independent) with permutations blocked by park. Before model finalization, we assessed collinearity among covariates using variance inflation factors (VIFs; factors treatment‐coded); elevation showed high collinearity with MAT and was excluded, while MAT and MAP were retained (ecological inferences unchanged). We evaluated both a categorical factor (continent of origin) and a continuous measure (distance to the native range centroid) as alternative descriptors of biogeographical origin and retained the categorical form because it provided the better fit and consistent results across analyses. Significant PCNM eigenvectors were selected by forward selection (forward.sel, adespatial) with 999 permutations at *α* = 0.05 (Blanchet *et al*., 2008) and included as spatial covariates (Borcard & Legendre, 2002). In addition to the global interaction model, we ran a complementary set of single‐predictor models in which each ecological variable was tested individually while the PCNMs remained in the model, to report stand‐alone marginal *R* ^2^ and *F*‐values. Full marginal PERMANOVA results are provided in Table S6. NMDS ordinations were initially explored (ecodist; Goslee & Urban, 2015), but stress exceeded 0.2; we therefore used principal coordinates analysis (PCoA) for visualization. Pairwise PERMANOVAs comparing levels of origin, mycorrhizal type, and plant group followed the same blocked design, retained the PCNMs as covariates, and likewise used marginal sums‐of‐squares to test the focal factor while controlling for spatial structure.

PERMANOVA was also applied to assess differences in fungal community structure among three tree assemblages: (1) the park‐alien‐tree assemblage (all alien cores pooled within a park), (2) the park‐native tree assemblage, and (3) the forest‐native tree assemblage. Analyses used marginal (order‐independent) sums‐of‐squares with 999 permutations blocked by park identity. Models included the environmental covariates MAT, MAP, and elevation, the number of pooled soil cores per sample, and the single forward‐selected spatial PCNM axis (PCNM2). Where a given comparison involved samples from the same park, permutations were constrained to within‐park strata. For each pairwise test (park‐alien vs park‐native, park‐alien vs forest‐native, park‐native vs forest‐native), we retained the 38 parks in which both native assemblage types were present to ensure a balanced design and ran 9999 blocked permutations; resulting *p*‐values were Bonferroni‐adjusted. Although multivariate dispersion differed among groups, our design was balanced (equal group sizes), and previous work has shown that PERMANOVA remains robust to such dispersion heterogeneity under balanced designs (Anderson & Walsh, 2013).

### Univariate analyses

To examine differences in the relative richness and abundance of ECM and pathogenic fungal guilds at the individual tree level, we used binomial generalized linear mixed‐effects models (GLMMs; glmer function, lme4 package; Bates *et al*., 2015). For ECM fungal relative richness and abundance, the analyses were restricted to ECM‐host trees; pathogen relative richness and abundance were modelled across all alien trees. The models included the same set of fixed effects as the multivariate analysis of alien tree symbiotic fungal community composition (see previously), along with a park‐level random intercept, except that mycorrhizal type was included only in the pathogen models. At the tree assemblage level, we modelled the relative richness and abundance of ECM and pathogenic fungi with binomial generalized linear mixed‐effects models. The response variable consisted of the proportion of ECM or pathogenic species (or sequences) in a sample out of the total number of guild‐assigned species (or sequences in the same sample). Because amplicon sequencing yields compositional read counts, we use ‘relative abundance’ to denote the proportion of sequences assigned to the focal guild within each soil sample; we did not estimate absolute fungal abundance or biomass (e.g. by qPCR or PLFA). Tree assemblage group (park‐alien, park‐native, forest‐native) was the focal fixed effect, while environmental covariates (MAT, MAP, elevation, proportion of ECM trees) were included to control for potential confounding effects. A random intercept for the park accounted for site‐level clustering, and a random observation‐level effect accounted for residual overdispersion. Predictor significance was assessed with likelihood‐ratio *χ* ^2^ tests, and Tukey‐adjusted marginal means were obtained with the emmeans package (Lenth, 2017). We assessed spatial autocorrelation in all model residuals by calculating Moran's *I* (Cliff & Ord, 1981), using the spdep package in R (Bivand *et al*., 2011), to evaluate whether model residuals exhibited non‐random spatial structure. To assess robustness to potential AM fungal under‐detection by ITS2, we recomputed pathogen proportional responses after excluding AM fungal taxa from the denominators and re‐fit the four GLMMs; the direction and significance of focal effects were unchanged (Table S4).

## Results

The chateau parks included in this study varied considerably in the number of cultivated alien tree species. Such differences subsequently affected the number of samples obtained from alien trees in each park, as we tended to collect more samples in parks containing more alien tree species (mean = 10.8 samples per park, median = 9.5; Table 1). Most samples were collected in the Průhonice chateau (29) and the fewest in Zbiroh (2). We sampled a total of 73 alien tree taxa (for simplicity further referred to as ‘species’), 39 of which were present in at least three chateau parks. The most frequently sampled alien trees were *Pseudotsuga menziesii* (Mirb.) Franco (44 samples from 28 parks), *Picea pungens* Engelm. (38 samples, 23 parks), and *Pinus strobus* L. (35 samples, 22 parks). The majority of alien trees originated in North America (69.8% of individual trees), followed by Asia (16.7%) and Europe (6.2%). Hybrids, with unattributable geographic origin, comprised 7.3% of recorded tree taxa. In terms of plant families, most alien trees belonged to Pinaceae (36.0%), Cupressaceae (14.4%), Fabaceae (12.3%), and Fagaceae (6.2%); there were 45.8% of angiosperms and 54.2% gymnosperms. In terms of mycorrhizal type, the majority of alien trees were AM (57.1%), and the rest were ECM (42.9%).

The resulting dataset consisted of 520 fungal communities associated with alien trees (Table S2) and 76 fungal communities associated with native tree assemblages inside and outside the subset of 38 parks (Table S3). A total of 4972 572 fungal sequences associated with alien or native trees in Czech chateau parks were recovered from 10 059 381 raw sequences. Sample‐based rarefaction curves are provided in Fig. S3. Across all samples, 13 067 distinct UNITE SHs were recovered, including 3045 assigned to symbiotic guilds (ECM, AM, plant pathogens, endophytes, animal parasites); the remainder were saprotrophs, epiphytes, or unassigned. Most of the detected fungal SHs belonged to the phylum Ascomycota (49.8%), followed by Basidiomycota (30.6%) and Glomeromycota (2.4%), the remaining 17.2% belonged to various or unassigned phyla. Saprotrophic fungal SHs comprised the largest proportion of fungal trophic guilds (32.9%), followed by pathogens (7.4%), ECM fungi (8.4%), AM fungi (1.3%), and endophytes (1.0%). Species with unclear classification in the trophic guild made up 43.5% of the total. A substantial share of unassigned SHs fell within *Fungi incertae sedis* or broad Ascomycota or Basidiomycota categories; early‐diverging phyla contributed smaller proportions (Table S7). The remaining unassigned reads primarily consisted of non‐target DNA (e.g. plant or protist) or low‐quality reads. The most abundant ectomycorrhizal and putative plant‐pathogenic genera, together with their ecological classification, are listed in Fig. S4.

### Biogeographical origin patterns symbiotic fungal community composition

**Fig. 3:**
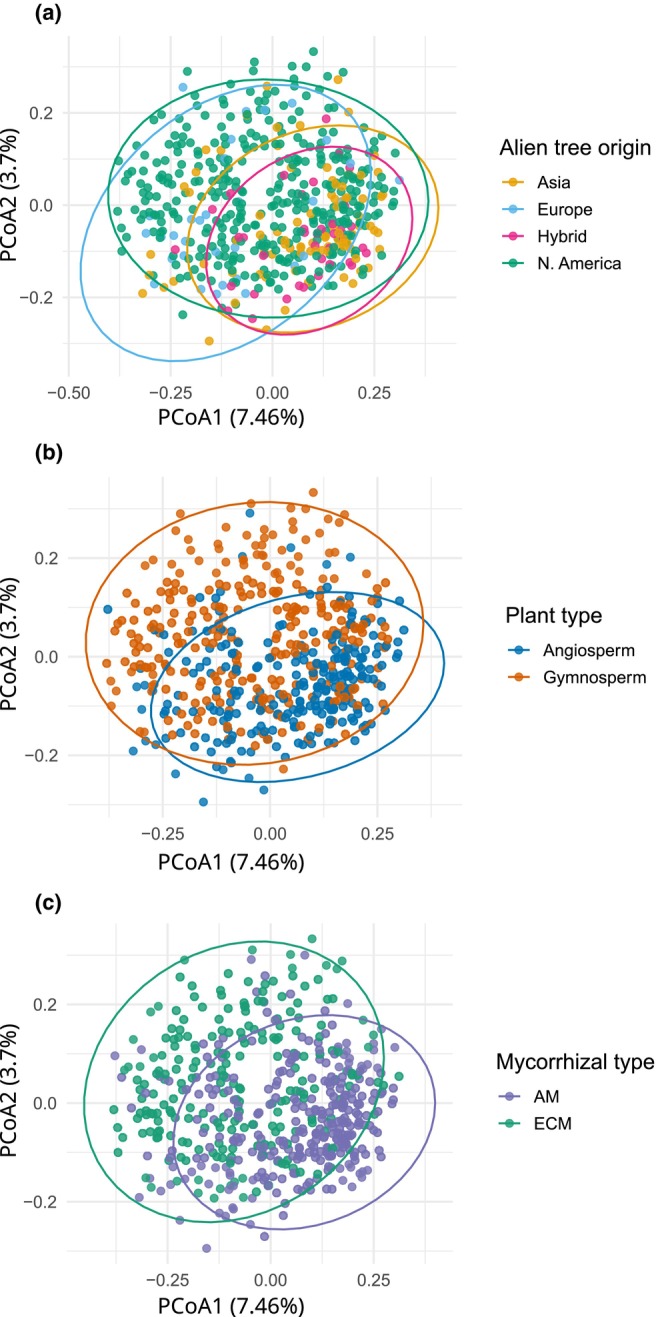
Fungal community composition is structured by biogeographical origin, plant group, and mycorrhizal association of alien trees. Principal coordinates analysis (PCoA) of Bray–Curtis dissimilarities calculated on Hellinger‐transformed fungal communities sampled beneath alien trees in 48 parks. Each point is one sample. Panels show colouring by (a) host biogeographical origin (Asia, Europe, North America, Hybrid), (b) plant group (angiosperm vs gymnosperm), and (c) mycorrhizal type (AM vs ECM). Ellipses denote 95% confidence intervals. Axes show the percentage of variance explained (PCoA1 = 7.46%, PCoA2 = 3.70%). PERMANOVA with park‐blocked permutations (marginal SS) indicated significant effects of mycorrhizal type (df = 1, pseudo‐*F* = 3.93, *R* ^2^ = 0.0067, *P* = 0.001) and plant group (df = 1, pseudo‐*F* = 3.10, *R* ^2^ = 0.0053, *P* = 0.001), and a small but significant origin × mycorrhizal type interaction (df = 2, pseudo‐*F* = 1.43, *R* ^2^ = 0.0048, *P* = 0.004).

The composition of symbiotic fungal communities associated with alien trees differed significantly among host biogeographical origins (Fig. 3a, Table S6). In a global PERMANOVA with park‐blocked permutations and forward‐selected PCNMs as covariates, we included an origin × mycorrhizal type interaction; the interaction was significant (df = 2, pseudo‐*F* = 1.43, *R* ^2^ = 0.0048, *P* = 0.004). Complementary single‐predictor marginal tests identified biogeographical origin (df = 3, pseudo‐*F* = 2.50, *R* ^2^ = 0.0132, *P* = 0.001), mycorrhizal type (df = 1, pseudo‐*F* = 3.93, *R* ^2^ = 0.0067, *P* = 0.001), plant group (df = 1, pseudo‐*F* = 3.10, *R* ^2^ = 0.0053, *P* = 0.001), residence time (df = 1, pseudo‐*F* = 1.86, *R* ^2^ = 0.0032, *P* = 0.002) and MAT (df = 1, pseudo‐*F* = 2.64, *R* ^2^ = 0.0045, *P* = 0.011) as significant predictors (Table S6). MAP, park area, ECM tree proportion, angiosperm proportion and alien tree richness were not significant. Pairwise PERMANOVAs confirmed significant differences in fungal community composition among alien trees of different origins (all *P* < 0.005; Fig. 3a): Asian vs European (*F* = 2.94, *P* = 0.001), North American vs Asian (*F* = 2.92, *P* = 0.001), North American vs European (*F* = 2.25, *P* = 0.001), Hybrid vs North American (*F* = 2.22, *P* = 0.001), Hybrid vs European (*F* = 2.08, *P* = 0.002) and Asian vs Hybrid (*F* = 1.41, *P* = 0.004). Because plant group (angiosperm vs gymnosperm), mycorrhizal type, and biogeographical origin covary in our dataset, these effects should not be interpreted as fully independent causal drivers. Group dispersions (distance to group centroid) differed for origin, mycorrhizal type, and plant group (betadisper with park‐blocked permutations, all *P* = 0.001). Mean ± SE distances: origin – Asia 0.550 ± 0.008, Europe 0.593 ± 0.008, Hybrid 0.535 ± 0.011, N. America 0.586 ± 0.003; mycorrhizal type – AM 0.556 ± 0.004, ECM 0.598 ± 0.004; plant group – Angiosperm 0.563 ± 0.005, Gymnosperm 0.588 ± 0.004. Because origin groups were unequal in size, dispersion differences could influence PERMANOVA; we therefore report betadisper alongside blocked PERMANOVA and interpret location effects with this caveat.

### Residence time, mycorrhizal type, and biogeographical origin influence the richness and abundance of fungal communities

**Fig. 4:**
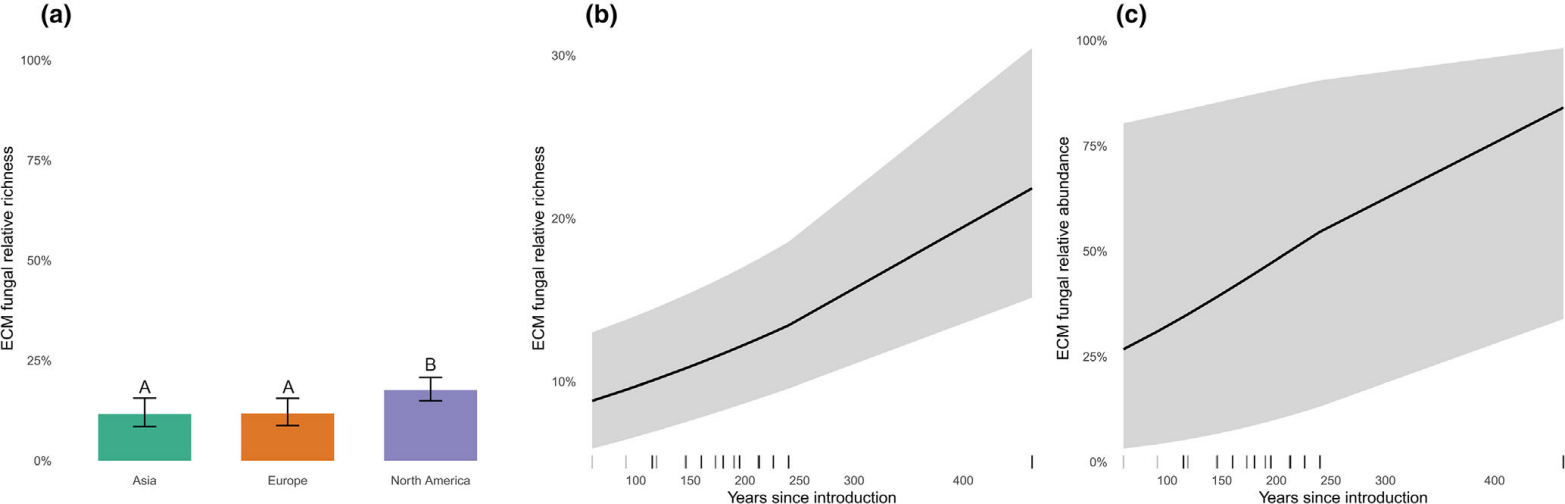
Residence time shapes ectomycorrhizal (ECM) fungal associations with alien trees, represented by their relative richness and abundance. Results from binomial generalized linear mixed models (GLMMs) show that ECM fungal relative richness differs among host biogeographical origins (a) and increases with residence time within ECM hosts for both relative richness (b) and relative abundance (c). Shaded ribbons indicate 95% confidence intervals. GLMMs included park identity as a random effect and accounted for environmental covariates; ECM models were fit on ECM hosts only.

**Fig. 5:**
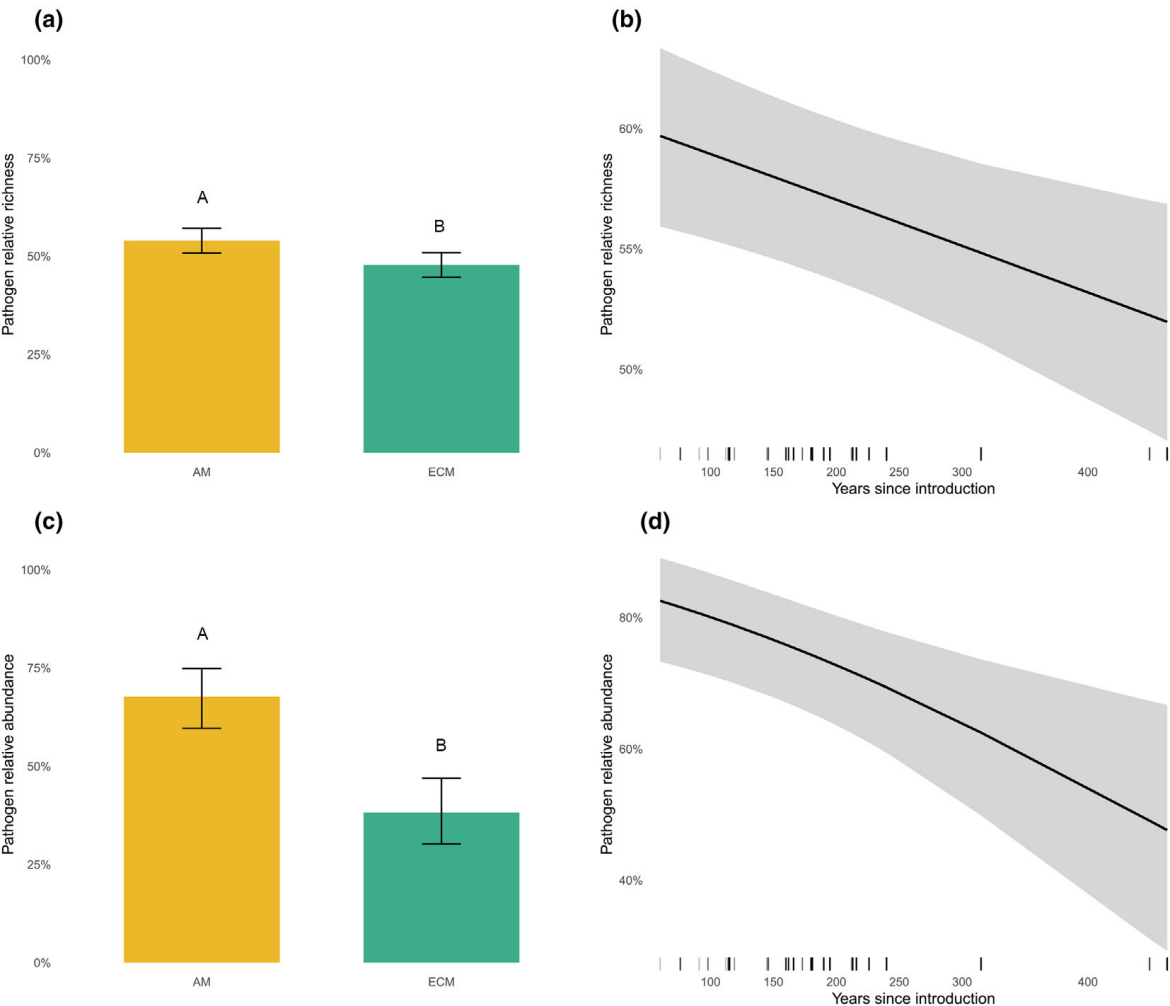
Residence time and mycorrhizal type structure pathogen associations with alien trees. Binomial GLMMs indicate that pathogen relative richness (a) and relative abundance (c) are higher in AM than ECM hosts, while both pathogen relative richness (b) and relative abundance (d) decline with residence time across alien trees. Shaded ribbons indicate 95% confidence intervals. GLMMs included park identity as a random effect and accounted for environmental covariates; pathogen models were fit across all alien trees.

Mycorrhizal type and residence time emerged as consistent predictors of symbiotic fungal community structure, with distinct effects on ECM fungi and pathogens; biogeographical origin showed fungal group–dependent effects and was most evident for ECM fungi (Table 2; Figs 4, 5). In ECM trees, longer residence times were associated with increases in ECM fungal relative richness (χ^2^ = 22.96, *P* < 0.001) and relative abundance (χ^2^ = 4.94, *P* = 0.026). Regarding pathogens, residence time was a significant predictor of both responses, with shorter residence times associated with higher pathogen relative richness (χ^2^ = 8.14, *P* = 0.004) and relative abundance (χ^2^ = 10.12, *P* = 0.001). These inferences for pathogens were qualitatively unchanged when AM taxa were excluded from denominators (Table S4).

**Table 2:** Summary of binomial GLMMs testing effects of ecological predictors on the relative richness and abundance of ectomycorrhizal (ECM) fungi and of pathogens associated with alien trees.

Response	Significant predictors	Effect direction	χ^2^	*P*‐value
ECM fungal relative richness	Residence time	↑	22.96	< 0.001***
	Origin	–	19	< 0.001***
	MAP	↑	6.68	< 0.001***
	Proportion of angiosperms	↓	3.89	0.048*
ECM fungal relative abundance	Residence time	↑	4.94	0.026*
Pathogen relative richness	Mycorrhizal type	–	43.73	< 0.001***
	MAT	↑	27.65	< 0.001***
	Origin	–	14.85	0.002**
	Residence time	↓	8.14	0.004**
Pathogen relative abundance	Mycorrhizal type	–	43.25	< 0.001***
	Residence time	↓	10.12	0.001**
	MAT	↑	4.47	0.035*

ECM fungal responses were modelled only for ECM‐host trees, whereas pathogen responses were modelled across all alien trees. Results are likelihood‐ratio tests from binomial GLMMs (*n* = 520 soil samples). Only significant predictors are shown. All models included the full set of covariates: mean annual temperature (MAT), precipitation (MAP), park area, alien tree richness, ECM tree proportion, and angiosperm proportion. Symbols ↑/↓ indicate the direction of effect on fungal proportional representation. Asterisks denote significance levels: *P < 0.05; **P < 0.01; ***P < 0.001.

Beyond residence time, ECM fungal relative richness varied with biogeographical origin (North American trees were associated with significantly higher ECM fungal relative richness compared to European and Asian trees), increased with precipitation (MAP), and declined with the local proportion of angiosperms (Table 2). For pathogens, AM hosts harboured higher relative richness and abundance than ECM hosts, and warmer sites (higher MAT) supported more pathogens. The apparent biogeographical origin effect for pathogens was driven by higher pathogen relative richness in Asian and North American alien trees compared with hybrids (Tukey‐adjusted: Asia > Hybrid, *P* = 0.001; North America > Hybrid, *P* = 0.027; Europe did not differ significantly from other origins). However, this origin pattern primarily reflects differences involving the hybrid‐origin category; when hybrid‐origin trees were excluded, differences among continental origins were weaker and the evidence for differences among continental origins decreased (Table S5).

### Alien vegetation hosts distinct symbiotic fungal communities compared to native vegetation

The composition of symbiotic fungal communities differed significantly among the park‐alien tree, park‐native tree, and forest‐native tree assemblages (PERMANOVA: df = 2106, *F* = 6.04, *R* ^2^ = 0.088, *P* = 0.001), after accounting for park identity and environmental covariates (Table S8). The full model explained 16.8% of the variance (adjusted *R* ^2^ = 0.168). Bonferroni‐adjusted pairwise PERMANOVAs showed that each tree assemblage type harboured a distinct community: park‐alien tree assemblages differed from park‐native tree assemblages (*F* = 12.00, *P* < 0.001) and from forest‐native tree assemblages (*F* = 15.82, *P* < 0.001), and park‐native tree and forest‐native tree assemblages also differed from one another (*F* = 2.11, *P* < 0.001).

**Fig. 6:**
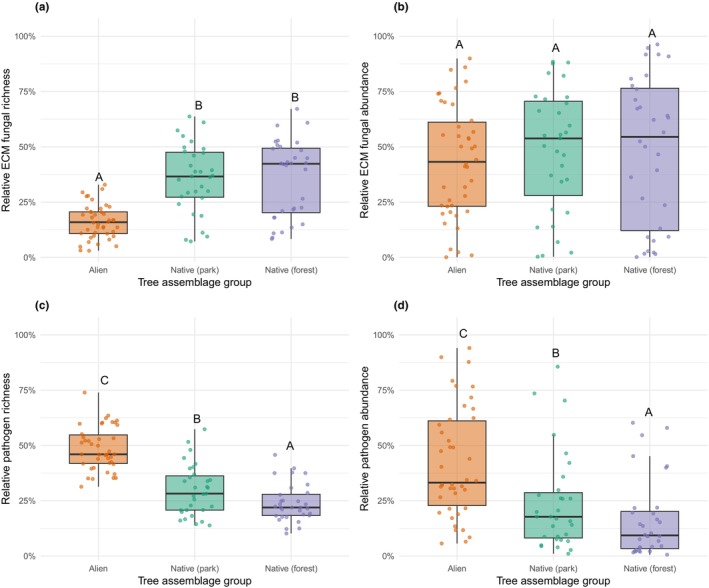
Opposing patterns of ECM fungal and pathogen representation across tree assemblages. Relative ECM fungal richness (a), relative ECM fungal abundance (b), relative pathogen richness (c), and relative pathogen abundance (d) across ECM park‐alien, park‐native, and forest‐native tree assemblages. Letters indicate Tukey‐adjusted pairwise comparisons from binomial GLMMs; groups not sharing a letter differ significantly (*P* < 0.05). Park‐alien trees hosted a lower proportion of ECM fungi and a markedly higher proportion of pathogens compared to native assemblages, particularly in forests.

ECM and pathogen fungi displayed opposite trends across tree assemblage groups (Fig. 6, Table S9). Alien ECM tree assemblages contained a significantly lower share of ECM fungal SHs than both park‐native and forest‐native tree assemblages (χ^2^ = 33, *P* < 0.001; Fig. 6a), but the relative abundance of ECM fungi did not differ among the three groups (χ^2^ = 0.3, *P* = 0.86, Fig. 6b). Both pathogen relative richness and abundance were markedly elevated in park‐alien tree vegetation (richness χ^2^ = 72.54, abundance χ^2^ = 28.53; both *P* < 0.001, Fig. 6c,d). Pairwise contrasts showed that park‐alien assemblages harboured roughly twice the pathogen fungal richness (odds ratio [OR] = 2.5) and threefold the pathogen abundance (OR = 3.3) of native tree assemblages. Park‐native tree assemblages were intermediate, differing significantly from forest assemblages for pathogen richness and abundance (richness OR = 1.38, *P* = 0.013; abundance OR = 2.22, *P* = 0.011) but not for ECM richness and abundance. The effects of environmental covariates (MAT, elevation) were generally non‐significant, whereas MAP had a weak positive effect on ECM fungal richness (Table S9). The park‐level random intercept accounted for modest but significant clustering (intraclass correlation coefficient, ICC ≈ 0.02), while the inclusion of an observation‐level random effect eliminated residual overdispersion (Pearson χ^2^/df ≈ 1), confirming adequate model fit.

## Discussion

Our study demonstrates that biogeographical origin, residence time, and plant mycorrhizal type are key determinants of belowground symbiotic fungal communities associated with long‐cultivated alien trees in Czech chateau parks. Leveraging an extensive dataset from 48 chateau parks and over 500 soil samples collected from 73 alien tree species, we employed high‐throughput sequencing of eDNA extracted from soil and ecological modelling to disentangle how host‐associated and environmental factors jointly shape the soil symbiotic fungal community composition and structure.

Consistent with our first hypothesis, biogeographical origin was identified as a significant predictor of symbiotic fungal community composition, even after accounting for other ecological and environmental factors (Fig. 3, Table S6). Alien trees from Asia, Europe, and North America, as well as hybrids, support different fungal communities, potentially reflecting their evolutionary interactions with native fungi and biogeographically structured variation in their traits (Brundrett & Tedersoo, 2018; Lutzoni *et al*., 2018). This finding supports the idea that the biogeographical origin of alien trees is a strong predictor of their symbiotic fungal community composition following introduction, underscoring the enduring influence of evolutionary history on belowground interactions in new areas (Reinhart & Callaway, 2006; Pringle *et al*., 2009; Dickie *et al*., 2017; Vlk *et al*., 2020). The aboveground impacts of alien plants are well documented (Vilà *et al*., 2011; Pyšek *et al*., 2012; Simberloff *et al*., 2013; Richardson *et al*., 2014; IPBES, 2023; Bacher *et al*., 2025), but our study provides regional‐scale empirical evidence that alien trees also restructure belowground fungal networks, altering the relative richness, abundance, and composition of both mutualistic and pathogenic guilds (see also Novoa *et al*., 2020). Given the covariance among origin, mycorrhizal type and plant group, we interpret these patterns as correlational rather than independent causal effects.

Importantly, residence time and mycorrhizal type jointly shaped soil symbiotic fungal communities, with contrasting patterns for ECM fungi and pathogens (Figs 4, 5). In ECM alien trees, longer residence times were associated with higher ECM fungal richness and abundance – that is, a greater proportional representation of ECM fungi within ECM hosts – which could enhance nutrient uptake and disease tolerance and ultimately facilitate naturalization (Nuñez *et al*., 2009; Dickie *et al*., 2017). Two non‐exclusive mechanisms may underlie this pattern. First, progressive integration into local ECM networks can increase partner discovery and compatibility through time, expanding the pool of effective symbionts and reinforcing benefits such as nutrient foraging and pathogen suppression (Nuñez *et al*., 2009; van der Heijden *et al*., 2015; Dickie *et al*., 2017). Second, demographic and management filtering may have preferentially retained plantings that established strong ECM associations early on, while individuals with poor symbiont support failed or were removed – yielding today's older cohorts enriched in ECM partners (consistent with long‐term horticultural selection in parks). More broadly, evidence from invasion‐focused studies suggests that many alien plants with generalist mutualisms can recruit partners in new regions over time – most clearly for pollination (Pyšek *et al*., 2011), where alien plants (often invasive) integrate into native networks via common generalist pollinators – whereas specialized systems integrate less readily (Daehler, 1998; Vilà *et al*., 2009; Traveset & Richardson, 2014). For nitrogen‐fixing symbioses, outcomes are mixed: some legumes partner successfully with locally available or co‐introduced rhizobia, but compatibility and symbiotic effectiveness vary among regions and lineages (Rodríguez‐Echeverría *et al*., 2011; Horn *et al*., 2014; Simonsen *et al*., 2017). Our ECM result, therefore, highlights a general mechanism – mutualist build‐up through time – that may extend beyond fungi and merits explicit tests in other mutualist systems. Notably, this accumulation did not translate in our study into convergence with native tree communities, as symbiotic communities of park‐alien trees and co‐occurring natives did not become more similar with increasing residence time, suggesting that integration proceeds along fungal guild‐specific pathways rather than toward full community homogenization.

By contrast, pathogens exhibit a general decline in their relative richness and abundance over time in both AM and ECM alien tree hosts – a pattern that strongly supports the broader notion that residence time is a critical factor in alien plant naturalization (Pyšek *et al*., 2009; Dawson *et al*., 2017) and runs counter to the canonical expectation of enemy accumulation (Keane & Crawley, 2002; Mitchell *et al*., 2010; Flory & Clay, 2013). A more nuanced trajectory is therefore likely, in which progressive integration into local mutualist networks buffers pathogen colonization, especially for ECM trees. In addition, several non‐exclusive mechanisms may contribute: (1) age‐ or trait‐linked induction of defences that reduce effective pathogen colonization (e.g. high constitutive defences in long‐lived conifers; Bentz *et al*., 2017); (2) survivorship and management filtering that removes highly susceptible individuals in parks; and (3) introduction bias favouring hardy, pest‐tolerant taxa and cultivars (van Kleunen *et al*., 2018). Over longer timescales, local pathogens are expected to evolve host‐specific adaptations that enable them to effectively colonize and harm newly introduced alien trees (Keane & Crawley, 2002; Stricker *et al*., 2016). Thus, even if initial pathogen loads decline, smaller yet increasingly specialized populations may still constrain naturalization, and further work is needed to resolve the balance between progressive mutualistic benefits and specialized local pathogen adaptation. Metabarcoding of fungal communities does not provide absolute abundance; read counts are compositional. Thus, whether the observed decrease in pathogens in alien tree–associated fungal communities reflects solely an increase in ECM fungi or an actual reduction in pathogen biomass remains unclear. Nevertheless, our results show a clear shift from pathogen‐dominated to mycorrhizal‐dominated communities during the post‐introduction phase of alien tree species in novel regions.

Focusing on the mycorrhizal type, our results indicate that inherent differences between ECM and AM symbioses further shape the balance between mutualistic and pathogenic fungi associated with alien trees. AM alien trees exhibited higher overall pathogen relative richness and abundance than ECM alien trees (Fig. 5a,c), presumably because their AM associations – characterized by predominantly intracellular colonization and limited extraradical hyphal development – offer fewer physical barriers against pathogen invasion (Smith & Read, 2008; Eagar *et al*., 2022) whereas ectomycorrhizal fungi envelop roots in a protective sheath and release antifungal compounds (Smith & Read, 2008; van der Heijden *et al*., 2015) that hinder pathogen ingress. We note that ITS2 under‐detects AM fungi relative to ECM fungi; accordingly, our analyses of mycorrhizal fungi were restricted to ECM fungi and to alien hosts that are ECM. By contrast, analyses of pathogenic fungi included both AM‐ and ECM‐associated alien trees. We also verified that pathogen‐model inferences are robust to potential AM under‐detection (Table S4). Taken together, our results suggest that the timing of alien tree introductions (residence time) and their mode of association with mycorrhizal fungi (mycorrhizal type) may both affect resource uptake and pathogen interactions, with important implications for alien tree post‐introduction trajectories. Understanding these interacting processes is critical for developing risk assessment and monitoring to manage alien tree introductions and mitigate their impacts on native ecosystems.

Our comparative analyses revealed clear fungal trophic guild‐level shifts between alien and native tree assemblages, both inside parks and in adjacent forests. Park‐alien tree assemblages hosted fewer ECM fungal species than native tree assemblages, although ECM fungal abundance was similar across groups (Fig. 6a,b). By contrast, pathogen richness was twice as high, and pathogen abundance even three times higher in alien tree assemblages compared to native tree ones. Although pathogen richness and abundance declined with residence time at the individual tree level, park‐alien tree assemblages still supported elevated pathogen loads overall (Fig. 6c,d). These findings are consistent with hypotheses and mechanisms discussed in the literature, often framed within invasion ecology, suggesting that the introduction of alien plants can disrupt soil microbial communities and alter competitive dynamics, potentially favouring pathogens and amplifying impacts on native ecosystems (Ehrenfeld, 2003; Callaway & Ridenour, 2004; Desprez‐Loustau *et al*., 2007; Simberloff *et al*., 2013). The gradient we observed – from highest pathogen loads in alien tree assemblages, through park‐native tree patches, to lowest loads in native forests – points to density‐driven host–pathogen feedbacks, whereby prolific alien hosts amplify local inoculum that spills over into neighbouring vegetation (Bever *et al*., 2015; Parker *et al*., 2015; Gilbert & Parker, 2016). This finding has important management implications, as the accumulation of pathogens in alien vegetation may facilitate spillover into adjacent native ecosystems, thereby elevating disease risks and triggering outbreaks of emerging pathogens (Daszak *et al*., 2000; Anderson *et al*., 2004).

Despite our rigorous approach, extensive replication, and broad taxonomic coverage of alien trees in assessing their belowground fungal interactions, several limitations remain. First, our observational design limits the possibility of making causal inferences. Although we identified robust associations among alien tree origin, mycorrhizal type, residence time, and the structure and composition of symbiotic fungal communities, controlled experiments are needed to disentangle the effects of host‐associated and environmental factors and to establish causality. Second, assigning fungi to trophic guilds is complicated by the functional plasticity of some groups – mainly saprotrophs and certain pathogens – and by gaps in ecological knowledge (Nguyen *et al*., 2016; Põlme *et al*., 2020; Tanunchai *et al*., 2023). For this reason, we only classified fungal SHs as ‘pathogen’ when pathogenic status was well supported; ambiguous or facultative taxa were left unassigned. As a result, the pathogen richness and abundance we report should be viewed as conservative estimates of pathogenic richness and abundance. This is less of a concern for ECM and AM fungi, whose ecological roles are comparatively well characterized (Treseder & Lennon, 2015; Nguyen *et al*., 2016). In addition, a sizeable fraction of SHs lacked a confident trophic‐guild assignment. We intentionally excluded these unassigned taxa from guild‐based proportions to avoid false positives. Genus‐level trait assignment is a common practice in environmental ITS surveys and reflects evidence that many fungal functional traits – and thus trophic roles – are conserved at the genus level (Nguyen *et al*., 2016; Põlme *et al*., 2020; Zanne *et al*., 2020). Third, while pooling soil samples from alien trees into composite ‘park‐alien tree assemblage’ units enabled broad landscape‐level analyses, it may also potentially obscure the heterogeneity among individual trees and microhabitats that typically characterizes natural vegetation and underpins fine‐scale fungal interactions. Fourth, although dual mycorrhizal associations have been reported for some tree taxa (Teste *et al*., 2020), our host tree list did not include any taxa classified as mixed/dual ECM–AM in the reference we used (Soudzilovskaia *et al*., 2020); therefore, potential dual colonization is unlikely to affect our AM vs ECM comparisons. Finally, because our sampling represents only a snapshot in time, longitudinal studies will be essential to reveal how symbiotic fungi and pathogens shift during the post‐introduction residence of alien plants.

### Conclusions

The assembly of belowground symbiotic fungal communities associated with alien tree species is primarily driven by the interplay between residence time and mycorrhizal type, with the biogeographical origin and local environmental factors further shaping their composition, structure, and dynamics. From a conservation perspective, prioritizing risk assessment for tree introductions from distant regions, and rigorously monitoring pathogen outbreaks – particularly in newly established alien tree stands, and in their vicinity – may help safeguard native forest resilience and ecosystem functions. We propose giving special attention to monitoring the naturalization risk of alien ECM trees, which tend to accumulate ECM fungal mutualists with longer residence times, potentially strengthening their fitness and aiding naturalization, although the drivers of this accumulation are still unknown. Our findings underscore that integrating belowground and aboveground processes is essential for developing holistic models of post‐introduction dynamics under global change (van der Putten *et al*., 2009; Vestergård *et al*., 2015; Dickie *et al*., 2017), emphasizing that a comprehensive understanding of belowground plant–fungal symbioses is pivotal for predicting, mitigating, and managing the ecological impacts of alien plant introductions. Taken together, our findings substantially advance current knowledge of how alien trees restructure native soil fungal networks by revealing the joint roles of biogeographical origin, residence time and mycorrhizal type.

## Competing interests

None declared.

## Author contributions

LV, PK and Petr Pyšek conceived and designed the study. LV, CK, MP, MV, JS and Petr Petřík performed the terrain surveys and soil sampling in chateau parks. JK and PB performed the molecular work and contributed to sequencing. TV processed sequencing data and conducted bioinformatics. LV, PK, IO, TV, JP and PB analysed the data and interpreted the results. LV wrote the first draft of the manuscript, and all authors contributed to revisions and approved the final manuscript. Petr Pyšek and PK contributed equally to this work.

## Disclaimer

The New Phytologist Foundation remains neutral with regard to jurisdictional claims in maps and in any institutional affiliations.

## Supporting information


**Fig. S1** Map of sampling locations across the 48 Czech chateau parks.
**Fig. S2** Representative photos of sampling locations and tree assemblage types.
**Fig. S3** Sample‐based rarefaction curves of fungal SH richness across tree assemblage types, shown separately for ectomycorrhizal fungi and putative plant pathogens.
**Fig. S4** Top 15 genera of ectomycorrhizal fungi and putative plant‐pathogenic fungi detected in soil, shown as relative read abundance.
**Table S1** List of the 48 Czech chateau parks sampled and park identity numbers used in analyses.
**Table S2** Alien tree sample metadata for 520 soil samples, including host traits, residence time, environmental covariates and GPS.
**Table S3** Native assemblage metadata for park and forest plots (76 composite samples), including environmental covariates and GPS.
**Table S4** Robustness of pathogen GLMMs to AM fungal under‐detection: results after excluding AM fungal taxa from denominators.
**Table S5** Contingency tables and sensitivity analyses addressing dependence among biogeographical origin, mycorrhizal type and plant group.
**Table S6** Full marginal PERMANOVA results for drivers of alien tree fungal community composition.
**Table S7** Taxonomic composition of unassigned SHs lacking a confident trophic‐guild assignment.
**Table S8** Blocked PERMANOVA comparing fungal community composition across park‐alien, park‐native and forest‐native assemblages (including pairwise tests).
**Table S9** Binomial GLMM outputs for ECM and pathogen relative richness and abundance across tree assemblages.
Please note: Wiley is not responsible for the content or functionality of any Supporting Information supplied by the authors. Any queries (other than missing material) should be directed to the *New Phytologist* Central Office.

## Data Availability

Raw ITS2 reads have been deposited in the NCBI SRA under BioProject PRJNA1291651. All R scripts and processed data needed to reproduce the analyses are archived on Zenodo (doi: 10.5281/zenodo.17607238).
