# Supplementary material for: From pathogens to partners: temporal and biogeographical patterns in fungal associations of alien trees
Source: New Phytol. 2026 Mar 26;250(5):3381–95. doi: 10.1111/nph.71094 (PMC13150302; doi:10.1111/nph.71094)
Supplement: Supplementary file 1 — Fig. S1 Map of sampling locations across the 48 Czech chateau parks. Fig. S2 Representative photos of sampling locations and tree assemblage types. Fig. S3 Sample‐based rarefaction curves of fungal SH richness across tree assemblage types, shown separately for ectomycorrhizal fungi and putative plant pathogens. Fig. S4 Top 15 genera of ectomycorrhizal fungi and putative plant‐pathogenic fungi detected in soil, shown as relative read abundance. Table S1 List of the 48 Czech chateau parks sampled and park identity numbers used in analyses. Table S2 Alien tree sample metadata for 520 soil samples, including host traits, residence time, environmental covariates and GPS. Table S3 Native assemblage metadata for park and forest plots (76 composite samples), including environmental covariates and GPS. Table S4 Robustness of pathogen GLMMs to AM fungal under‐detection: results after excluding AM fungal taxa from denominators. Table S5 Contingency tables and sensitivity analyses addressing dependence among biogeographical origin, mycorrhizal type and plant group. Table S6 Full marginal PERMANOVA results for drivers of alien tree fungal community composition. Table S7 Taxonomic composition of unassigned SHs lacking a confident trophic‐guild assignment. Table S8 Blocked PERMANOVA comparing fungal community composition across park‐alien, park‐native and forest‐native assemblages (including pairwise tests). Table S9 Binomial GLMM outputs for ECM and pathogen relative richness and abundance across tree assemblages. Please note: Wiley is not responsible for the content or functionality of any Supporting Information supplied by the authors. Any queries (other than missing material) should be directed to the New Phytologist Central Office. [file NPH-250-3381-s001.pdf]

New Phytologist Supporting Information

Article title: From pathogens to partners: Temporal and biogeographical patterns in fungal associations of alien trees

Authors: Lukáš Vlk, Iñaki Odriozola, Jan Pergl, Tomáš Větrovský, Jana Kvasničková, Claudia Krüger, Markéta Petružálková, Petr Baldrian, Martin Vojík, Jiří Sádlo, Petr Petřík, Petr Pyšek, Petr Kohout

Article acceptance date: 18 February 2026

Figure S1

No.	Code	Name
1	AZ	Americká zahrada
2	BH	Bílá Lhota
3	BLUD	Bludov
4	CK	Ceský Krumlov
5	CR	Chrast
6	DB	Dobříš
7	DO	Doksany
8	HL	Hluboká nad Vltavou
9	HN	Hrádek u Nechanic
10	HT	Horšovský Týn
11	CHO	Jirkov
12	JE	Jemniště
13	KA	Kačina
14	KD	Krásný dvůr
15	KK	Karlova Koruna
16	KNO	Klášterec nad Ohří
17	KO	Konopiště
18	KP	Kopidlno
19	KROM	Kroměříž
20	KYN	Kynžvart
21	KZ	Kozel
22	LDN	Lednice
23	LI	Libochovice
24	LIB	Liběchov
25	LL	Lysá nad Labem
26	LO	Loučeň
27	ME	Měšice
28	NH	Nové Hrady
29	NOSV	Nový Světlov
30	OR	Orlík nad Vltavou
31	PASK	Paskov
32	PB	Panenské Břežany
33	PL	Ploskovice
34	PR	Průhonice
35	SL	Slatiňany
36	SYCH	Sychrov
37	TE	Telč
38	VE	Veltrusy
39	VEHE	Velké Heraltice
40	VELO	Velké Losiny
41	VES	Veselíčko
42	VJ	Vrchotovy Janovice
43	VL	Vlašim
44	VN	Vinoř
45	VR	Vráž
46	ZAH	Zahrádky u Ceské Lípy
47	ZB	Zbiroh
48	ZH	Zehušice

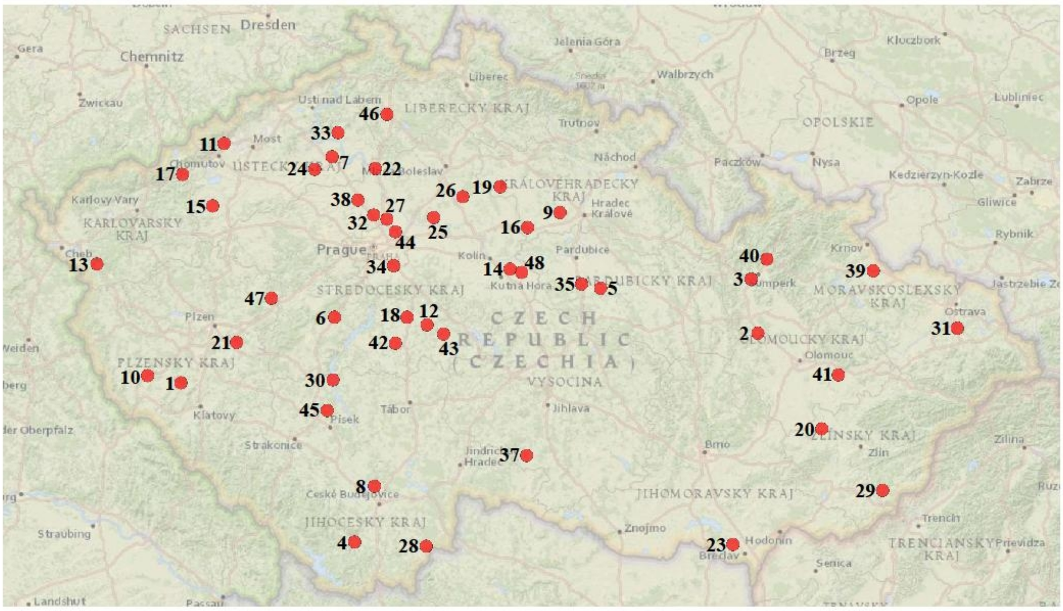

Supplementary Figure S1: Map of sampling locations

Figure S2

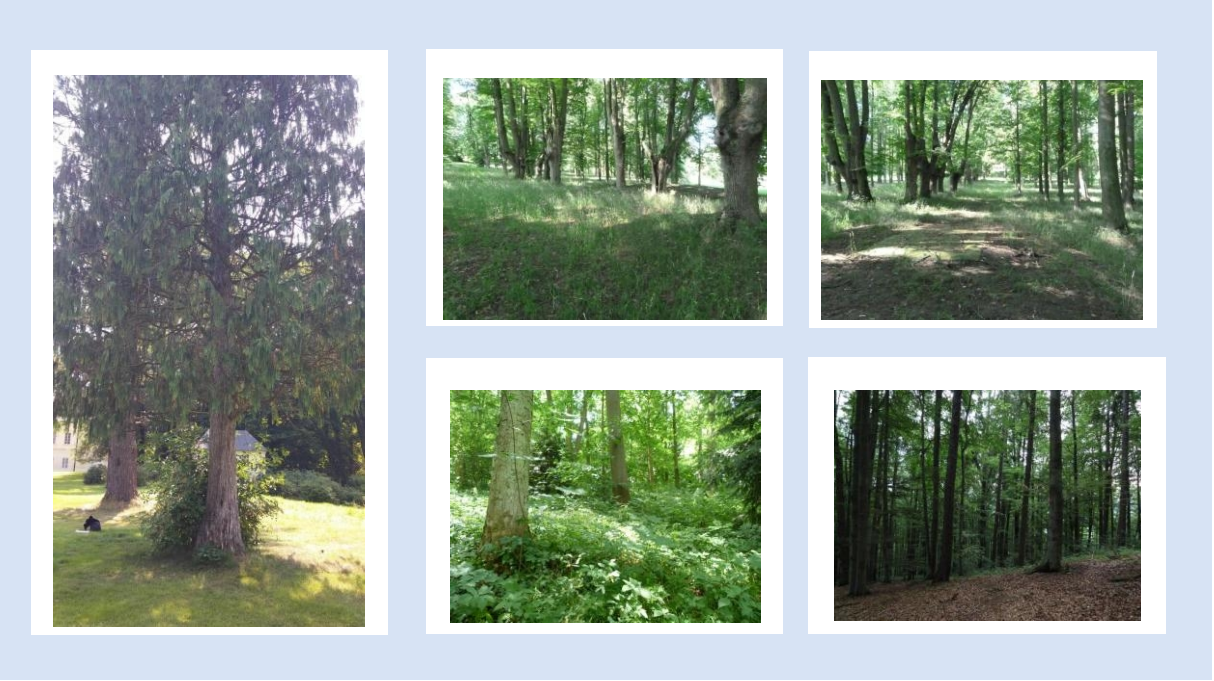

Supplementary Figure S2: Representative photos of sampling locations and tree assemblage types. (A) Solitary non-native tree (Chamaecyparis lawsoniana) sampled in Vlašim Chateau Park. (B) Park-native tree assemblage, Kozel Chateau Park. (C) Park-native tree assemblage, Konopiště Chateau Park. (D) Forest-native tree assemblage near Vrchotovy Janovice Chateau Park. (E) Forest-native tree assemblage near Velké Losiny Chateau Park. Photos: (A) Petr Kohout; (B–E) Petr Petřík.

Figure S3

Sample-based rarefaction (species accumulation) curves of fungal SH richness across tree assemblage types

Ectomycorrhizal fungi

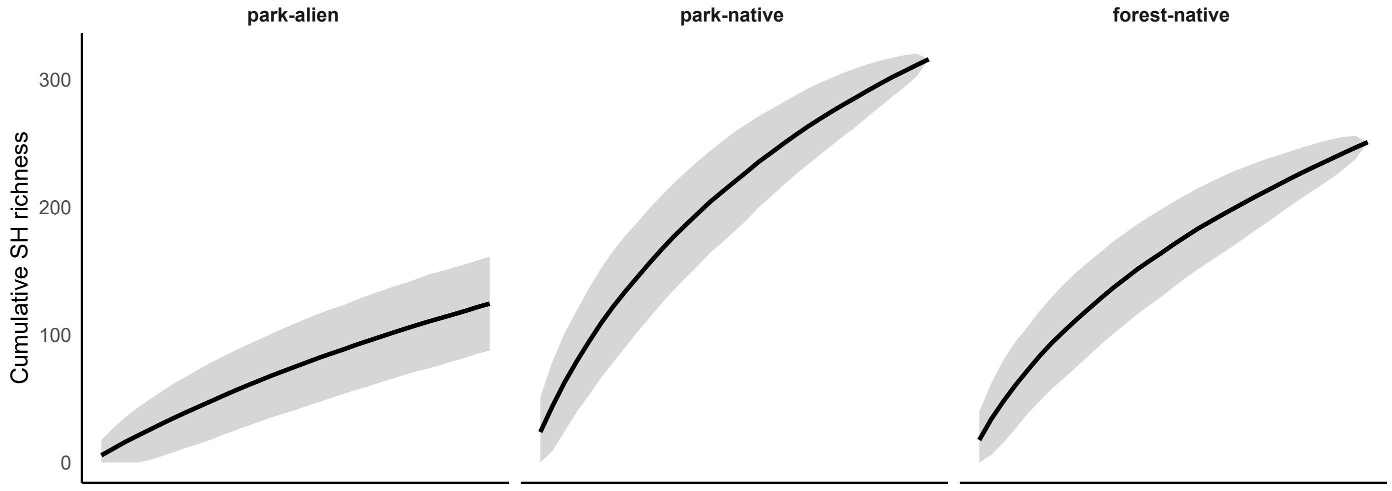

Putative plant pathogens

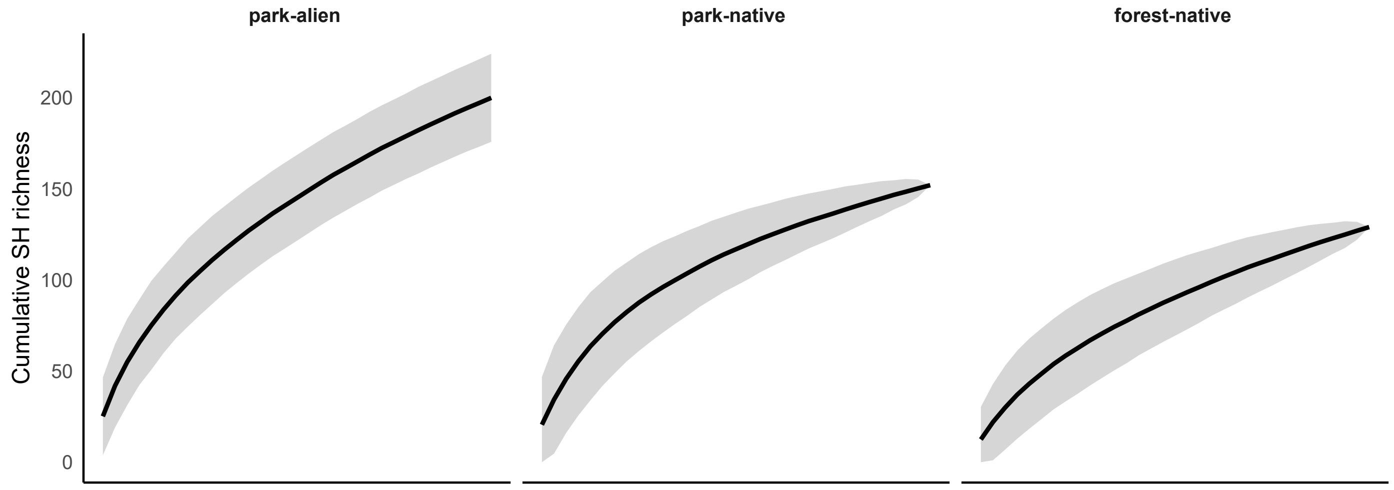

Supplementary Figure S3: Sample-based rarefaction (species accumulation) curves of fungal SH richness across tree assemblage types (park-alien, park-native, forest-native), shown separately for ectomycorrhizal fungi and putative plant pathogens. Here, ‘samples’ correspond to individual soil samples within each tree assemblage type. Curves were computed from SH presence/absence data using randomised sample-order permutations (999 permutations; function specaccum, method = “random”), with shaded bands indicating 95% confidence intervals. To standardise sampling effort across assemblage types, curves are truncated to the minimum number of samples available among groups.

Figure S4

ECM fungi

Fungal genus	Relative read abundance (% of symbiotic reads)
Russula	11.22367663
Inocybe	9.565643121
Tomentella	2.153981146
Sebacina	2.005196853
Scleroderma	1.252903625
Lactarius	1.001538709
Tuber	0.912204551
Amphinema	0.82212859
Hebeloma	0.723680864
Hymenogaster	0.565888976
Amanita	0.559212757
Cortinarius	0.458751568
Piloderma	0.45271118
Xerocomellus	0.435649734
Clavulina	0.419648004

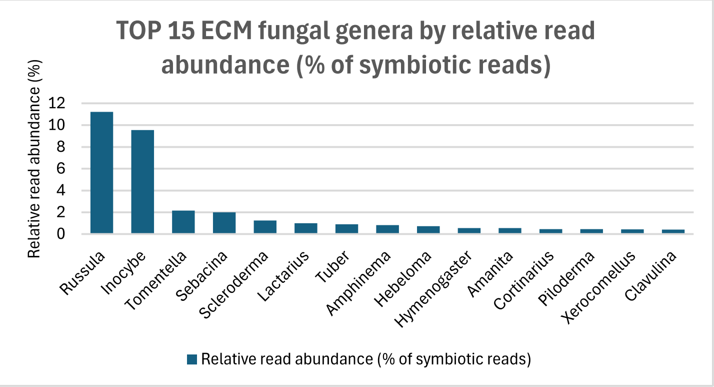

Putative plant pathogens

Fungal genus	Relative read abundance (% of symbiotic reads)
Fusarium	14.93872715
Alternaria	7.550590898
Epicoccum	6.69094832
Neonectria	1.785517481
Phoma	1.687811557
Calophoma	1.504904371
Neoascochyta	0.971760657
Ganoderma	0.924391298
Diaporthe	0.888466886
Paraphoma	0.777302553
Laetiporus	0.719653939
Gibellulopsis	0.694326698
Discosia	0.630743667
Didymella	0.626080912
Cylindrocarpon	0.504743294

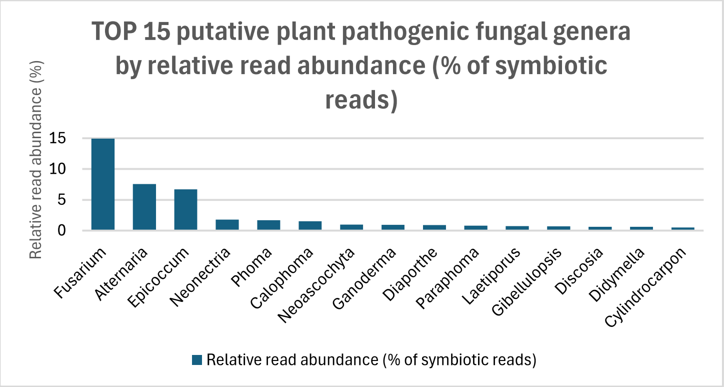

Supplementary Figure S4: Top 15 genera of ectomycorrhizal (ECM) fungi and putative plant pathogenic fungi detected in soil. Bars show relative read abundance (% of symbiotic reads) for each genus. Percentages are calculated from all ITS2 reads assigned to symbiotic fungal guilds; plots show only ECM and plant pathogen guilds. Read counts were summed across all UNITE SHs assigned to the same genus.

Table S1

Code	No.	Name	Native tree assemblage
AZ	1	Americká zahrada	No
BH	2	Bílá Lhota	Yes
BLUD	3	Bludov	No
CK	4	Český Krumlov	No
CR	5	Chrast	No
DB	6	Dobříš	No
DO	7	Doksany	No
HL	8	Hluboká nad Vltavou	Yes
HN	9	Hrádek u Nechanic	No
HT	10	Horšovský Týn	Yes
CHO	11	Jirkov	No
JE	12	Jemniště	Yes
KA	13	Kačina	Yes
KD	14	Krásný dvůr	Yes
KK	15	Karlova Koruna	Yes
KNO	16	Klášterec nad Ohří	Yes
KO	17	Konopiště	Yes
KP	18	Kopidlno	Yes
KROM	19	Kroměříž	Yes
KYN	20	Kynžvart	Yes
KZ	21	Kozel	Yes
LB	22	Liběchov	No
LED	23	Lednice	Yes
LI	24	Libochovice	Yes
LL	25	Lysá nad Labem	No
LO	26	Loučeň	Yes
ME	27	Měšice	No
NH	28	Nové Hrady	Yes
NOSV	29	Nový Světlov	Yes
OR	30	Orlík nad Vltavou	Yes
PASK	31	Paskov	Yes
PB	32	Panenské Břežany	Yes
PL	33	Ploskovice	Yes
PR	34	Průhonice	Yes
SL	35	Slatiňany	No
SYCH	36	Sychrov	No

TE	37	Telč	No
VE	38	Veltrusy	Yes
VEHE	39	Velké Heraltice	Yes
VELO	40	Velké Losiny	Yes
VES	41	Veselíčko	Yes
VJ	42	Vrchotovy Janovice	Yes
VL	43	Vlašim	Yes
VN	44	Vinoř	Yes
VR	45	Vráž	No
ZAH	46	Zahrádky u České Lípy	Yes
ZB	47	Zbiroh	Yes
ZH	48	Žehušice	Yes

Supplementary Table S1: List of the 48 Czech chateau parks sampled and park identity numbers used in analyses.

Table S2

sample_ID	total_sps.sym	total_seq.sym	ecm_sps	ecm_seq	ecm_share_sps.sym	ecm_share.sym	patho_sps	patho_seq	patho_share_sps.sym	patho_share.sym	park	park_nr	pl_species	pl_species_age_cr	pl_genus	pl_family	pl_order	pl_origin_cont	plant_type	latitude	longitude	m_type	mat	map	area	alien.treeSR	altitude	ecm.s	angio.s
AZ01	37	2792	13	2631	0.351351351	0.942335244	7	37	0.189189189	0.013252149	AZ	1	Tsuga_canadensis	1812	Tsuga	Pinaceae	Pinales	namerica	gymno	49.4614125	13.15831028	ecm	77	809	16288	6	515	0.714285714	0.142857143
AZ02	31	436	6	128	0.193548387	0.293577982	8	53	0.258064516	0.121559633	AZ	1	Pseudotsuga_menziesii	1830	Pseudotsuga	Pinaceae	Pinales	namerica	gymno	49.46147972	13.15861	ecm	77	809	16288	6	515	0.714285714	0.142857143
AZ03	16	546	3	411	0.1875	0.752747253	4	57	0.25	0.104395604	AZ	1	Abies_grandis	1879	Abies	Pinaceae	Pinales	namerica	gymno	49.46113917	13.15849528	ecm	77	809	16288	6	515	0.714285714	0.142857143
AZ04	40	1144	16	813	0.4	0.710664336	8	106	0.2	0.092657343	AZ	1	Sequoiadendron_giganteum	1859	Sequoiadendron	Cupressaceae	Pinales	namerica	gymno	49.46123278	13.15812778	am	80	814	16288	6	515	0.714285714	0.142857143
AZ05	41	1228	12	1061	0.292682927	0.864006515	9	40	0.219512195	0.03257329	AZ	1	Thuja_plicata	1844	Thuja	Cupressaceae	Pinales	namerica	gymno	49.46119917	13.15817667	am	80	814	16288	6	515	0.714285714	0.142857143
AZ07	35	1740	6	1487	0.171428571	0.854597701	18	168	0.514285714	0.096551724	AZ	1	Pseudotsuga_menziesii	1830	Pseudotsuga	Pinaceae	Pinales	namerica	gymno	49.46193333	13.15764472	ecm	80	814	16288	6	515	0.714285714	0.142857143
AZ10	20	521	10	463	0.5	0.888675624	5	27	0.25	0.051823417	AZ	1	Carya_tomentosa	1865	Carya	Juglandaceae	Fagales	namerica	angio	49.46230861	13.15774333	ecm	80	814	16288	6	515	0.714285714	0.142857143
BH01	45	579	1	3	0.022222222	0.005181347	32	508	0.711111111	0.877374784	BH	2	Pterocarya_fraxinifolia	1844	Pterocarya	Juglandaceae	Fagales	asia	angio	49.70951278	16.97619194	am	90	607	25161	13	320	0.461538462	0.538461538
BH02	46	3356	16	2496	0.347826087	0.743742551	18	506	0.391304348	0.150774732	BH	2	Castanea_sativa	1562	Castanea	Fagaceae	Fagales	europe	angio	49.70966083	16.97615778	ecm	90	607	25161	13	320	0.461538462	0.538461538
BH03	49	2170	1	3	0.020408163	0.001382488	36	2012	0.734693878	0.92718894	BH	2	Gleditsia_triacanthos	1785	Gleditsia	Fabaceae	Fabales	namerica	angio	49.70986278	16.97723583	am	90	607	25161	13	320	0.461538462	0.538461538
BH04	39	779	0	0	0	0	29	719	0.743589744	0.922978177	BH	2	Celtis_occidentalis	1835	Celtis	Cannabaceae	Rosales	namerica	angio	49.70955389	16.97773583	am	90	607	25161	13	320	0.461538462	0.538461538
BH05	35	569	2	45	0.057142857	0.079086116	27	494	0.771428571	0.868189807	BH	2	Tsuga_canadensis	1812	Tsuga	Pinaceae	Pinales	namerica	gymno	49.70989778	16.97758583	ecm	90	607	25161	13	320	0.461538462	0.538461538
BH06	53	751	0	0	0	0	42	651	0.79245283	0.866844208	BH	2	Picea_pungens	1910	Picea	Pinaceae	Pinales	namerica	gymno	49.70998083	16.97742389	ecm	90	607	25161	13	320	0.461538462	0.538461538
BH07	47	876	3	101	0.063829787	0.115296804	30	635	0.638297872	0.724885845	BH	2	Abies_concolor	1910	Abies	Pinaceae	Pinales	namerica	gymno	49.70999889	16.97737194	ecm	90	607	25161	13	320	0.461538462	0.538461538
BH08	46	1005	13	596	0.282608696	0.593034826	24	356	0.52173913	0.354228856	BH	2	Cryptomeria_japonica	1845	Cryptomeria	Cupressaceae	Pinales	asia	gymno	49.71047	16.97685083	am	90	607	25161	13	320	0.461538462	0.538461538
BH09	47	488	4	58	0.085106383	0.118852459	29	351	0.617021277	0.719262295	BH	2	Liriodendron_tulipifera	1785	Liriodendron	Altingiaceae	Saxifragales	namerica	angio	49.71031889	16.97620083	am	90	607	25161	13	320	0.461538462	0.538461538
BH10	51	1626	5	964	0.098039216	0.592865929	29	502	0.568627451	0.308733087	BH	2	Pinus_ponderosa	1845	Pinus	Pinaceae	Pinales	namerica	gymno	49.71054583	16.97584583	ecm	90	607	25161	13	320	0.461538462	0.538461538
BH11	32	1692	3	21	0.09375	0.012411348	15	264	0.46875	0.156028369	BH	2	Pseudotsuga_menziesii	1830	Pseudotsuga	Pinaceae	Pinales	namerica	gymno	49.71046694	16.97542	ecm	90	607	25161	13	320	0.461538462	0.538461538
BH12	33	861	0	0	0	0	23	398	0.696969697	0.462253194	BH	2	Catalpa_bignonioides	1785	Catalpa	Bignoniaceae	Lamiales	namerica	angio	49.71012278	16.976195	am	90	607	25161	13	320	0.461538462	0.538461538
BH13	53	1232	14	408	0.264150943	0.331168831	27	745	0.509433962	0.604707792	BH	2	Magnolia_x_soulangeana	1844	Magnolia	Magnoliaceae	Magnoliales	hybrid	angio	49.71007194	16.97635694	am	90	607	25161	13	320	0.461538462	0.538461538
BLUD01	41	2307	1	7	0.024390244	0.003034244	25	2176	0.609756098	0.943216298	BLUD	3	Liriodendron_tulipifera	1785	Liriodendron	Altingiaceae	Saxifragales	namerica	angio	49.93946389	16.92946194	am	87	682	43096	7	300	0.625	0.375
BLUD02	36	431	6	83	0.166666667	0.192575406	19	301	0.527777778	0.69837587	BLUD	3	Pinus_ponderosa	1845	Pinus	Pinaceae	Pinales	namerica	gymno	49.939315	16.93001583	ecm	87	682	43096	7	300	0.625	0.375
BLUD03	35	437	0	0	0	0	24	377	0.685714286	0.862700229	BLUD	3	Platanus_×_acerifolia	1835	Platanus	Platanaceae	Proteales	hybrid	angio	49.93914583	16.930115	am	87	682	43096	7	300	0.625	0.375
BLUD04	45	1732	7	167	0.155555556	0.096420323	26	1440	0.577777778	0.831408776	BLUD	3	Pinus_ponderosa	1845	Pinus	Pinaceae	Pinales	namerica	gymno	49.93832389	16.92947694	ecm	87	682	43096	7	300	0.625	0.375
BLUD05	46	1085	1	98	0.02173913	0.090322581	29	840	0.630434783	0.774193548	BLUD	3	Pseudotsuga_menziesii	1830	Pseudotsuga	Pinaceae	Pinales	namerica	gymno	49.93815	16.92932	ecm	87	682	43096	7	300	0.625	0.375
BLUD06	38	832	5	475	0.131578947	0.570913462	25	316	0.657894737	0.379807692	BLUD	3	Cedrus_libani	1812	Cedrus	Pinaceae	Pinales	asia	gymno	49.93826583	16.929345	ecm	87	682	43096	7	300	0.625	0.375
BLUD07	58	4151	0	0	0	0	41	3969	0.706896552	0.956155143	BLUD	3	Gleditsia_triacanthos	1785	Gleditsia	Fabaceae	Fabales	namerica	angio	49.94037583	16.92985778	am	87	682	43096	7	300	0.625	0.375
BLUD08	43	1363	2	11	0.046511628	0.008070433	28	1276	0.651162791	0.936170213	BLUD	3	Picea_pungens	1910	Picea	Pinaceae	Pinales	namerica	gymno	49.94035778	16.92953194	ecm	87	682	43096	7	300	0.625	0.375
CK01	37	895	1	4	0.027027027	0.004469274	24	823	0.648648649	0.919553073	CK	4	Robinia_pseudoacacia	1710	Robinia	Fabaceae	Fabales	namerica	angio	48.81063389	14.30822444	am	85	594	91887	3	550	0.333333333	0.666666667
CK02	49	1499	7	720	0.142857143	0.480320213	32	621	0.653061224	0.414276184	CK	4	Platanus_occidentalis	1785	Platanus	Platanaceae	Proteales	namerica	angio	48.8107375	14.30830139	am	85	594	91887	3	550	0.333333333	0.666666667
CK03	41	469	9	88	0.219512195	0.187633262	23	337	0.56097561	0.718550107	CK	4	Pinus_strobus	1785	Pinus	Pinaceae	Pinales	namerica	gymno	48.81145083	14.30833778	ecm	85	594	91887	3	550	0.333333333	0.666666667
CR01	49	1007	2	62	0.040816327	0.061569017	34	882	0.693877551	0.875868918	CR	5	Pinus_strobus	1785	Pinus	Pinaceae	Pinales	namerica	gymno	49.90058083	15.93836	ecm	89	610	24188	15	284	0.476190476	0.380952381
CR02	54	907	2	9	0.037037037	0.009922822	33	622	0.611111111	0.685777288	CR	5	Chamaecyparis_nootkatensis	1863	Chamaecyparis	Cupressaceae	Pinales	namerica	gymno	49.90050944	15.93844278	am	89	610	24188	15	284	0.476190476	0.380952381
CR03	58	2388	4	98	0.068965517	0.041038526	37	2173	0.637931034	0.909966499	CR	5	Tsuga_canadensis	1812	Tsuga	Pinaceae	Pinales	namerica	gymno	49.9005125	15.93826278	ecm	89	610	24188	15	284	0.476190476	0.380952381
CR04	71	5872	1	3	0.014084507	0.000510899	44	5562	0.61971831	0.947207084	CR	5	Fraxinus_pennsylvanica	1835	Fraxinus	Oleaceae	Lamiales	namerica	angio	49.90013361	15.93897583	am	89	610	24188	15	284	0.476190476	0.380952381
CR05	51	773	1	7	0.019607843	0.009055627	34	623	0.666666667	0.805950841	CR	5	Thuja_plicata	1844	Thuja	Cupressaceae	Pinales	namerica	gymno	49.90024944	15.93922111	am	89	610	24188	15	284	0.476190476	0.380952381
CR06	61	4601	1	6	0.016393443	0.001304064	48	4380	0.786885246	0.951966964	CR	5	Castanea_sativa	1562	Castanea	Fagaceae	Fagales	europe	angio	49.90005139	15.93937611	ecm	89	610	24188	15	284	0.476190476	0.380952381
CR07	41	1282	11	773	0.268292683	0.602964119	25	449	0.609756098	0.350234009	CR	5	Abies_concolor	1910	Abies	Pinaceae	Pinales	namerica	gymno	49.89999389	15.9395525	ecm	89	610	24188	15	284	0.476190476	0.380952381
CR08	29	431	2	11	0.068965517	0.025522042	24	405	0.827586207	0.939675174	CR	5	Sophora_japonica	1835	Sophora	Fabaceae	Fabales	asia	angio	49.89991167	15.93957444	am	89	610	24188	15	284	0.476190476	0.380952381
CR09	39	1164	1	640	0.025641026	0.549828179	30	479	0.769230769	0.411512027	CR	5	Gleditsia_triacanthos	1785	Gleditsia	Fabaceae	Fabales	namerica	angio	49.89970833	15.93959917	am	89	592	24188	15	284	0.476190476	0.380952381
CR10	41	1382	0	0	0	0	33	1315	0.804878049	0.951519537	CR	5	Gleditsia_triacanthos	1785	Gleditsia	Fabaceae	Fabales	namerica	angio	49.89968056	15.93929278	am	89	592	24188	15	284	0.476190476	0.380952381
CR11	27	184	2	8	0.074074074	0.043478261	17	138	0.62962963	0.75	CR	5	Pseudotsuga_menziesii	1830	Pseudotsuga	Pinaceae	Pinales	namerica	gymno	49.89969833	15.93980556	ecm	89	592	24188	15	284	0.476190476	0.380952381
CR12	66	3353	7	222	0.106060606	0.066209365	41	2886	0.621212121	0.860721742	CR	5	Pseudotsuga_menziesii	1830	Pseudotsuga	Pinaceae	Pinales	namerica	gymno	49.89963306	15.93971056	ecm	89	592	24188	15	284	0.476190476	0.380952381
CR13	102	5773	18	920	0.176470588	0.15936255	55	4302	0.539215686	0.74519314	CR	5	Castanea_sativa	1562	Castanea	Fagaceae	Fagales	europe	angio	49.89909667	15.9405	ecm	89	592	24188	15	284	0.476190476	0.380952381
CR14	59	1616	7	119	0.118644068	0.073638614	36	995	0.610169492	0.615717822	CR	5	Picea_pungens	1910	Picea	Pinaceae	Pinales	namerica	gymno	49.89972056	15.93877694	ecm	89	592	24188	15	284	0.476190476	0.380952381
CR15	67	3405	6	997	0.089552239	0.292804699	47	1837	0.701492537	0.539500734	CR	5	Picea_pungens	1910	Picea	Pinaceae	Pinales	namerica	gymno	49.89985778	15.93909972	ecm	89	592	24188	15	284	0.476190476	0.380952381
CR16	38	3148	0	0	0	0	28	3045	0.736842105	0.967280813	CR	5	Taxodium_distichum	1835	Taxodium	Cupressaceae	Pinales	namerica	gymno	49.89977583	15.93868306	am	89	592	24188	15	284	0.476190476	0.380952381
CR17	46	3520	0	0	0	0	33	2773	0.717391304	0.787784091	CR	5	Ginkgo_biloba	1809	Ginkgo	Ginkgoaceae	Ginkgoales	asia	gymno	49.89996417	15.93842639	am	89	610	24188	15	284	0.476190476	0.380952381
CR19	50	941	5	25	0.1	0.026567481	32	845	0.64	0.897980871	CR	5	Pinus_strobus	1785	Pinus	Pinaceae	Pinales	namerica	gymno	49.90025722	15.93798944	ecm	89	610	24188	15	284	0.476190476	0.380952381
CR20	66	1829	1	9	0.015151515	0.004920722	43	1640	0.651515152	0.896664844	CR	5	Juniperus_virginiana	1812	Juniperus	Cupressaceae	Pinales	namerica	gymno	49.90018444	15.93812306	am	89	610	24188	15	284	0.476190476	0.380952381
CR21	77	2236	19	1206	0.246753247	0.539355993	36	752	0.467532468	0.336314848	CR	5	Magnolia_x_soulangeana	1844	Magnolia	Magnoliaceae	Magnoliales	hybrid	angio	49.90012583	15.93820639	am	89	610	24188	15	284	0.476190476	0.380952381
CR22	61	3677	1	6	0.016393443	0.001631765	40	3371	0.655737705	0.916779984	CR	5	Magnolia_x_soulangeana	1844	Magnolia	Magnoliaceae	Magnoliales	hybrid	angio	49.90051556	15.93791694	am	89	610	24188	15	284	0.476190476	0.380952381
DB01	71	2420	3	139	0.042253521	0.057438017	38	1958	0.535211268	0.809090909	DB	6	Magnolia_acuminata	1844	Magnolia	Magnoliaceae	Magnoliales	namerica	angio	49.77856556	14.17976028	am	86	463	100324	5	365	0.4	0.2
DB04	40	573	10	289	0.25	0.504363002	16	180	0.4	0.314136126	DB	6	Pseudotsuga_menziesii	1830	Pseudotsuga	Pinaceae	Pinales	namerica	gymno	49.77839528	14.18004639	ecm	86	463	100324	5	365	0.4	0.2
DB05	39	882	7	537	0.179487179	0.608843537	20	265	0.512820513	0.300453515	DB	6	Pinus_strobus	1785	Pinus	Pinaceae	Pinales	namerica	gymno	49.77755917	14.18113611	ecm	86	463	100324	5	365	0.4	0.2
DB06	9	68	0	0	0	0	5	28	0.555555556	0.411764706	DB	6	Thuja_occidentalis	1785	Thuja	Cupressaceae	Pinales	namerica	gymno	49.77897278	14.17895306	am	86	463	100324	5	365	0.4	0.2
DB08	37	1217	3	92	0.081081081	0.075595727	23	1005	0.621621622	0.82580115	DB	6	Ginkgo_biloba	1809	Ginkgo	Ginkgoaceae	Ginkgoales	asia	gymno	49.781575	14.17869528	am	86	463	100324	5	365	0.4	0.2
DO03	51	1290	0	0	0	0	38	1156	0.745098039	0.896124031	DO	7	Robinia_pseudoacacia	1710	Robinia	Fabaceae	Fabales	namerica	angio	50.45626806	14.1590325	am	94	491	101027	5	150	0	0.4
DO04	46	1410	0	0	0	0	35	1258	0.760869565	0.892198582	DO	7	Thuja_plicata	1844	Thuja	Cupressaceae	Pinales	namerica	gymno	50.45526361	14.15798778	am	95	481	101027	5	150	0	0.4
DO05	55	2309	3	100	0.054545455	0.043308792	35	1920	0.636363636	0.8315288	DO	7	Platycladus_orientalis	1785	Platycladus	Cupressaceae	Pinales	asia	gymno	50.45523583	14.15817194	am	95	481	101027	5	150	0	0.4
DO06	54	1656	0	0	0	0	30	1403	0.555555556	0.847222222	DO	7	Platanus_×_acerifolia	1835	Platanus	Platanaceae	Proteales	hybrid	angio	50.45442222	14.15816556	am	95	481	101027	5	150	0	0.4
DO07	43	8138	0	0	0	0	34	8058	0.790697674	0.990169575	DO	7	Ginkgo_biloba	1809	Ginkgo	Ginkgoaceae	Ginkgoales	asia	gymno	50.45437389	14.15883139	am	94	491	101027	5	150	0	0.4
HL01	55	2824	0	0	0	0	36	1846	0.654545455	0.65368272	HL	8	Gymnocladus_dioicus	1844	Gymnocladus	Fabaceae	Fabales	namerica	angio	49.05117583	14.44068472	am	88	641	211039	17	440	0.423076923	0.615384615
HL02	41	495	1	5	0.024390244	0.01010101	24	319	0.585365854	0.644444444	HL	8	Thuja_occidentalis	1785	Thuja	Cupressaceae	Pinales	namerica	gymno	49.05212361	14.4401225	am	88	641	211039	17	440	0.423076923	0.615384615
HL03	63	2361	17	1021	0.26984127	0.43244388	20	865	0.317460317	0.366370182	HL	8	Gymnocladus_dioicus	1844	Gymnocladus	Fabaceae	Fabales	namerica	angio	49.05236083	14.44015806	am	88	641	211039	17	440	0.423076923	0.615384615
HL04	77	2141	13	1268	0.168831169	0.592246614	35	596	0.454545455	0.278374591	HL	8	Gymnocladus_dioicus	1844	Gymnocladus	Fabaceae	Fabales	namerica	angio	49.05253444	14.43969778	am	88	641	211039	17	440	0.423076923	0.615384615
HL05	43	711	15	501	0.348837209	0.70464135	12	96	0.279069767	0.135021097	HL	8	Thuja_occidentalis	1785	Thuja	Cupressaceae	Pinales	namerica	gymno	49.05263222	14.43961194	am	88	641	211039	17	440	0.423076923	0.615384615
HL06	48	2547	12	1810	0.25	0.710639969	17	374	0.354166667	0.146839419	HL	8	Picea_pungens	1910	Picea	Pinaceae	Pinales	namerica	gymno	49.05310333	14.43894194	ecm	88	641	211039	17	440	0.423076923	0.615384615
HL07	59	711	7	37	0.118644068	0.052039381	21	329	0.355932203	0.462728551	HL	8	Aesculus_hippocastanum	1576	Aesculus	Sapindaceae	Sapindales	europe	angio	49.05300917	14.43898583	am	88	641	211039	17	440	0.423076923	0.615384615
HL09	55	3752	20	3008	0.363636364	0.801705757	11	85	0.2	0.022654584	HL	8	Picea_pungens	1910	Picea	Pinaceae	Pinales	namerica	gymno	49.05284861	14.43886	ecm	88	641	211039	17	440	0.423076923	0.615384615
HL10	37	1193	22	1027	0.594594595	0.860854987	6	82	0.162162162	0.068734283	HL	8	Pinus_strobus	1785	Pinus	Pinaceae	Pinales	namerica	gymno	49.05355083	14.43836417	ecm	88	641	211039	17	440	0.423076923	0.615384615
HL11	21	170	12	131	0.571428571	0.770588235	2	7	0.095238095	0.041176471	HL	8	Pinus_strobus	1785	Pinus	Pinaceae	Pinales	namerica	gymno	49.05342944	14.43839167	ecm	88	641	211039	17	440	0.423076923	0.615384615
HL12	47	2296	14	1896	0.29787234	0.825783972	10	79	0.212765957	0.034407666	HL	8	Castanea_sativa	1562	Castanea	Fagaceae	Fagales	europe	angio	49.05341194	14.43957417	ecm	88	641	211039	17	440	0.423076923	0.615384615
HL13	39	2339	10	1986	0.256410256	0.849080804	13	70	0.333333333	0.029927319	HL	8	Castanea_sativa	1562	Castanea	Fagaceae	Fagales	europe	angio	49.05340889	14.43995444	ecm	88	641	211039	17	440	0.423076923	0.615384615
HL15	24	520	9	299	0.375	0.575	6	64	0.25	0.123076923	HL	8	Tsuga_canadensis	1812	Tsuga	Pinaceae	Pinales	namerica	gymno	49.05130611	14.44206333	ecm	90	633	211039	17	440	0.423076923	0.615384615
HL16	23	790	5	213	0.217391304	0.269620253	13	518	0.565217391	0.655696203	HL	8	Magnolia_tripetala	1844	Magnolia	Magnoliaceae	Magnoliales	namerica	angio	49.05136111	14.44188278	am	90	633	211039	17	440	0.423076923	0.615384615
HL17	66	2194	9	1262	0.136363636	0.575205105	36	734	0.545454545	0.334548769	HL	8	Carya_ovata	1865	Carya	Juglandaceae	Fagales	namerica	angio	49.05041556	14.44258167	ecm	90	633	211039	17	440	0.423076923	0.615384615
HL18	33	1372	0	0	0	0	24	1266	0.727272727	0.922740525	HL	8	Thuja_plicata	1844	Thuja	Cupressaceae	Pinales	namerica	gymno	49.05033389	14.44263389	am	90	633	211039	17	440	0.423076923	0.615384615
HL19	63	857	4	62	0.063492063	0.072345391	33	327	0.523809524	0.381563594	HL	8	Picea_alcoquiana	1865	Picea	Pinaceae	Pinales	asia	gymno	49.05009722	14.44240528	ecm	90	633	211039	17	440	0.423076923	0.615384615
HL20	55	2176	2	7	0.036363636	0.003216912	36	1669	0.654545455	0.767003676	HL	8	Wisteria_sinensis	1913	Wisteria	Fabaceae	Fabales	asia	angio	49.05011778	14.44270611	am	90	633	211039	17	440	0.423076923	0.615384615

sample_ID	total_sps.sym	total_seq.sym	ecm_sps	ecm_seq	ecm_share_sps.sym	ecm_share.sym	patho_sps	patho_seq	patho_share_sps.sym	patho_share.sym	park	park_nr	pl_species	pl_species_age_cr	pl_genus	pl_family	pl_order	pl_origin_cont	plant_type	latitude	longitude	m_type	mat	map	area	alien.treeSR	altitude	ecm.s	angio.s
HL21	29	1556	0	0	0	0	21	1351	0.724137931	0.868251928	HL	8	Ginkgo_biloba	1809	Ginkgo	Ginkgoaceae	Ginkgoales	asia	gymno	49.05033528	14.44181528	am	90	633	211039	17	440	0.423076923	0.615384615
HL22	63	1014	2	6	0.031746032	0.00591716	32	583	0.507936508	0.57495069	HL	8	Catalpa_bignonioides	1785	Catalpa	Bignoniaceae	Lamiales	namerica	angio	49.05042444	14.44192361	am	90	633	211039	17	440	0.423076923	0.615384615
HL23	44	1559	4	14	0.090909091	0.008980115	19	1131	0.431818182	0.725465042	HL	8	Cladrastis_kentukea	1844	Cladrastis	Fabaceae	Fabales	namerica	angio	49.05049639	14.44183667	am	90	633	211039	17	440	0.423076923	0.615384615
HL24	57	3144	1	3	0.01754386	0.000954198	32	2607	0.561403509	0.829198473	HL	8	Paulownia_tomentosa	1844	Paulownia	Paulowniaceae	Lamiales	asia	angio	49.05064639	14.44163083	am	90	633	211039	17	440	0.423076923	0.615384615
HL25	54	1889	1	7	0.018518519	0.003705664	33	1568	0.611111111	0.830068819	HL	8	Gymnocladus_dioicus	1844	Gymnocladus	Fabaceae	Fabales	namerica	angio	49.05077472	14.44150028	am	88	641	211039	17	440	0.423076923	0.615384615
HL26	43	1567	0	0	0	0	29	1428	0.674418605	0.911295469	HL	8	Platanus_×_acerifolia	1835	Platanus	Platanaceae	Proteales	hybrid	angio	49.05057806	14.44107722	am	88	641	211039	17	440	0.423076923	0.615384615
HL27	29	1496	7	1320	0.24137931	0.882352941	11	88	0.379310345	0.058823529	HL	8	Castanea_sativa	1562	Castanea	Fagaceae	Fagales	europe	angio	49.05092417	14.44052694	ecm	88	641	211039	17	440	0.423076923	0.615384615
HL28	47	6562	15	5526	0.319148936	0.842121304	21	888	0.446808511	0.135324596	HL	8	Castanea_sativa	1562	Castanea	Fagaceae	Fagales	europe	angio	49.05090528	14.44038639	ecm	88	641	211039	17	440	0.423076923	0.615384615
HN01	40	2050	4	1456	0.1	0.710243902	17	341	0.425	0.166341463	HN	9	Pseudotsuga_menziesii	1830	Pseudotsuga	Pinaceae	Pinales	namerica	gymno	50.22390167	15.6681525	ecm	87	704	46423	5	295	0.444444444	0.777777778
HN02	58	3101	7	1469	0.120689655	0.473718155	32	1047	0.551724138	0.337633022	HN	9	Pseudotsuga_menziesii	1830	Pseudotsuga	Pinaceae	Pinales	namerica	gymno	50.22403083	15.66804528	ecm	87	704	46423	5	295	0.444444444	0.777777778
HN04	65	1277	7	688	0.107692308	0.538762725	29	350	0.446153846	0.274079875	HN	9	Catalpa_bignonioides	1785	Catalpa	Bignoniaceae	Lamiales	namerica	angio	50.22412861	15.66788111	am	87	704	46423	5	295	0.444444444	0.777777778
HN05	48	5024	14	4520	0.291666667	0.899681529	18	194	0.375	0.03861465	HN	9	Castanea_sativa	1562	Castanea	Fagaceae	Fagales	europe	angio	50.22392583	15.66700278	ecm	87	704	46423	5	295	0.444444444	0.777777778
HN06	57	3691	8	2372	0.140350877	0.64264427	25	1042	0.438596491	0.282308318	HN	9	Castanea_sativa	1562	Castanea	Fagaceae	Fagales	europe	angio	50.2238975	15.66709861	ecm	87	704	46423	5	295	0.444444444	0.777777778
HN07	59	589	2	6	0.033898305	0.010186757	28	414	0.474576271	0.702886248	HN	9	Platanus_occidentalis	1785	Platanus	Platanaceae	Proteales	namerica	angio	50.22414028	15.66860806	am	87	704	46423	5	295	0.444444444	0.777777778
HN08	51	1852	6	915	0.117647059	0.494060475	20	396	0.392156863	0.213822894	HN	9	Robinia_pseudoacacia	1710	Robinia	Fabaceae	Fabales	namerica	angio	50.22471056	15.66627417	am	87	715	46423	5	295	0.444444444	0.777777778
HN09	25	1375	6	871	0.24	0.633454545	9	130	0.36	0.094545455	HN	9	Robinia_pseudoacacia	1710	Robinia	Fabaceae	Fabales	namerica	angio	50.22483167	15.666125	am	87	715	46423	5	295	0.444444444	0.777777778
HN10	52	2396	7	950	0.134615385	0.396494157	29	1340	0.557692308	0.559265442	HN	9	Platanus_occidentalis	1785	Platanus	Platanaceae	Proteales	namerica	angio	50.22477722	15.66831111	am	87	704	46423	5	295	0.444444444	0.777777778
HT02	52	1393	15	891	0.288461538	0.639626705	23	264	0.442307692	0.189519024	HT	10	Quercus_rubra	1799	Quercus	Fagaceae	Fagales	namerica	angio	49.53159278	12.94265389	ecm	87	722	202078	4	380	1	0.166666667
HT03	33	1262	0	0	0	0	20	1198	0.606060606	0.949286846	HT	10	Abies_concolor	1910	Abies	Pinaceae	Pinales	namerica	gymno	49.531365	12.94224889	ecm	87	722	202078	4	380	1	0.166666667
HT04	39	669	1	9	0.025641026	0.013452915	19	447	0.487179487	0.668161435	HT	10	Pinus_strobus	1785	Pinus	Pinaceae	Pinales	namerica	gymno	49.53117194	12.94255694	ecm	87	722	202078	4	380	1	0.166666667
HT05	26	215	6	121	0.230769231	0.562790698	10	42	0.384615385	0.195348837	HT	10	Pseudotsuga_menziesii	1830	Pseudotsuga	Pinaceae	Pinales	namerica	gymno	49.53172694	12.93968083	ecm	87	709	202078	4	380	1	0.166666667
HT06	36	1412	2	1004	0.055555556	0.711048159	16	150	0.444444444	0.106232295	HT	10	Pinus_strobus	1785	Pinus	Pinaceae	Pinales	namerica	gymno	49.53167694	12.93926583	ecm	87	709	202078	4	380	1	0.166666667
HT07	43	539	8	232	0.186046512	0.430426716	15	115	0.348837209	0.213358071	HT	10	Pseudotsuga_menziesii	1830	Pseudotsuga	Pinaceae	Pinales	namerica	gymno	49.533505	12.93703694	ecm	85	761	202078	4	380	1	0.166666667
CHO05	64	5790	0	0	0	0	45	5577	0.703125	0.963212435	CHO	11	Pseudotsuga_menziesii	1830	Pseudotsuga	Pinaceae	Pinales	namerica	gymno	50.51264389	13.44516389	ecm	81	620	66000	2	480	1	0.333333333
CHO06	57	1214	22	553	0.385964912	0.455518946	17	433	0.298245614	0.356672158	CHO	11	Pseudotsuga_menziesii	1830	Pseudotsuga	Pinaceae	Pinales	namerica	gymno	50.51321694	13.44348083	ecm	81	620	66000	2	480	1	0.333333333
CHO07	55	3074	13	2247	0.236363636	0.730969421	19	279	0.345454545	0.090761223	CHO	11	Castanea_sativa	1562	Castanea	Fagaceae	Fagales	europe	angio	50.51295778	13.44313194	ecm	81	620	66000	2	480	1	0.333333333
JE01	71	7657	10	6145	0.14084507	0.802533629	25	1119	0.352112676	0.146140786	JE	12	Cercidiphyllum_japonicum	1910	Cercidiphyllum	Cercidiphyllaceae	Saxifragales	asia	angio	49.74458583	14.78488389	am	82	613	122712	8	410	0.363636364	0.454545455
JE02	78	1903	19	1309	0.243589744	0.687861272	30	383	0.384615385	0.201261167	JE	12	Sophora_japonica	1835	Sophora	Fabaceae	Fabales	asia	angio	49.74440083	14.78478917	am	82	613	122712	8	410	0.363636364	0.454545455
JE04	58	10808	16	10133	0.275862069	0.937546262	15	138	0.25862069	0.01276832	JE	12	Pinus_wallichiana	1852	Pinus	Pinaceae	Pinales	asia	gymno	49.74284972	14.78705917	ecm	82	613	122712	8	410	0.363636364	0.454545455
JE05	26	323	0	0	0	0	12	134	0.461538462	0.414860681	JE	12	Sequoiadendron_giganteum	1859	Sequoiadendron	Cupressaceae	Pinales	namerica	gymno	49.74291306	14.78735444	am	82	613	122712	8	410	0.363636364	0.454545455
JE06	81	2738	5	81	0.061728395	0.029583638	38	2121	0.469135802	0.774653031	JE	12	Robinia_pseudoacacia	1710	Robinia	Fabaceae	Fabales	namerica	angio	49.7429976	14.78836778	am	82	613	122712	8	410	0.363636364	0.454545455
JE07	24	756	5	591	0.208333333	0.781746032	6	28	0.25	0.037037037	JE	12	Robinia_pseudoacacia	1710	Robinia	Fabaceae	Fabales	namerica	angio	49.7429975	14.78836778	am	82	613	122712	8	410	0.363636364	0.454545455
JE08	64	1634	7	623	0.109375	0.38127295	21	204	0.328125	0.124847001	JE	12	Robinia_pseudoacacia	1710	Robinia	Fabaceae	Fabales	namerica	angio	49.74263556	14.78818611	am	82	613	122712	8	410	0.363636364	0.454545455
JE10	13	440	0	0	0	0	5	396	0.384615385	0.9	JE	12	Metasequoia_glyptostroboides	1949	Metasequoia	Cupressaceae	Pinales	asia	gymno	49.74411361	14.78716167	am	82	613	122712	8	410	0.363636364	0.454545455
JE12	50	987	3	134	0.06	0.135764944	22	401	0.44	0.406281662	JE	12	Picea_pungens	1910	Picea	Pinaceae	Pinales	namerica	gymno	49.74491278	14.78566861	ecm	82	613	122712	8	410	0.363636364	0.454545455
JE13	73	2724	10	1071	0.136986301	0.393171806	36	1311	0.493150685	0.481277533	JE	12	Picea_pungens	1910	Picea	Pinaceae	Pinales	namerica	gymno	49.74505556	14.7857325	ecm	82	613	122712	8	410	0.363636364	0.454545455
JE14	22	1702	6	1567	0.272727273	0.920681551	5	51	0.227272727	0.029964747	JE	12	Pseudotsuga_menziesii	1830	Pseudotsuga	Pinaceae	Pinales	namerica	gymno	49.74505361	14.78613778	ecm	82	613	122712	8	410	0.363636364	0.454545455
KA01	21	171	0	0	0	0	14	144	0.666666667	0.842105263	KA	14	Platanus_×_acerifolia	1835	Platanus	Platanaceae	Proteales	hybrid	angio	49.98262389	15.33781639	am	94	574	252000	6	225	0.454545455	0.545454545
KA02	27	271	0	0	0	0	23	250	0.851851852	0.922509225	KA	14	Platanus_×_acerifolia	1835	Platanus	Platanaceae	Proteales	hybrid	angio	49.98242917	15.33796194	am	94	574	252000	6	225	0.454545455	0.545454545
KA03	40	641	2	9	0.05	0.014040562	20	400	0.5	0.624024961	KA	14	Platanus_×_acerifolia	1835	Platanus	Platanaceae	Proteales	hybrid	angio	49.98235972	15.33777056	am	94	574	252000	6	225	0.454545455	0.545454545
KA04	34	1051	1	97	0.029411765	0.092293054	14	774	0.411764706	0.736441484	KA	14	Pinus_cembra	1852	Pinus	Pinaceae	Pinales	europe	gymno	49.98042222	15.34752611	ecm	93	586	252000	6	225	0.454545455	0.545454545
KA05	39	418	0	0	0	0	15	186	0.384615385	0.444976077	KA	14	Pseudotsuga_menziesii	1830	Pseudotsuga	Pinaceae	Pinales	namerica	gymno	49.97977639	15.34829167	ecm	93	586	252000	6	225	0.454545455	0.545454545
KA06	46	823	4	91	0.086956522	0.110571081	23	552	0.5	0.670716889	KA	14	Tsuga_canadensis	1812	Tsuga	Pinaceae	Pinales	namerica	gymno	49.97984917	15.34844806	ecm	93	586	252000	6	225	0.454545455	0.545454545
KA07	39	482	2	13	0.051282051	0.026970954	21	329	0.538461538	0.682572614	KA	14	Tsuga_canadensis	1812	Tsuga	Pinaceae	Pinales	namerica	gymno	49.97986556	15.34856639	ecm	93	586	252000	6	225	0.454545455	0.545454545
KA08	44	1811	6	985	0.136363636	0.543898399	15	549	0.340909091	0.303147432	KA	14	Pinus_strobus	1785	Pinus	Pinaceae	Pinales	namerica	gymno	49.98000917	15.34860139	ecm	93	586	252000	6	225	0.454545455	0.545454545
KA09	33	446	0	0	0	0	15	158	0.454545455	0.35426009	KA	14	Robinia_pseudoacacia	1710	Robinia	Fabaceae	Fabales	namerica	angio	49.97978167	15.34882361	am	93	586	252000	6	225	0.454545455	0.545454545
KA10	37	1258	0	0	0	0	29	1167	0.783783784	0.927662957	KA	14	Robinia_pseudoacacia	1710	Robinia	Fabaceae	Fabales	namerica	angio	49.97944028	15.34769611	am	93	586	252000	6	225	0.454545455	0.545454545
KA11	28	507	0	0	0	0	17	372	0.607142857	0.733727811	KA	14	Robinia_pseudoacacia	1710	Robinia	Fabaceae	Fabales	namerica	angio	49.97949139	15.347515	am	93	586	252000	6	225	0.454545455	0.545454545
KD01	58	3333	0	0	0	0	36	3036	0.620689655	0.910891089	KD	15	Magnolia_x_soulangeana	1844	Magnolia	Magnoliaceae	Magnoliales	hybrid	angio	50.251	13.3696	am	88	550	433000	2	320	0	0.5
KD02	15	162	0	0	0	0	10	137	0.666666667	0.845679012	KD	15	Magnolia_x_soulangeana	1844	Magnolia	Magnoliaceae	Magnoliales	hybrid	angio	50.2521	13.3699	am	88	550	433000	2	320	0	0.5
KD03	41	1391	0	0	0	0	32	1279	0.780487805	0.919482387	KD	15	Thuja_occidentalis	1785	Thuja	Cupressaceae	Pinales	namerica	gymno	50.2517	13.3703	am	88	550	433000	2	320	0	0.5
KD04	20	216	0	0	0	0	15	190	0.75	0.87962963	KD	15	Thuja_occidentalis	1785	Thuja	Cupressaceae	Pinales	namerica	gymno	50.2515	13.3698	am	88	550	433000	2	320	0	0.5
KK01	28	884	0	0	0	0	20	844	0.714285714	0.954751131	KK	16	Ailanthus_altissima	1799	Ailanthus	Simaroubaceae	Sapindales	asia	angio	50.15915389	15.45042306	am	91	586	184399	6	240	0.714285714	0.571428571
KK02	28	537	12	410	0.428571429	0.763500931	12	110	0.428571429	0.204841713	KK	16	Quercus_rubra	1799	Quercus	Fagaceae	Fagales	namerica	angio	50.15950444	15.44958056	ecm	91	614	184399	6	240	0.714285714	0.571428571
KK08	64	1173	10	456	0.15625	0.388746803	29	466	0.453125	0.397271952	KK	16	Tsuga_canadensis	1812	Tsuga	Pinaceae	Pinales	namerica	gymno	50.15840833	15.451985	ecm	91	586	184399	6	240	0.714285714	0.571428571
KK09	41	837	0	0	0	0	28	744	0.682926829	0.888888889	KK	16	Magnolia_x_soulangeana	1844	Magnolia	Magnoliaceae	Magnoliales	hybrid	angio	50.15816611	15.45197694	am	91	584	184399	6	240	0.714285714	0.571428571
KK10	25	136	3	18	0.12	0.132352941	6	33	0.24	0.242647059	KK	16	Tsuga_canadensis	1812	Tsuga	Pinaceae	Pinales	namerica	gymno	50.15834472	15.45196722	ecm	91	586	184399	6	240	0.714285714	0.571428571
KK12	72	1815	3	10	0.041666667	0.005509642	37	582	0.513888889	0.320661157	KK	16	Castanea_sativa	1562	Castanea	Fagaceae	Fagales	europe	angio	50.1580825	15.45160722	ecm	91	584	184399	6	240	0.714285714	0.571428571
KK13	27	179	1	7	0.037037037	0.039106145	15	116	0.555555556	0.648044693	KK	16	Picea_pungens	1910	Picea	Pinaceae	Pinales	namerica	gymno	50.1580875	15.45177056	ecm	91	584	184399	6	240	0.714285714	0.571428571
KNO01	58	2237	6	389	0.103448276	0.173893608	34	1665	0.586206897	0.744300402	KNO	17	Ginkgo_biloba	1809	Ginkgo	Ginkgoaceae	Ginkgoales	asia	gymno	50.38491694	13.173265	am	85	607	97737	7	300	0.5	0.625
KNO03	49	1488	3	725	0.06122449	0.487231183	33	662	0.673469388	0.444892473	KNO	17	Quercus_palustris	1835	Quercus	Fagaceae	Fagales	namerica	angio	50.38422389	13.17254194	ecm	85	607	97737	7	300	0.5	0.625
KNO04	40	791	0	0	0	0	30	712	0.75	0.900126422	KNO	17	Picea_pungens	1910	Picea	Pinaceae	Pinales	namerica	gymno	50.38434278	13.17268278	ecm	85	607	97737	7	300	0.5	0.625
KNO06	60	1586	14	447	0.233333333	0.28184111	28	871	0.466666667	0.549180328	KNO	17	Quercus_rubra	1799	Quercus	Fagaceae	Fagales	namerica	angio	50.38348389	13.17163194	ecm	85	607	97737	7	300	0.5	0.625
KNO07	61	2298	5	112	0.081967213	0.048738033	38	2036	0.62295082	0.885987815	KNO	17	Magnolia_x_soulangeana	1844	Magnolia	Magnoliaceae	Magnoliales	hybrid	angio	50.38318889	13.17130889	am	86	627	97737	7	300	0.5	0.625
KNO09	63	903	10	163	0.158730159	0.180509413	30	582	0.476190476	0.644518272	KNO	17	Pterocarya_fraxinifolia	1844	Pterocarya	Juglandaceae	Fagales	asia	angio	50.38481389	13.173725	am	85	607	97737	7	300	0.5	0.625
KNO10	46	795	1	3	0.02173913	0.003773585	33	705	0.717391304	0.886792453	KNO	17	Paulownia_tomentosa	1844	Paulownia	Paulowniaceae	Lamiales	asia	angio	50.384295	13.17496278	am	86	613	97737	7	300	0.5	0.625
KNO12	52	2067	0	0	0	0	34	1786	0.653846154	0.864054185	KNO	17	Picea_pungens	1910	Picea	Pinaceae	Pinales	namerica	gymno	50.38440389	13.175035	ecm	86	613	97737	7	300	0.5	0.625
KO01	34	408	2	67	0.058823529	0.164215686	9	195	0.264705882	0.477941176	KO	18	Thuja_occidentalis	1785	Thuja	Cupressaceae	Pinales	namerica	gymno	49.77830889	14.65805	am	87	591	198053	10	375	0.538461538	0.230769231
KO02	64	2529	4	64	0.0625	0.025306445	34	2218	0.53125	0.877026493	KO	18	Chamaecyparis_nootkatensis	1863	Chamaecyparis	Cupressaceae	Pinales	namerica	gymno	49.77832139	14.65769083	am	87	591	198053	10	375	0.538461538	0.230769231
KO03	48	1348	11	712	0.229166667	0.528189911	15	188	0.3125	0.139465875	KO	18	Tsuga_canadensis	1812	Tsuga	Pinaceae	Pinales	namerica	gymno	49.77824528	14.657535	ecm	87	591	198053	10	375	0.538461538	0.230769231
KO04	37	877	10	633	0.27027027	0.721778791	12	176	0.324324324	0.200684151	KO	18	Pseudotsuga_menziesii	1830	Pseudotsuga	Pinaceae	Pinales	namerica	gymno	49.77835528	14.65745722	ecm	87	591	198053	10	375	0.538461538	0.230769231
KO06	67	2096	10	883	0.149253731	0.421278626	29	926	0.432835821	0.441793893	KO	18	Liriodendron_tulipifera	1785	Liriodendron	Altingiaceae	Saxifragales	namerica	angio	49.77732639	14.65940917	am	86	602	198053	10	375	0.538461538	0.230769231
KO07	26	243	8	70	0.307692308	0.288065844	8	113	0.307692308	0.465020576	KO	18	Abies_concolor	1910	Abies	Pinaceae	Pinales	namerica	gymno	49.77695222	14.65911861	ecm	86	602	198053	10	375	0.538461538	0.230769231
KO08	21	1061	11	892	0.523809524	0.840716305	6	77	0.285714286	0.072573044	KO	18	Catalpa_bignonioides	1785	Catalpa	Bignoniaceae	Lamiales	namerica	angio	49.77670139	14.65870806	am	86	602	198053	10	375	0.538461538	0.230769231
KO10	38	1525	13	1256	0.342105263	0.823606557	11	90	0.289473684	0.059016393	KO	18	Tsuga_canadensis	1812	Tsuga	Pinaceae	Pinales	namerica	gymno	49.77609389	14.65804444	ecm	87	591	198053	10	375	0.538461538	0.230769231
KO11	53	1995	6	573	0.113207547	0.287218045	25	1100	0.471698113	0.551378446	KO	18	Chamaecyparis_lawsoniana	1859	Chamaecyparis	Cupressaceae	Pinales	namerica	gymno	49.77601722	14.65793056	am	87	591	198053	10	375	0.538461538	0.230769231
KO12	48	662	3	14	0.0625	0.021148036	23	417	0.479166667	0.629909366	KO	18	Platanus_×_acerifolia	1835	Platanus	Platanaceae	Proteales	hybrid	angio	49.77691556	14.65769778	am	87	591	198053	10	375	0.538461538	0.230769231
KO13	33	768	0	0	0	0	22	671	0.666666667	0.873697917	KO	18	Picea_pungens	1910	Picea	Pinaceae	Pinales	namerica	gymno	49.77713389	14.65764667	ecm	87	591	198053	10	375	0.538461538	0.230769231
KO14	48	799	5	149	0.104166667	0.186483104	23	352	0.479166667	0.440550688	KO	18	Picea_pungens	1910	Picea	Pinaceae	Pinales	namerica	gymno	49.77783806	14.65751194	ecm	87	591	198053	10	375	0.538461538	0.230769231
KO15	52	7196	8	176	0.153846154	0.024458032	29	6908	0.557692308	0.959977765	KO	18	Picea_pungens	1910	Picea	Pinaceae	Pinales	namerica	gymno	49.77808194	14.65479889	ecm	87	591	198053	10	375	0.538461538	0.230769231

sample_ID	total_sps.sym	total_seq.sym	ecm_sps	ecm_seq	ecm_share_sps.sym	ecm_share.sym	patho_sps	patho_seq	patho_share_sps.sym	patho_share.sym	park	park_nr	pl_species	pl_species_age_cr	pl_genus	pl_family	pl_order	pl_origin_cont	plant_type	latitude	longitude	m_type	mat	map	area	alien.treeSR	altitude	ecm.s	angio.s
KP01	84	4163	5	134	0.05952381	0.032188326	50	3685	0.595238095	0.885178957	KP	19	Platanus_×_acerifolia	1835	Platanus	Platanaceae	Proteales	hybrid	angio	50.33049639	15.27119556	am	90	658	100131	16	225	0.363636364	0.318181818
KP02	61	2396	1	3	0.016393443	0.001252087	35	1991	0.573770492	0.83096828	KP	19	Platanus_×_acerifolia	1835	Platanus	Platanaceae	Proteales	hybrid	angio	50.33068472	15.27121556	am	90	658	100131	16	225	0.363636364	0.318181818
KP03	66	4827	0	0	0	0	45	3946	0.681818182	0.81748498	KP	19	Ginkgo_biloba	1809	Ginkgo	Ginkgoaceae	Ginkgoales	asia	gymno	50.33081333	15.27116583	am	90	658	100131	16	225	0.363636364	0.318181818
KP04	80	3805	20	2364	0.25	0.621287779	31	902	0.3875	0.237056505	KP	19	Castanea_sativa	1562	Castanea	Fagaceae	Fagales	europe	angio	50.33073833	15.27142778	ecm	90	658	100131	16	225	0.363636364	0.318181818
KP05	82	1926	0	0	0	0	44	1008	0.536585366	0.523364486	KP	19	Thuja_occidentalis	1785	Thuja	Cupressaceae	Pinales	namerica	gymno	50.33098389	15.27168972	am	90	658	100131	16	225	0.363636364	0.318181818
KP06	86	4634	2	536	0.023255814	0.115666811	53	3064	0.61627907	0.661199827	KP	19	Thuja_occidentalis	1785	Thuja	Cupressaceae	Pinales	namerica	gymno	50.33108333	15.27171944	am	90	658	100131	16	225	0.363636364	0.318181818
KP07	42	1620	9	803	0.214285714	0.495679012	22	726	0.523809524	0.448148148	KP	19	Taxodium_distichum	1835	Taxodium	Cupressaceae	Pinales	namerica	gymno	50.33134972	15.27225111	am	90	658	100131	16	225	0.363636364	0.318181818
KP09	60	2223	2	58	0.033333333	0.026090868	35	877	0.583333333	0.394511921	KP	19	Abies_concolor	1910	Abies	Pinaceae	Pinales	namerica	gymno	50.3308075	15.2726525	ecm	90	658	100131	16	225	0.363636364	0.318181818
KP10	41	2539	0	0	0	0	27	2268	0.658536585	0.893265065	KP	19	Chamaecyparis_nootkatensis	1863	Chamaecyparis	Cupressaceae	Pinales	namerica	gymno	50.33079361	15.27247111	am	90	658	100131	16	225	0.363636364	0.318181818
KP11	29	539	1	3	0.034482759	0.005565863	21	499	0.724137931	0.925788497	KP	19	Chamaecyparis_pisifera	1865	Chamaecyparis	Cupressaceae	Pinales	asia	gymno	50.33052111	15.27243889	am	90	658	100131	16	225	0.363636364	0.318181818
KP12	61	2408	0	0	0	0	37	1686	0.606557377	0.700166113	KP	19	Chamaecyparis_pisifera	1865	Chamaecyparis	Cupressaceae	Pinales	asia	gymno	50.33045111	15.27239833	am	90	658	100131	16	225	0.363636364	0.318181818
KP13	60	3661	0	0	0	0	36	2187	0.6	0.597377766	KP	19	Picea_pungens	1910	Picea	Pinaceae	Pinales	namerica	gymno	50.33046222	15.272585	ecm	90	658	100131	16	225	0.363636364	0.318181818
KP15	60	1738	0	0	0	0	34	1390	0.566666667	0.79976985	KP	19	Picea_pungens	1910	Picea	Pinaceae	Pinales	namerica	gymno	50.33033583	15.27303722	ecm	90	658	100131	16	225	0.363636364	0.318181818
KP16	57	1535	1	6	0.01754386	0.003908795	37	1345	0.649122807	0.876221498	KP	19	Metasequoia_glyptostroboides	1949	Metasequoia	Cupressaceae	Pinales	asia	gymno	50.33075528	15.2745325	am	90	658	100131	16	225	0.363636364	0.318181818
KP17	66	11748	13	10513	0.196969697	0.894875724	29	440	0.439393939	0.037453184	KP	19	Tsuga_canadensis	1812	Tsuga	Pinaceae	Pinales	namerica	gymno	50.33093056	15.27551722	ecm	89	672	100131	16	225	0.363636364	0.318181818
KP18	44	2387	15	1582	0.340909091	0.662756598	19	713	0.431818182	0.298701299	KP	19	Pinus_strobus	1785	Pinus	Pinaceae	Pinales	namerica	gymno	50.3308875	15.27534333	ecm	89	672	100131	16	225	0.363636364	0.318181818
KP19	60	1939	0	0	0	0	40	1546	0.666666667	0.797318205	KP	19	Pseudotsuga_menziesii	1830	Pseudotsuga	Pinaceae	Pinales	namerica	gymno	50.33029528	15.272145	ecm	90	658	100131	16	225	0.363636364	0.318181818
KP20	66	2061	8	302	0.121212121	0.14653081	33	1460	0.5	0.708393984	KP	19	Pseudotsuga_menziesii	1830	Pseudotsuga	Pinaceae	Pinales	namerica	gymno	50.33031583	15.27196833	ecm	90	658	100131	16	225	0.363636364	0.318181818
KP21	77	2570	0	0	0	0	40	2127	0.519480519	0.827626459	KP	19	Liriodendron_tulipifera	1785	Liriodendron	Altingiaceae	Saxifragales	namerica	angio	50.33009806	15.27164194	am	90	658	100131	16	225	0.363636364	0.318181818
KP22	83	1972	1	6	0.012048193	0.003042596	44	1152	0.530120482	0.584178499	KP	19	Magnolia_stellata	1927	Magnolia	Magnoliaceae	Magnoliales	asia	angio	50.33011	15.27151278	am	90	658	100131	16	225	0.363636364	0.318181818
KP23	46	1051	1	3	0.02173913	0.002854424	33	989	0.717391304	0.941008563	KP	19	Magnolia_stellata	1927	Magnolia	Magnoliaceae	Magnoliales	asia	angio	50.33023806	15.27155444	am	90	658	100131	16	225	0.363636364	0.318181818
KP24	79	4476	0	0	0	0	41	3492	0.518987342	0.780160858	KP	19	Catalpa_bignonioides	1785	Catalpa	Bignoniaceae	Lamiales	namerica	angio	50.33026389	15.27141528	am	90	658	100131	16	225	0.363636364	0.318181818
KROM01	52	1322	1	47	0.019230769	0.035552194	31	1098	0.596153846	0.830559758	KROM	20	Catalpa_bignonioides	1785	Catalpa	Bignoniaceae	Lamiales	namerica	angio	49.30175583	17.394925	am	98	562	436000	15	190	0.266666667	0.666666667
KROM02	25	317	5	29	0.2	0.09148265	12	181	0.48	0.570977918	KROM	20	Pinus_strobus	1785	Pinus	Pinaceae	Pinales	namerica	gymno	49.30164278	17.39567389	ecm	98	562	436000	15	190	0.266666667	0.666666667
KROM03	46	656	0	0	0	0	29	543	0.630434783	0.827743902	KROM	20	Platanus_×_acerifolia	1835	Platanus	Platanaceae	Proteales	hybrid	angio	49.30182083	17.39551194	am	98	562	436000	15	190	0.266666667	0.666666667
KROM04	45	1148	1	88	0.022222222	0.076655052	28	862	0.622222222	0.75087108	KROM	20	Liriodendron_tulipifera	1785	Liriodendron	Altingiaceae	Saxifragales	namerica	angio	49.30194778	17.39562278	am	98	562	436000	15	190	0.266666667	0.666666667
KROM05	19	659	0	0	0	0	14	605	0.736842105	0.918057663	KROM	20	Ginkgo_biloba	1809	Ginkgo	Ginkgoaceae	Ginkgoales	asia	gymno	49.30233583	17.39543694	am	98	562	436000	15	190	0.266666667	0.666666667
KROM06	58	2642	0	0	0	0	41	2487	0.706896552	0.941332324	KROM	20	Taxodium_ascensens	1859	Taxodium	Cupressaceae	Pinales	namerica	gymno	49.30242194	17.39561	am	98	562	436000	15	190	0.266666667	0.666666667
KROM07	37	2444	0	0	0	0	29	2397	0.783783784	0.980769231	KROM	20	Pinus_ponderosa	1845	Pinus	Pinaceae	Pinales	namerica	gymno	49.304205	17.39580889	ecm	98	562	436000	15	190	0.266666667	0.666666667
KROM08	53	1213	0	0	0	0	36	1128	0.679245283	0.929925804	KROM	20	Pseudotsuga_menziesii	1830	Pseudotsuga	Pinaceae	Pinales	namerica	gymno	49.30513889	17.39272389	ecm	98	562	436000	15	190	0.266666667	0.666666667
KROM09	47	1051	0	0	0	0	38	999	0.808510638	0.950523311	KROM	20	Gleditsia_triacanthos	1785	Gleditsia	Fabaceae	Fabales	namerica	angio	49.30451194	17.39245778	am	98	562	436000	15	190	0.266666667	0.666666667
KROM10	56	1319	1	3	0.017857143	0.00227445	40	1176	0.714285714	0.891584534	KROM	20	Juglans_cinerea	1835	Juglans	Juglandaceae	Fagales	namerica	angio	49.30626	17.39187083	am	98	562	436000	15	190	0.266666667	0.666666667
KROM11	40	925	0	0	0	0	25	738	0.625	0.797837838	KROM	20	Pterocarya_fraxinifolia	1844	Pterocarya	Juglandaceae	Fagales	asia	angio	49.307105	17.39005389	am	98	560	436000	15	190	0.266666667	0.666666667
KROM12	52	2099	1	7	0.019230769	0.003334921	37	1896	0.711538462	0.90328728	KROM	20	Pterocarya_rhoifolia	1910	Pterocarya	Juglandaceae	Fagales	asia	angio	49.30720278	17.39001278	am	98	560	436000	15	190	0.266666667	0.666666667
KROM13	44	2867	1	3	0.022727273	0.00104639	34	2793	0.772727273	0.974189048	KROM	20	Sophora_japonica	1835	Sophora	Fabaceae	Fabales	asia	angio	49.30697944	17.38770306	am	98	560	436000	15	190	0.266666667	0.666666667
KROM14	53	579	4	78	0.075471698	0.134715026	28	291	0.528301887	0.502590674	KROM	20	Castanea_sativa	1562	Castanea	Fagaceae	Fagales	europe	angio	49.30707	17.388765	ecm	98	560	436000	15	190	0.266666667	0.666666667
KROM16	49	1117	2	34	0.040816327	0.030438675	31	795	0.632653061	0.711727842	KROM	20	Gymnocladus_dioicus	1844	Gymnocladus	Fabaceae	Fabales	namerica	angio	49.30422694	17.38967	am	98	560	436000	15	190	0.266666667	0.666666667
KYN01	33	978	3	97	0.090909091	0.099182004	18	708	0.545454545	0.72392638	KYN	13	Picea_pungens	1910	Picea	Pinaceae	Pinales	namerica	gymno	50.00485417	12.60687278	ecm	71	720	54372	7	600	0.785714286	0.071428571
KYN02	37	826	7	601	0.189189189	0.727602906	15	146	0.405405405	0.176755448	KYN	13	Chamaecyparis_nootkatensis	1863	Chamaecyparis	Cupressaceae	Pinales	namerica	gymno	50.00498778	12.60721417	am	71	720	54372	7	600	0.785714286	0.071428571
KYN03	21	102	6	29	0.285714286	0.284313725	7	34	0.333333333	0.333333333	KYN	13	Acer_rubrum	1835	Acer	Sapindaceae	Sapindales	namerica	angio	50.0045253	12.6068097	am	71	720	54372	7	600	0.785714286	0.071428571
KYN05	52	917	1	40	0.019230769	0.043620502	25	529	0.480769231	0.576881134	KYN	13	Pinus_strobus	1785	Pinus	Pinaceae	Pinales	namerica	gymno	50.00408167	12.6068	ecm	71	720	54372	7	600	0.785714286	0.071428571
KYN06	39	1580	17	1039	0.435897436	0.657594937	9	101	0.230769231	0.063924051	KYN	13	Tsuga_canadensis	1812	Tsuga	Pinaceae	Pinales	namerica	gymno	50.0036525	12.60528222	ecm	71	720	54372	7	600	0.785714286	0.071428571
KYN07	64	1714	12	269	0.1875	0.156942824	29	1128	0.453125	0.658109685	KYN	13	Picea_pungens	1910	Picea	Pinaceae	Pinales	namerica	gymno	50.00356056	12.60613806	ecm	71	720	54372	7	600	0.785714286	0.071428571
KYN09	42	497	1	3	0.023809524	0.006036217	20	300	0.476190476	0.60362173	KYN	13	Pseudotsuga_menziesii	1830	Pseudotsuga	Pinaceae	Pinales	namerica	gymno	50.00334222	12.60533083	ecm	71	720	54372	7	600	0.785714286	0.071428571
KYN10	56	1828	3	545	0.053571429	0.298140044	31	1080	0.553571429	0.590809628	KYN	13	Picea_pungens	1910	Picea	Pinaceae	Pinales	namerica	gymno	50.003485	12.60533861	ecm	71	720	54372	7	600	0.785714286	0.071428571
KYN11	17	302	3	106	0.176470588	0.350993377	9	175	0.529411765	0.579470199	KYN	13	Pinus_strobus	1785	Pinus	Pinaceae	Pinales	namerica	gymno	50.00377389	12.6051175	ecm	71	720	54372	7	600	0.785714286	0.071428571
KYN12	44	1308	13	419	0.295454545	0.320336391	17	775	0.386363636	0.592507645	KYN	13	Pinus_strobus	1785	Pinus	Pinaceae	Pinales	namerica	gymno	50.00365583	12.60478194	ecm	71	720	54372	7	600	0.785714286	0.071428571
KYN13	24	1296	8	1072	0.333333333	0.827160494	6	87	0.25	0.06712963	KYN	13	Pseudotsuga_menziesii	1830	Pseudotsuga	Pinaceae	Pinales	namerica	gymno	50.00369278	12.6048825	ecm	71	720	54372	7	600	0.785714286	0.071428571
KYN14	46	625	4	24	0.086956522	0.0384	26	433	0.565217391	0.6928	KYN	13	Picea_pungens	1910	Picea	Pinaceae	Pinales	namerica	gymno	50.00467694	12.60372417	ecm	71	720	54372	7	600	0.785714286	0.071428571
KYN15	56	3998	2	10	0.035714286	0.002501251	37	3345	0.660714286	0.836668334	KYN	13	Chamaecyparis_lawsoniana	1859	Chamaecyparis	Cupressaceae	Pinales	namerica	gymno	50.00487556	12.60387306	am	71	720	54372	7	600	0.785714286	0.071428571
KYN16	23	363	2	9	0.086956522	0.024793388	16	285	0.695652174	0.785123967	KYN	13	Pinus_strobus	1785	Pinus	Pinaceae	Pinales	namerica	gymno	50.00480278	12.60395389	ecm	71	720	54372	7	600	0.785714286	0.071428571
KZ02	26	1250	12	1185	0.461538462	0.948	6	32	0.230769231	0.0256	KZ	21	Abies_pinsapo	1845	Abies	Pinaceae	Pinales	europe	gymno	49.66950472	13.53006833	ecm	86	567	140732	11	390	0.636363636	0.272727273
KZ03	15	123	0	0	0	0	9	100	0.6	0.81300813	KZ	21	Phellodendron_amurense	1911	Phellodendron	Rutaceae	Sapindales	asia	angio	49.66948194	13.52918167	am	86	567	140732	11	390	0.636363636	0.272727273
KZ04	29	393	4	133	0.137931034	0.338422392	17	221	0.586206897	0.562340967	KZ	21	Castanea_sativa	1562	Castanea	Fagaceae	Fagales	europe	angio	49.669585	13.52887917	ecm	86	567	140732	11	390	0.636363636	0.272727273
KZ05	66	7736	15	7189	0.227272727	0.929291624	26	420	0.393939394	0.054291624	KZ	21	Picea_orientalis	1813	Picea	Pinaceae	Pinales	asia	gymno	49.66929194	13.52937528	ecm	86	567	140732	11	390	0.636363636	0.272727273
KZ06	42	435	1	3	0.023809524	0.006896552	23	326	0.547619048	0.749425287	KZ	21	Chamaecyparis_lawsoniana	1859	Chamaecyparis	Cupressaceae	Pinales	namerica	gymno	49.66902167	13.53031111	am	86	567	140732	11	390	0.636363636	0.272727273
KZ07	26	1282	12	1133	0.461538462	0.883775351	5	57	0.192307692	0.044461778	KZ	21	Abies_grandis	1879	Abies	Pinaceae	Pinales	namerica	gymno	49.66864083	13.53441611	ecm	84	608	140732	11	390	0.636363636	0.272727273
KZ08	18	2191	15	2178	0.833333333	0.994066636	0	0	0	0	KZ	21	Pseudotsuga_menziesii	1830	Pseudotsuga	Pinaceae	Pinales	namerica	gymno	49.66862139	13.53506917	ecm	84	608	140732	11	390	0.636363636	0.272727273
KZ09	31	268	4	19	0.129032258	0.070895522	15	197	0.483870968	0.735074627	KZ	21	Pinus_strobus	1785	Pinus	Pinaceae	Pinales	namerica	gymno	49.66915167	13.53218444	ecm	86	567	140732	11	390	0.636363636	0.272727273
KZ10	26	318	0	0	0	0	18	284	0.692307692	0.893081761	KZ	21	Thuja_plicata	1844	Thuja	Cupressaceae	Pinales	namerica	gymno	49.67083778	13.53083778	am	86	567	140732	11	390	0.636363636	0.272727273
KZ11	11	110	0	0	0	0	6	58	0.545454545	0.527272727	KZ	21	Liriodendron_tulipifera	1785	Liriodendron	Altingiaceae	Saxifragales	namerica	angio	49.67109278	13.51905361	am	87	523	140732	11	390	0.636363636	0.272727273
KZ12	42	1947	18	1718	0.428571429	0.882383154	11	51	0.261904762	0.026194145	KZ	21	Tsuga_canadensis	1812	Tsuga	Pinaceae	Pinales	namerica	gymno	49.67160389	13.52915889	ecm	86	567	140732	11	390	0.636363636	0.272727273
LDN01	41	714	0	0	0	0	29	623	0.707317073	0.87254902	LED	23	Thuja_plicata	1844	Thuja	Cupressaceae	Pinales	namerica	gymno	48.80124222	16.80709333	am	103	482	1570000	13	170	0.307692308	0.538461538
LDN02	40	3200	0	0	0	0	23	2902	0.575	0.906875	LED	23	Pterocarya_fraxinifolia	1844	Pterocarya	Juglandaceae	Fagales	asia	angio	48.80141333	16.80776722	am	103	482	1570000	13	170	0.307692308	0.538461538
LDN03	47	1519	1	5	0.021276596	0.003291639	34	1420	0.723404255	0.934825543	LED	23	Abies_concolor	1910	Abies	Pinaceae	Pinales	namerica	gymno	48.8016725	16.80783833	ecm	103	482	1570000	13	170	0.307692308	0.538461538
LDN04	51	1874	2	180	0.039215686	0.096051227	39	1638	0.764705882	0.874066169	LED	23	Pinus_strobus	1785	Pinus	Pinaceae	Pinales	namerica	gymno	48.80147944	16.80761917	ecm	103	482	1570000	13	170	0.307692308	0.538461538
LDN05	47	1631	1	50	0.021276596	0.030656039	35	1436	0.744680851	0.880441447	LED	23	Pseudotsuga_menziesii	1830	Pseudotsuga	Pinaceae	Pinales	namerica	gymno	48.80204639	16.80689167	ecm	103	482	1570000	13	170	0.307692308	0.538461538
LDN06	52	907	3	37	0.057692308	0.040793826	34	655	0.653846154	0.72216097	LED	23	Platanus_×_acerifolia	1835	Platanus	Platanaceae	Proteales	hybrid	angio	48.80237972	16.80705556	am	103	482	1570000	13	170	0.307692308	0.538461538
LDN07	43	1447	0	0	0	0	34	1325	0.790697674	0.91568763	LED	23	Pinus_ponderosa	1845	Pinus	Pinaceae	Pinales	namerica	gymno	48.80321833	16.80623889	ecm	103	482	1570000	13	170	0.307692308	0.538461538
LDN08	43	2417	2	7	0.046511628	0.002896152	33	2363	0.76744186	0.977658254	LED	23	Gymnocladus_dioicus	1844	Gymnocladus	Fabaceae	Fabales	namerica	angio	48.80298361	16.80838889	am	103	483	1570000	13	170	0.307692308	0.538461538
LDN09	47	796	6	143	0.127659574	0.179648241	25	553	0.531914894	0.694723618	LED	23	Catalpa_bignonioides	1785	Catalpa	Bignoniaceae	Lamiales	namerica	angio	48.80288028	16.809425	am	103	483	1570000	13	170	0.307692308	0.538461538
LDN10	41	897	3	16	0.073170732	0.017837235	28	821	0.682926829	0.915273133	LED	23	Ginkgo_biloba	1809	Ginkgo	Ginkgoaceae	Ginkgoales	asia	gymno	48.80271306	16.80826278	am	103	483	1570000	13	170	0.307692308	0.538461538
LDN11	33	1472	0	0	0	0	23	1386	0.696969697	0.941576087	LED	23	Sophora_japonica	1835	Sophora	Fabaceae	Fabales	asia	angio	48.80270972	16.808215	am	103	483	1570000	13	170	0.307692308	0.538461538
LDN12	47	1582	1	3	0.021276596	0.001896334	31	1482	0.659574468	0.936788875	LED	23	Cercidiphyllum_japonicum	1910	Cercidiphyllum	Cercidiphyllaceae	Saxifragales	asia	angio	48.80270722	16.80817694	am	103	482	1570000	13	170	0.307692308	0.538461538
LDN13	53	1549	2	8	0.037735849	0.005164622	38	1318	0.716981132	0.85087153	LED	23	Liriodendron_tulipifera	1785	Liriodendron	Altingiaceae	Saxifragales	namerica	angio	48.80210778	16.81003556	am	103	483	1570000	13	170	0.307692308	0.538461538
LI01	60	1434	0	0	0	0	32	1053	0.533333333	0.734309623	LI	24	Celtis_occidentalis	1835	Celtis	Cannabaceae	Rosales	namerica	angio	50.40539722	14.04474917	am	94	481	276000	5	165	0.090909091	0.909090909
LI06	53	2247	0	0	0	0	39	2061	0.735849057	0.917222964	LI	24	Celtis_occidentalis	1835	Celtis	Cannabaceae	Rosales	namerica	angio	50.40534333	14.047355	am	94	481	276000	5	165	0.090909091	0.909090909
LI07	37	1404	0	0	0	0	22	1322	0.594594595	0.941595442	LI	24	Picea_pungens	1910	Picea	Pinaceae	Pinales	namerica	gymno	50.40508139	14.04745472	ecm	94	481	276000	5	165	0.090909091	0.909090909
LI09	32	1267	1	3	0.03125	0.002367798	25	1198	0.78125	0.945540647	LI	24	Celtis_occidentalis	1835	Celtis	Cannabaceae	Rosales	namerica	angio	50.40516639	14.04781222	am	94	481	276000	5	165	0.090909091	0.909090909
LI10	38	2196	0	0	0	0	32	2092	0.842105263	0.952641166	LI	24	Celtis_occidentalis	1835	Celtis	Cannabaceae	Rosales	namerica	angio	50.40514722	14.04788667	am	94	481	276000	5	165	0.090909091	0.909090909

sample_ID	total_sps.sym	total_seq.sym	ecm_sps	ecm_seq	ecm_share_sps.sym	ecm_share.sym	patho_sps	patho_seq	patho_share_sps.sym	patho_share.sym	park	park_nr	pl_species	pl_species_age_cr	pl_genus	pl_family	pl_order	pl_origin_cont	plant_type	latitude	longitude	m_type	mat	map	area	alien.treeSR	altitude	ecm.s	angio.s
LI11	47	1693	0	0	0	0	31	1558	0.659574468	0.920259894	LI	24	Catalpa_bignonioides	1785	Catalpa	Bignoniaceae	Lamiales	namerica	angio	50.40496361	14.0483425	am	94	481	276000	5	165	0.090909091	0.909090909
LI12	46	606	0	0	0	0	30	530	0.652173913	0.874587459	LI	24	Catalpa_bignonioides	1785	Catalpa	Bignoniaceae	Lamiales	namerica	angio	50.40502861	14.04834806	am	94	481	276000	5	165	0.090909091	0.909090909
LI14	66	2369	22	1920	0.333333333	0.810468552	22	264	0.333333333	0.111439426	LI	24	Platanus_×_acerifolia	1835	Platanus	Platanaceae	Proteales	hybrid	angio	50.40370861	14.04842639	am	94	481	276000	5	165	0.090909091	0.909090909
LI15	42	685	16	499	0.380952381	0.728467153	14	131	0.333333333	0.191240876	LI	24	Platanus_×_acerifolia	1835	Platanus	Platanaceae	Proteales	hybrid	angio	50.40373944	14.04833806	am	94	481	276000	5	165	0.090909091	0.909090909
LI16	47	862	26	645	0.553191489	0.748259861	10	103	0.212765957	0.119489559	LI	24	Robinia_pseudoacacia	1710	Robinia	Fabaceae	Fabales	namerica	angio	50.40383278	14.04795389	am	94	481	276000	5	165	0.090909091	0.909090909
LI17	28	704	0	0	0	0	20	665	0.714285714	0.944602273	LI	24	Robinia_pseudoacacia	1710	Robinia	Fabaceae	Fabales	namerica	angio	50.40479417	14.04543806	am	94	481	276000	5	165	0.090909091	0.909090909
LIB01	44	1961	0	0	0	0	33	1888	0.75	0.962774095	LB	22	Platanus_×_acerifolia	1835	Platanus	Platanaceae	Proteales	hybrid	angio	50.40864	14.44468917	am	93	569	113189	4	170	0.428571429	0.285714286
LIB02	64	2290	14	1356	0.21875	0.592139738	32	824	0.5	0.359825328	LB	22	Pinus_strobus	1785	Pinus	Pinaceae	Pinales	namerica	gymno	50.4068575	14.44406694	ecm	94	537	113189	4	170	0.428571429	0.285714286
LIB03	63	2096	19	1092	0.301587302	0.520992366	27	891	0.428571429	0.42509542	LB	22	Pinus_strobus	1785	Pinus	Pinaceae	Pinales	namerica	gymno	50.40694417	14.44422083	ecm	94	537	113189	4	170	0.428571429	0.285714286
LIB04	53	847	5	103	0.094339623	0.121605667	28	518	0.528301887	0.611570248	LB	22	Pinus_strobus	1785	Pinus	Pinaceae	Pinales	namerica	gymno	50.40688306	14.44431917	ecm	94	537	113189	4	170	0.428571429	0.285714286
LIB05	55	699	0	0	0	0	31	519	0.563636364	0.74248927	LB	22	Juglans_cinerea	1835	Juglans	Juglandaceae	Fagales	namerica	angio	50.40678167	14.44435833	am	94	537	113189	4	170	0.428571429	0.285714286
LIB06	46	1205	2	9	0.043478261	0.00746888	30	1126	0.652173913	0.934439834	LB	22	Thuja_occidentalis	1785	Thuja	Cupressaceae	Pinales	namerica	gymno	50.40841333	14.44636056	am	93	569	113189	4	170	0.428571429	0.285714286
LIB07	45	992	2	59	0.044444444	0.059475806	27	640	0.6	0.64516129	LB	22	Thuja_occidentalis	1785	Thuja	Cupressaceae	Pinales	namerica	gymno	50.40848889	14.44648056	am	93	569	113189	4	170	0.428571429	0.285714286
LL01	60	1787	1	4	0.016666667	0.002238388	41	1432	0.683333333	0.801343033	LL	25	Aesculus_hippocastanum	1576	Aesculus	Sapindaceae	Sapindales	europe	angio	50.20309028	14.83320722	am	93	576	203851	3	210	0	1
LL02	33	2972	0	0	0	0	26	2385	0.787878788	0.802489906	LL	25	Platanus_×_acerifolia	1835	Platanus	Platanaceae	Proteales	hybrid	angio	50.20271167	14.83318972	am	94	570	203851	3	210	0	1
LL03	71	2743	3	11	0.042253521	0.004010208	31	2029	0.436619718	0.739701057	LL	25	Aesculus_hippocastanum	1576	Aesculus	Sapindaceae	Sapindales	europe	angio	50.20343917	14.83297333	am	94	570	203851	3	210	0	1
LL04	17	153	2	73	0.117647059	0.477124183	10	60	0.588235294	0.392156863	LL	25	Catalpa_bignonioides	1785	Catalpa	Bignoniaceae	Lamiales	namerica	angio	50.20305	14.83866417	am	93	576	203851	3	210	0	1
LL05	53	801	0	0	0	0	33	704	0.622641509	0.878901373	LL	25	Catalpa_bignonioides	1785	Catalpa	Bignoniaceae	Lamiales	namerica	angio	50.20323778	14.83867389	am	93	576	203851	3	210	0	1
LO03	43	1719	4	606	0.093023256	0.352530541	15	579	0.348837209	0.336823735	LO	26	Chamaecyparis_lawsoniana	1859	Chamaecyparis	Cupressaceae	Pinales	namerica	gymno	50.287685	15.02361472	am	89	634	161622	10	240	0.181818182	0.727272727
LO04	42	2038	8	798	0.19047619	0.391560353	17	1113	0.404761905	0.546123651	LO	26	Sophora_japonica	1835	Sophora	Fabaceae	Fabales	asia	angio	50.2877025	15.0238825	am	89	634	161622	10	240	0.181818182	0.727272727
LO06	67	1819	15	342	0.223880597	0.188015393	34	1150	0.507462687	0.632215503	LO	26	Sophora_japonica	1835	Sophora	Fabaceae	Fabales	asia	angio	50.28777472	15.02439556	am	89	634	161622	10	240	0.181818182	0.727272727
LO07	40	311	0	0	0	0	13	155	0.325	0.498392283	LO	26	Acer_palmatum	1835	Acer	Sapindaceae	Sapindales	asia	angio	50.28739583	15.0245075	am	89	634	161622	10	240	0.181818182	0.727272727
LO08	48	2199	0	0	0	0	33	1826	0.6875	0.830377444	LO	26	Catalpa_bignonioides	1785	Catalpa	Bignoniaceae	Lamiales	namerica	angio	50.28740167	15.02489361	am	91	603	161622	10	240	0.181818182	0.727272727
LO09	51	1672	1	7	0.019607843	0.004186603	30	1453	0.588235294	0.869019139	LO	26	Magnolia_x_soulangeana	1844	Magnolia	Magnoliaceae	Magnoliales	hybrid	angio	50.28689028	15.02499583	am	91	603	161622	10	240	0.181818182	0.727272727
LO10	77	1919	0	0	0	0	35	1067	0.454545455	0.55601876	LO	26	Liriodendron_tulipifera	1785	Liriodendron	Altingiaceae	Saxifragales	namerica	angio	50.28668111	15.02513	am	91	603	161622	10	240	0.181818182	0.727272727
LO11	36	1991	9	1667	0.25	0.837267705	14	190	0.388888889	0.095429432	LO	26	Castanea_sativa	1562	Castanea	Fagaceae	Fagales	europe	angio	50.28673306	15.02635056	ecm	91	603	161622	10	240	0.181818182	0.727272727
LO12	45	1331	4	815	0.088888889	0.612321563	23	309	0.511111111	0.232156273	LO	26	Taxodium_distichum	1835	Taxodium	Cupressaceae	Pinales	namerica	gymno	50.28719111	15.0270425	am	91	603	161622	10	240	0.181818182	0.727272727
LO13	54	2215	1	3	0.018518519	0.001354402	35	2028	0.648148148	0.915575621	LO	26	Robinia_pseudoacacia	1710	Robinia	Fabaceae	Fabales	namerica	angio	50.2881975	15.02705056	am	91	603	161622	10	240	0.181818182	0.727272727
LO14	62	2270	10	633	0.161290323	0.278854626	35	942	0.564516129	0.414977974	LO	26	Pseudotsuga_menziesii	1830	Pseudotsuga	Pinaceae	Pinales	namerica	gymno	50.28835417	15.02713833	ecm	91	603	161622	10	240	0.181818182	0.727272727
ME01	51	1245	16	965	0.31372549	0.775100402	20	204	0.392156863	0.163855422	ME	27	Tsuga_canadensis	1812	Tsuga	Pinaceae	Pinales	namerica	gymno	50.1946375	14.52292	ecm	93	489	169124	9	200	0.4	0.3
ME02	54	597	7	143	0.12962963	0.239530988	25	269	0.462962963	0.450586265	ME	27	Chamaecyparis_nootkatensis	1863	Chamaecyparis	Cupressaceae	Pinales	namerica	gymno	50.19455167	14.52287556	am	93	489	169124	9	200	0.4	0.3
ME03	40	853	6	556	0.15	0.651817116	18	133	0.45	0.155920281	ME	27	Abies_concolor	1910	Abies	Pinaceae	Pinales	namerica	gymno	50.19433889	14.52244417	ecm	93	489	169124	9	200	0.4	0.3
ME04	58	717	3	16	0.051724138	0.022315202	35	451	0.603448276	0.629009763	ME	27	Cedrus_libani	1812	Cedrus	Pinaceae	Pinales	asia	gymno	50.1934	14.52091444	ecm	93	489	169124	9	200	0.4	0.3
ME05	54	2007	8	1360	0.148148148	0.677628301	21	429	0.388888889	0.213751868	ME	27	Thuja_occidentalis	1785	Thuja	Cupressaceae	Pinales	namerica	gymno	50.1956625	14.52164111	am	93	489	169124	9	200	0.4	0.3
ME06	46	1302	3	12	0.065217391	0.00921659	26	590	0.565217391	0.453149002	ME	27	Thuja_occidentalis	1785	Thuja	Cupressaceae	Pinales	namerica	gymno	50.1957	14.522	am	93	489	169124	9	200	0.4	0.3
ME07	71	2129	2	8	0.028169014	0.003757633	32	1721	0.450704225	0.808360733	ME	27	Liriodendron_tulipifera	1785	Liriodendron	Altingiaceae	Saxifragales	namerica	angio	50.19580722	14.52145917	am	93	489	169124	9	200	0.4	0.3
ME08	27	224	5	77	0.185185185	0.34375	14	107	0.518518519	0.477678571	ME	27	Gleditsia_triacanthos	1785	Gleditsia	Fabaceae	Fabales	namerica	angio	50.19571833	14.52091	am	93	489	169124	9	200	0.4	0.3
ME09	64	579	6	20	0.09375	0.034542314	26	252	0.40625	0.435233161	ME	27	Platanus_×_acerifolia	1835	Platanus	Platanaceae	Proteales	hybrid	angio	50.19579	14.52112889	am	93	489	169124	9	200	0.4	0.3
ME10	34	375	2	65	0.058823529	0.173333333	17	219	0.5	0.584	ME	27	Picea_pungens	1910	Picea	Pinaceae	Pinales	namerica	gymno	50.1954775	14.52488417	ecm	93	489	169124	9	200	0.4	0.3
NH01	53	670	0	0	0	0	30	410	0.566037736	0.611940299	NH	28	Chamaecyparis_nootkatensis	1863	Chamaecyparis	Cupressaceae	Pinales	namerica	gymno	48.79122083	14.78307139	am	83	705	27020	5	510	0.333333333	0.166666667
NH02	39	779	1	5	0.025641026	0.006418485	18	453	0.461538462	0.581514763	NH	28	Chamaecyparis_nootkatensis	1863	Chamaecyparis	Cupressaceae	Pinales	namerica	gymno	48.79111611	14.78309417	am	83	705	27020	5	510	0.333333333	0.166666667
NH04	39	519	7	68	0.179487179	0.131021195	14	190	0.358974359	0.366088632	NH	28	Abies_concolor	1910	Abies	Pinaceae	Pinales	namerica	gymno	48.79148278	14.78155611	ecm	83	705	27020	5	510	0.333333333	0.166666667
NH05	59	1656	0	0	0	0	30	992	0.508474576	0.599033816	NH	28	Robinia_pseudoacacia	1710	Robinia	Fabaceae	Fabales	namerica	angio	48.79164278	14.78125083	am	85	669	27020	5	510	0.333333333	0.166666667
NH06	63	1531	14	959	0.222222222	0.626387982	26	284	0.412698413	0.185499673	NH	28	Ginkgo_biloba	1809	Ginkgo	Ginkgoaceae	Ginkgoales	asia	gymno	48.790435	14.78206472	am	83	705	27020	5	510	0.333333333	0.166666667
NH07	26	203	6	98	0.230769231	0.482758621	6	36	0.230769231	0.177339901	NH	28	Picea_pungens	1910	Picea	Pinaceae	Pinales	namerica	gymno	48.79045	14.78282111	ecm	83	705	27020	5	510	0.333333333	0.166666667
NOSV02	29	366	12	181	0.413793103	0.494535519	12	164	0.413793103	0.448087432	NOSV	29	Pseudotsuga_menziesii	1830	Pseudotsuga	Pinaceae	Pinales	namerica	gymno	49.03327278	17.80021083	ecm	94	604	32182	6	310	0.571428571	0.285714286
NOSV03	45	1207	2	12	0.044444444	0.009942005	33	1143	0.733333333	0.946975973	NOSV	29	Ginkgo_biloba	1809	Ginkgo	Ginkgoaceae	Ginkgoales	asia	gymno	49.03348389	17.79995278	am	94	604	32182	6	310	0.571428571	0.285714286
NOSV04	41	2147	4	89	0.097560976	0.04145319	30	2026	0.731707317	0.943642292	NOSV	29	Picea_pungens	1910	Picea	Pinaceae	Pinales	namerica	gymno	49.034075	17.79946694	ecm	94	595	32182	6	310	0.571428571	0.285714286
NOSV05	61	1839	0	0	0	0	48	1713	0.786885246	0.931484502	NOSV	29	Catalpa_bignonioides	1785	Catalpa	Bignoniaceae	Lamiales	namerica	angio	49.03379083	17.79919278	am	94	595	32182	6	310	0.571428571	0.285714286
NOSV06	39	1964	8	1141	0.205128205	0.58095723	19	241	0.487179487	0.122708758	NOSV	29	Picea_pungens	1910	Picea	Pinaceae	Pinales	namerica	gymno	49.03402083	17.80063694	ecm	94	604	32182	6	310	0.571428571	0.285714286
NOSV07	43	1218	0	0	0	0	33	1139	0.76744186	0.935139573	NOSV	29	Liriodendron_tulipifera	1785	Liriodendron	Altingiaceae	Saxifragales	namerica	angio	49.03365889	17.80095889	am	94	604	32182	6	310	0.571428571	0.285714286
NOSV08	48	1741	12	1337	0.25	0.767949454	24	303	0.5	0.174037909	NOSV	29	Abies_concolor	1910	Abies	Pinaceae	Pinales	namerica	gymno	49.03351778	17.80111889	ecm	94	604	32182	6	310	0.571428571	0.285714286
OR01	50	356	6	27	0.12	0.075842697	24	232	0.48	0.651685393	OR	30	Chamaecyparis_lawsoniana	1859	Chamaecyparis	Cupressaceae	Pinales	namerica	gymno	49.51221389	14.16906333	am	88	493	75000	11	375	0.470588235	0.176470588
OR02	59	3037	7	290	0.118644068	0.095488969	28	1394	0.474576271	0.459005598	OR	30	Abies_koreana	1934	Abies	Pinaceae	Pinales	asia	gymno	49.51214556	14.16903111	ecm	88	493	75000	11	375	0.470588235	0.176470588
OR03	27	312	0	0	0	0	16	263	0.592592593	0.842948718	OR	30	Magnolia_x_soulangeana	1844	Magnolia	Magnoliaceae	Magnoliales	hybrid	angio	49.51234111	14.16874389	am	88	493	75000	11	375	0.470588235	0.176470588
OR04	66	3648	0	0	0	0	47	3439	0.712121212	0.942708333	OR	30	Sophora_japonica	1835	Sophora	Fabaceae	Fabales	asia	angio	49.51213889	14.16805306	am	88	493	75000	11	375	0.470588235	0.176470588
OR05	64	2000	4	23	0.0625	0.0115	27	1136	0.421875	0.568	OR	30	Chamaecyparis_lawsoniana	1859	Chamaecyparis	Cupressaceae	Pinales	namerica	gymno	49.51198361	14.16828806	am	88	493	75000	11	375	0.470588235	0.176470588
OR06	32	553	6	158	0.1875	0.285714286	13	271	0.40625	0.49005425	OR	30	Thuja_plicata	1844	Thuja	Cupressaceae	Pinales	namerica	gymno	49.511635	14.1675675	am	88	493	75000	11	375	0.470588235	0.176470588
OR07	56	1387	13	778	0.232142857	0.560922855	24	347	0.428571429	0.250180245	OR	30	Pseudotsuga_menziesii	1830	Pseudotsuga	Pinaceae	Pinales	namerica	gymno	49.51166444	14.16766222	ecm	88	493	75000	11	375	0.470588235	0.176470588
OR08	21	627	1	3	0.047619048	0.004784689	12	526	0.571428571	0.83891547	OR	30	Acer_palmatum	1835	Acer	Sapindaceae	Sapindales	asia	angio	49.51172722	14.16759139	am	88	493	75000	11	375	0.470588235	0.176470588
OR09	41	530	2	12	0.048780488	0.022641509	32	491	0.780487805	0.926415094	OR	30	Ginkgo_biloba	1809	Ginkgo	Ginkgoaceae	Ginkgoales	asia	gymno	49.51228361	14.16731361	am	88	493	75000	11	375	0.470588235	0.176470588
OR10	70	1808	7	106	0.1	0.058628319	36	1420	0.514285714	0.78539823	OR	30	Thuja_plicata	1844	Thuja	Cupressaceae	Pinales	namerica	gymno	49.51231056	14.16744528	am	88	493	75000	11	375	0.470588235	0.176470588
OR11	16	485	9	452	0.5625	0.931958763	5	21	0.3125	0.043298969	OR	30	Pinus_strobus	1785	Pinus	Pinaceae	Pinales	namerica	gymno	49.51222639	14.18288167	ecm	88	495	75000	11	375	0.470588235	0.176470588
OR12	41	2164	5	911	0.12195122	0.420979667	23	999	0.56097561	0.461645102	OR	30	Pseudotsuga_menziesii	1830	Pseudotsuga	Pinaceae	Pinales	namerica	gymno	49.51123556	14.16715444	ecm	88	493	75000	11	375	0.470588235	0.176470588
OR13	18	243	9	197	0.5	0.810699588	6	34	0.333333333	0.139917695	OR	30	Pseudotsuga_menziesii	1830	Pseudotsuga	Pinaceae	Pinales	namerica	gymno	49.51121333	14.16720444	ecm	88	493	75000	11	375	0.470588235	0.176470588
OR14	55	5779	8	4230	0.145454545	0.731960547	25	1164	0.454545455	0.201418931	OR	30	Tsuga_canadensis	1812	Tsuga	Pinaceae	Pinales	namerica	gymno	49.51104167	14.16706722	ecm	88	493	75000	11	375	0.470588235	0.176470588
OR16	34	971	5	637	0.147058824	0.656024717	15	274	0.441176471	0.282183316	OR	30	Chamaecyparis_lawsoniana	1859	Chamaecyparis	Cupressaceae	Pinales	namerica	gymno	49.51050944	14.18211056	am	88	495	75000	11	375	0.470588235	0.176470588
OR17	55	1721	9	638	0.163636364	0.370714701	20	323	0.363636364	0.18768158	OR	30	Abies_concolor	1910	Abies	Pinaceae	Pinales	namerica	gymno	49.51054528	14.18227889	ecm	88	495	75000	11	375	0.470588235	0.176470588
OR18	31	476	1	40	0.032258065	0.084033613	19	307	0.612903226	0.644957983	OR	30	Abies_concolor	1910	Abies	Pinaceae	Pinales	namerica	gymno	49.51055278	14.18217722	ecm	88	495	75000	11	375	0.470588235	0.176470588
PASK01	67	2318	2	63	0.029850746	0.027178602	43	2026	0.641791045	0.874029336	PASK	31	Liriodendron_tulipifera	1785	Liriodendron	Altingiaceae	Saxifragales	namerica	angio	49.73212583	18.29422583	am	91	794	174960	5	250	0.5	0.5
PASK03	53	1565	12	888	0.226415094	0.567412141	27	424	0.509433962	0.270926518	PASK	31	Ginkgo_biloba	1809	Ginkgo	Ginkgoaceae	Ginkgoales	asia	gymno	49.73182	18.29455583	am	91	794	174960	5	250	0.5	0.5
PASK04	63	1538	17	1003	0.26984127	0.652145644	30	289	0.476190476	0.187906372	PASK	31	Pinus_strobus	1785	Pinus	Pinaceae	Pinales	namerica	gymno	49.73138083	18.29542889	ecm	91	794	174960	5	250	0.5	0.5
PASK05	68	2057	12	992	0.176470588	0.482255712	34	910	0.5	0.442391833	PASK	31	Pinus_strobus	1785	Pinus	Pinaceae	Pinales	namerica	gymno	49.73012778	18.29488	ecm	91	794	174960	5	250	0.5	0.5
PASK06	58	1416	10	692	0.172413793	0.488700565	23	410	0.396551724	0.289548023	PASK	31	Quercus_rubra	1799	Quercus	Fagaceae	Fagales	namerica	angio	49.730595	18.29460583	ecm	91	794	174960	5	250	0.5	0.5
PASK07	44	1468	11	747	0.25	0.508855586	22	590	0.5	0.401907357	PASK	31	Liriodendron_tulipifera	1785	Liriodendron	Altingiaceae	Saxifragales	namerica	angio	49.73131694	18.29456694	am	91	794	174960	5	250	0.5	0.5
PB01	28	532	0	0	0	0	22	503	0.785714286	0.945488722	PB	32	Sophora_japonica	1835	Sophora	Fabaceae	Fabales	asia	angio	50.21293833	14.4375575	am	89	534	32885	5	270	0.714285714	0.285714286
PB03	37	1031	5	139	0.135135135	0.134820563	18	684	0.486486486	0.66343356	PB	32	Abies_concolor	1910	Abies	Pinaceae	Pinales	namerica	gymno	50.212285	14.43784611	ecm	89	534	32885	5	270	0.714285714	0.285714286
PB04	46	456	7	29	0.152173913	0.063596491	22	269	0.47826087	0.589912281	PB	32	Pseudotsuga_menziesii	1830	Pseudotsuga	Pinaceae	Pinales	namerica	gymno	50.21218778	14.43747139	ecm	89	534	32885	5	270	0.714285714	0.285714286
PB05	41	653	11	161	0.268292683	0.246554364	14	247	0.341463415	0.378254211	PB	32	Pseudotsuga_menziesii	1830	Pseudotsuga	Pinaceae	Pinales	namerica	gymno	50.2120825	14.43713	ecm	89	534	32885	5	270	0.714285714	0.285714286
PB06	41	1413	8	1045	0.195121951	0.739561217	13	161	0.317073171	0.113941967	PB	32	Pinus_strobus	1785	Pinus	Pinaceae	Pinales	namerica	gymno	50.21190306	14.43715222	ecm	89	534	32885	5	270	0.714285714	0.285714286

sample_ID	total_sps.sym	total_seq.sym	ecm_sps	ecm_seq	ecm_share_sps.sym	ecm_share.sym	patho_sps	patho_seq	patho_share_sps.sym	patho_share.sym	park	park_nr	pl_species	pl_species_age_cr	pl_genus	pl_family	pl_order	pl_origin_cont	plant_type	latitude	longitude	m_type	mat	map	area	alien.treeSR	altitude	ecm.s	angio.s
PB07	43	1125	3	10	0.069767442	0.008888889	18	932	0.418604651	0.828444444	PB	32	Liriodendron_tulipifera	1785	Liriodendron	Altingiaceae	Saxifragales	namerica	angio	50.21184833	14.43792611	am	89	534	32885	5	270	0.714285714	0.285714286
PB08	75	2097	10	424	0.133333333	0.20219361	40	1422	0.533333333	0.678111588	PB	32	Pinus_strobus	1785	Pinus	Pinaceae	Pinales	namerica	gymno	50.21201639	14.43815361	ecm	89	534	32885	5	270	0.714285714	0.285714286
PL01	52	2850	0	0	0	0	25	2378	0.480769231	0.834385965	PL	33	Platycladus_orientalis	1785	Platycladus	Cupressaceae	Pinales	asia	gymno	50.56002306	14.20050472	am	90	520	88310	9	225	0.1	0.6
PL02	50	1782	0	0	0	0	31	1561	0.62	0.875982043	PL	33	Thuja_occidentalis	1785	Thuja	Cupressaceae	Pinales	namerica	gymno	50.56003194	14.20088028	am	90	520	88310	9	225	0.1	0.6
PL03	106	16899	10	13421	0.094339623	0.794189005	48	1891	0.452830189	0.111900112	PL	33	Platanus_×_acerifolia	1835	Platanus	Platanaceae	Proteales	hybrid	angio	50.56046417	14.20103694	am	90	520	88310	9	225	0.1	0.6
PL04	91	12184	10	9595	0.10989011	0.787508207	45	1915	0.494505495	0.157173342	PL	33	Robinia_pseudoacacia	1710	Robinia	Fabaceae	Fabales	namerica	angio	50.56055667	14.20087444	am	90	520	88310	9	225	0.1	0.6
PL05	53	1865	2	648	0.037735849	0.347453083	32	1041	0.603773585	0.558176944	PL	33	Pseudotsuga_menziesii	1830	Pseudotsuga	Pinaceae	Pinales	namerica	gymno	50.56069806	14.20087167	ecm	90	520	88310	9	225	0.1	0.6
PL07	86	4564	2	93	0.023255814	0.020376862	50	2970	0.581395349	0.650744961	PL	33	Juglans_nigra	1835	Juglans	Juglandaceae	Fagales	namerica	angio	50.56038167	14.2020525	am	90	520	88310	9	225	0.1	0.6
PL09	42	912	2	8	0.047619048	0.00877193	28	800	0.666666667	0.877192982	PL	33	Ginkgo_biloba	1809	Ginkgo	Ginkgoaceae	Ginkgoales	asia	gymno	50.56060972	14.20202611	am	90	520	88310	9	225	0.1	0.6
PL11	34	429	0	0	0	0	19	260	0.558823529	0.606060606	PL	33	Catalpa_bignonioides	1785	Catalpa	Bignoniaceae	Lamiales	namerica	angio	50.55949306	14.20192361	am	90	520	88310	9	225	0.1	0.6
PL12	30	874	0	0	0	0	24	792	0.8	0.90617849	PL	33	Paulownia_tomentosa	1844	Paulownia	Paulowniaceae	Lamiales	asia	angio	50.55951833	14.20059	am	90	520	88310	9	225	0.1	0.6
PL13	56	2186	0	0	0	0	28	1801	0.5	0.823879231	PL	33	Paulownia_tomentosa	1844	Paulownia	Paulowniaceae	Lamiales	asia	angio	50.55942917	14.20045167	am	90	520	88310	9	225	0.1	0.6
PR01	48	2350	5	61	0.104166667	0.025957447	34	2237	0.708333333	0.951914894	PR	34	Pseudotsuga_menziesii	1830	Pseudotsuga	Pinaceae	Pinales	namerica	gymno	49.99570389	14.56647389	ecm	88	554	1340000	18	325	0.379310345	0.655172414
PR02	35	835	8	509	0.228571429	0.609580838	22	303	0.628571429	0.362874251	PR	34	Pseudotsuga_menziesii	1830	Pseudotsuga	Pinaceae	Pinales	namerica	gymno	49.99572278	14.56639361	ecm	88	554	1340000	18	325	0.379310345	0.655172414
PR03	66	592	3	18	0.045454545	0.030405405	32	328	0.484848485	0.554054054	PR	34	Sophora_japonica	1835	Sophora	Fabaceae	Fabales	asia	angio	49.99408889	14.56351472	am	88	554	1340000	18	325	0.379310345	0.655172414
PR04	75	960	2	9	0.026666667	0.009375	38	526	0.506666667	0.547916667	PR	34	Sophora_japonica	1835	Sophora	Fabaceae	Fabales	asia	angio	49.99403639	14.56347917	am	88	554	1340000	18	325	0.379310345	0.655172414
PR05	62	2296	19	1768	0.306451613	0.770034843	20	324	0.322580645	0.141114983	PR	34	Juglans_nigra	1835	Juglans	Juglandaceae	Fagales	namerica	angio	49.99470361	14.56454222	am	88	554	1340000	18	325	0.379310345	0.655172414
PR06	42	1308	9	999	0.214285714	0.763761468	13	105	0.30952381	0.080275229	PR	34	Tsuga_canadensis	1812	Tsuga	Pinaceae	Pinales	namerica	gymno	49.99396972	14.56166017	ecm	88	554	1340000	18	325	0.379310345	0.655172414
PR07	69	1159	2	47	0.028985507	0.0405522	37	769	0.536231884	0.66350302	PR	34	Liriodendron_tulipifera	1785	Liriodendron	Altingiaceae	Saxifragales	namerica	angio	49.99367722	14.55887694	am	88	554	1340000	18	325	0.379310345	0.655172414
PR08	61	3329	17	1992	0.278688525	0.598377891	16	383	0.262295082	0.115049564	PR	34	Pseudotsuga_menziesii	1830	Pseudotsuga	Pinaceae	Pinales	namerica	gymno	49.99358556	14.55817722	ecm	88	546	1340000	18	325	0.379310345	0.655172414
PR09	35	2185	14	2009	0.4	0.919450801	8	41	0.228571429	0.018764302	PR	34	Pseudotsuga_menziesii	1830	Pseudotsuga	Pinaceae	Pinales	namerica	gymno	49.99278861	14.55447167	ecm	88	546	1340000	18	325	0.379310345	0.655172414
PR10	70	10219	18	9100	0.257142857	0.890498092	20	288	0.285714286	0.028182797	PR	34	Picea_pungens	1910	Picea	Pinaceae	Pinales	namerica	gymno	49.98920444	14.54725111	ecm	87	558	1340000	18	325	0.379310345	0.655172414
PR11	47	1912	9	1115	0.191489362	0.583158996	16	403	0.340425532	0.210774059	PR	34	Picea_pungens	1910	Picea	Pinaceae	Pinales	namerica	gymno	49.98911861	14.54717639	ecm	87	558	1340000	18	325	0.379310345	0.655172414
PR12	64	3786	2	12	0.03125	0.003169572	34	2776	0.53125	0.733227681	PR	34	Robinia_pseudoacacia	1710	Robinia	Fabaceae	Fabales	namerica	angio	49.98896722	14.54480889	am	87	558	1340000	18	325	0.379310345	0.655172414
PR13	30	426	2	42	0.066666667	0.098591549	10	159	0.333333333	0.373239437	PR	34	Chamaecyparis_pisifera	1865	Chamaecyparis	Cupressaceae	Pinales	asia	gymno	49.98857083	14.54351417	am	87	558	1340000	18	325	0.379310345	0.655172414
PR18	53	6827	15	6497	0.283018868	0.951662516	14	78	0.264150943	0.011425223	PR	34	Platanus_occidentalis	1785	Platanus	Platanaceae	Proteales	namerica	angio	49.98823944	14.54758139	am	87	558	1340000	18	325	0.379310345	0.655172414
PR19	28	393	15	328	0.535714286	0.834605598	11	53	0.392857143	0.134860051	PR	34	Catalpa_bignonioides	1785	Catalpa	Bignoniaceae	Lamiales	namerica	angio	49.99011722	14.55127389	am	87	562	1340000	18	325	0.379310345	0.655172414
PR20	97	9970	28	8600	0.288659794	0.862587763	32	736	0.329896907	0.073821464	PR	34	Catalpa_bignonioides	1785	Catalpa	Bignoniaceae	Lamiales	namerica	angio	49.99034694	14.55180417	am	87	562	1340000	18	325	0.379310345	0.655172414
PR21	106	5684	8	142	0.075471698	0.024982407	52	5067	0.490566038	0.891449683	PR	34	Catalpa_bignonioides	1785	Catalpa	Bignoniaceae	Lamiales	namerica	angio	49.99530917	14.55639444	am	88	546	1340000	18	325	0.379310345	0.655172414
PR22	97	15078	16	11996	0.164948454	0.795596233	44	639	0.453608247	0.042379626	PR	34	Castanea_sativa	1562	Castanea	Fagaceae	Fagales	europe	angio	49.99482083	14.5597925	ecm	88	554	1340000	18	325	0.379310345	0.655172414
PR23	71	3432	17	2494	0.23943662	0.726689977	36	759	0.507042254	0.221153846	PR	34	Carya_tomentosa	1865	Carya	Juglandaceae	Fagales	namerica	angio	49.99614083	14.55980417	ecm	88	554	1340000	18	325	0.379310345	0.655172414
PR24	84	3031	1	4	0.011904762	0.001319696	47	2624	0.55952381	0.865720884	PR	34	Phellodendron_amurense	1911	Phellodendron	Rutaceae	Sapindales	asia	angio	49.99691306	14.56140667	am	88	554	1340000	18	325	0.379310345	0.655172414
PR25	39	1932	12	1386	0.307692308	0.717391304	19	496	0.487179487	0.256728778	PR	34	Quercus_velutina	1852	Quercus	Fagaceae	Fagales	namerica	angio	49.99756861	14.55979222	ecm	88	554	1340000	18	325	0.379310345	0.655172414
PR26	43	543	4	62	0.093023256	0.114180479	16	211	0.372093023	0.388581952	PR	34	Chamaecyparis_nootkatensis	1863	Chamaecyparis	Cupressaceae	Pinales	namerica	gymno	49.99846306	14.55933	am	88	554	1340000	18	325	0.379310345	0.655172414
PR27	74	3586	2	1753	0.027027027	0.48884551	49	1546	0.662162162	0.431121026	PR	34	Sophora_japonica	1835	Sophora	Fabaceae	Fabales	asia	angio	49.99938333	14.55918083	am	88	554	1340000	18	325	0.379310345	0.655172414
PR28	64	1446	0	0	0	0	36	1224	0.5625	0.846473029	PR	34	Gleditsia_triacanthos	1785	Gleditsia	Fabaceae	Fabales	namerica	angio	49.99998083	14.55876944	am	89	542	1340000	18	325	0.379310345	0.655172414
PR29	84	2231	3	55	0.035714286	0.024652622	47	1327	0.55952381	0.594800538	PR	34	Thuja_plicata	1844	Thuja	Cupressaceae	Pinales	namerica	gymno	50.00001111	14.55724944	am	89	527	1340000	18	325	0.379310345	0.655172414
PR30	45	596	7	303	0.155555556	0.508389262	19	143	0.422222222	0.239932886	PR	34	Quercus_imbricaria	1880	Quercus	Fagaceae	Fagales	namerica	angio	49.99973083	14.55735806	ecm	88	546	1340000	18	325	0.379310345	0.655172414
PR32	60	968	1	30	0.016666667	0.030991736	29	553	0.483333333	0.571280992	PR	34	Catalpa_bignonioides	1785	Catalpa	Bignoniaceae	Lamiales	namerica	angio	49.98781361	14.56078889	am	87	564	1340000	18	325	0.379310345	0.655172414
PR33	59	2642	2	13	0.033898305	0.004920515	30	2329	0.508474576	0.881529145	PR	34	Robinia_pseudoacacia	1710	Robinia	Fabaceae	Fabales	namerica	angio	49.98913306	14.5613825	am	87	564	1340000	18	325	0.379310345	0.655172414
PR34	47	1350	1	54	0.021276596	0.04	21	998	0.446808511	0.739259259	PR	34	Robinia_pseudoacacia	1710	Robinia	Fabaceae	Fabales	namerica	angio	49.9890775	14.56139417	am	87	564	1340000	18	325	0.379310345	0.655172414
SL01	24	433	0	0	0	0	21	419	0.875	0.967667436	SL	35	Sophora_japonica	1835	Sophora	Fabaceae	Fabales	asia	angio	49.91921222	15.81058722	am	90	540	179805	19	287	0.347826087	0.608695652
SL02	46	2308	4	142	0.086956522	0.06152513	30	2105	0.652173913	0.912045061	SL	35	Robinia_pseudoacacia	1710	Robinia	Fabaceae	Fabales	namerica	angio	49.91806333	15.80963556	am	90	540	179805	19	287	0.347826087	0.608695652
SL03	33	1073	2	45	0.060606061	0.04193849	22	940	0.666666667	0.876048462	SL	35	Magnolia_x_soulangeana	1844	Magnolia	Magnoliaceae	Magnoliales	hybrid	angio	49.91813556	15.80993056	am	90	540	179805	19	287	0.347826087	0.608695652
SL04	53	1993	1	389	0.018867925	0.195183141	39	1547	0.735849057	0.776216759	SL	35	Magnolia_x_soulangeana	1844	Magnolia	Magnoliaceae	Magnoliales	hybrid	angio	49.91808167	15.80997306	am	90	540	179805	19	287	0.347826087	0.608695652
SL05	72	2345	3	10	0.041666667	0.004264392	42	1944	0.583333333	0.828997868	SL	35	Catalpa_bignonioides	1785	Catalpa	Bignoniaceae	Lamiales	namerica	angio	49.91743472	15.80950806	am	90	540	179805	19	287	0.347826087	0.608695652
SL07	64	1846	0	0	0	0	47	1734	0.734375	0.939328277	SL	35	Thuja_plicata	1844	Thuja	Cupressaceae	Pinales	namerica	gymno	49.91727639	15.80926528	am	90	540	179805	19	287	0.347826087	0.608695652
SL08	45	1054	2	94	0.044444444	0.089184061	27	823	0.6	0.780834915	SL	35	Abies_pinsapo	1845	Abies	Pinaceae	Pinales	europe	gymno	49.9177025	15.80897472	ecm	90	540	179805	19	287	0.347826087	0.608695652
SL10	44	603	7	31	0.159090909	0.051409619	22	330	0.5	0.547263682	SL	35	Picea_pungens	1910	Picea	Pinaceae	Pinales	namerica	gymno	49.91788694	15.80904444	ecm	90	540	179805	19	287	0.347826087	0.608695652
SL12	66	1834	2	7	0.03030303	0.003816794	35	1433	0.53030303	0.781352236	SL	35	Robinia_pseudoacacia	1710	Robinia	Fabaceae	Fabales	namerica	angio	49.93327278	15.80707528	am	91	562	179805	19	287	0.347826087	0.608695652
SL13	77	3410	8	42	0.103896104	0.012316716	41	3174	0.532467532	0.930791789	SL	35	Gymnocladus_dioicus	1844	Gymnocladus	Fabaceae	Fabales	namerica	angio	49.91657667	15.80736139	am	87	570	179805	19	287	0.347826087	0.608695652
SL14	116	4120	12	194	0.103448276	0.047087379	50	1070	0.431034483	0.259708738	SL	35	Parro�a_persica	1927	Parro�a	Hamamelidaceae	Saxifragales	asia	angio	49.91646389	15.806955	am	87	570	179805	19	287	0.347826087	0.608695652
SL15	47	1262	2	12	0.042553191	0.009508716	29	1000	0.617021277	0.792393027	SL	35	Pinus_contorta	1965	Pinus	Pinaceae	Pinales	namerica	gymno	49.91601944	15.80704472	ecm	87	570	179805	19	287	0.347826087	0.608695652
SL16	61	2025	12	1177	0.196721311	0.581234568	25	356	0.409836066	0.175802469	SL	35	Chamaecyparis_lawsoniana	1859	Chamaecyparis	Cupressaceae	Pinales	namerica	gymno	49.91597861	15.80689556	am	87	570	179805	19	287	0.347826087	0.608695652
SL17	11	52	2	12	0.181818182	0.230769231	1	3	0.090909091	0.057692308	SL	35	Platanus_×_acerifolia	1835	Platanus	Platanaceae	Proteales	hybrid	angio	49.91558722	15.80705056	am	87	570	179805	19	287	0.347826087	0.608695652
SL18	72	1244	8	519	0.111111111	0.417202572	32	409	0.444444444	0.328778135	SL	35	Tsuga_canadensis	1812	Tsuga	Pinaceae	Pinales	namerica	gymno	49.91555056	15.80736722	ecm	87	570	179805	19	287	0.347826087	0.608695652
SL19	55	978	4	87	0.072727273	0.088957055	34	700	0.618181818	0.715746421	SL	35	Platanus_×_acerifolia	1835	Platanus	Platanaceae	Proteales	hybrid	angio	49.91620861	15.8081025	am	87	570	179805	19	287	0.347826087	0.608695652
SL21	52	1399	2	483	0.038461538	0.345246605	23	314	0.442307692	0.224446033	SL	35	Picea_orientalis	1813	Picea	Pinaceae	Pinales	asia	gymno	49.91633389	15.80846694	ecm	90	537	179805	19	287	0.347826087	0.608695652
SL22	48	919	7	209	0.145833333	0.22742111	29	612	0.604166667	0.66594124	SL	35	Abies_lasiocarpa	1865	Abies	Pinaceae	Pinales	namerica	gymno	49.91627583	15.80846639	ecm	90	537	179805	19	287	0.347826087	0.608695652
SL23	47	678	10	169	0.212765957	0.249262537	29	374	0.617021277	0.551622419	SL	35	Sophora_japonica	1835	Sophora	Fabaceae	Fabales	asia	angio	49.91652306	15.8094975	am	90	537	179805	19	287	0.347826087	0.608695652
SL25	82	4519	1	4	0.012195122	0.000885152	45	3964	0.548780488	0.877185218	SL	35	Pinus_strobus	1785	Pinus	Pinaceae	Pinales	namerica	gymno	49.91647194	15.81106222	ecm	90	537	179805	19	287	0.347826087	0.608695652
SL26	66	1009	4	105	0.060606061	0.104063429	43	653	0.651515152	0.647175421	SL	35	Liriodendron_tulipifera	1785	Liriodendron	Altingiaceae	Saxifragales	namerica	angio	49.91892417	15.80949611	am	90	540	179805	19	287	0.347826087	0.608695652
SL27	72	3297	15	1624	0.208333333	0.492569002	33	1374	0.458333333	0.416742493	SL	35	Tilia_americana	1835	Tilia	Malvaceae	Malvales	namerica	angio	49.91891472	15.80923	ecm	90	540	179805	19	287	0.347826087	0.608695652
SL28	107	7286	16	5081	0.14953271	0.697364809	50	1727	0.46728972	0.23702992	SL	35	Aesculus_flava	1835	Aesculus	Sapindaceae	Sapindales	namerica	angio	49.91878222	15.80914167	am	90	540	179805	19	287	0.347826087	0.608695652
SYCH01	39	694	5	52	0.128205128	0.074927954	20	231	0.512820513	0.332853026	SYCH	36	Platanus_×_acerifolia	1835	Platanus	Platanaceae	Proteales	hybrid	angio	50.62595444	15.09076806	am	81	749	203030	11	375	0.615384615	0.461538462
SYCH02	31	473	1	7	0.032258065	0.014799154	21	320	0.677419355	0.67653277	SYCH	36	Picea_pungens	1910	Picea	Pinaceae	Pinales	namerica	gymno	50.62570556	15.09015667	ecm	81	749	203030	11	375	0.615384615	0.461538462
SYCH03	37	798	2	59	0.054054054	0.073934837	26	581	0.702702703	0.728070175	SYCH	36	Pterocarya_fraxinifolia	1844	Pterocarya	Juglandaceae	Fagales	asia	angio	50.62541167	15.0907325	am	81	749	203030	11	375	0.615384615	0.461538462
SYCH05	43	2270	8	1905	0.186046512	0.839207048	24	320	0.558139535	0.140969163	SYCH	36	Liriodendron_tulipifera	1785	Liriodendron	Altingiaceae	Saxifragales	namerica	angio	50.62554611	15.09166278	am	80	778	203030	11	375	0.615384615	0.461538462
SYCH07	46	2160	7	1150	0.152173913	0.532407407	21	655	0.456521739	0.303240741	SYCH	36	Quercus_rubra	1799	Quercus	Fagaceae	Fagales	namerica	angio	50.62597861	15.09371944	ecm	80	778	203030	11	375	0.615384615	0.461538462
SYCH08	34	1072	17	921	0.5	0.859141791	6	31	0.176470588	0.02891791	SYCH	36	Pinus_strobus	1785	Pinus	Pinaceae	Pinales	namerica	gymno	50.62667778	15.09361111	ecm	80	778	203030	11	375	0.615384615	0.461538462
SYCH09	38	656	9	280	0.236842105	0.426829268	8	96	0.210526316	0.146341463	SYCH	36	Pinus_strobus	1785	Pinus	Pinaceae	Pinales	namerica	gymno	50.62716111	15.09311194	ecm	80	778	203030	11	375	0.615384615	0.461538462
SYCH10	23	654	10	563	0.434782609	0.860856269	6	34	0.260869565	0.051987768	SYCH	36	Pseudotsuga_menziesii	1830	Pseudotsuga	Pinaceae	Pinales	namerica	gymno	50.62739889	15.09077333	ecm	81	749	203030	11	375	0.615384615	0.461538462
SYCH11	47	1297	6	187	0.127659574	0.144178874	28	945	0.595744681	0.728604472	SYCH	36	Ginkgo_biloba	1809	Ginkgo	Ginkgoaceae	Ginkgoales	asia	gymno	50.6260975	15.08980333	am	81	749	203030	11	375	0.615384615	0.461538462
SYCH12	19	304	1	5	0.052631579	0.016447368	10	218	0.526315789	0.717105263	SYCH	36	Pseudotsuga_menziesii	1830	Pseudotsuga	Pinaceae	Pinales	namerica	gymno	50.62611222	15.08996889	ecm	81	749	203030	11	375	0.615384615	0.461538462
SYCH13	37	1112	0	0	0	0	21	839	0.567567568	0.754496403	SYCH	36	Catalpa_bignonioides	1785	Catalpa	Bignoniaceae	Lamiales	namerica	angio	50.62595972	15.08952139	am	81	749	203030	11	375	0.615384615	0.461538462
SYCH14	46	2974	20	2257	0.434782609	0.758910558	15	414	0.326086957	0.139206456	SYCH	36	Castanea_sativa	1562	Castanea	Fagaceae	Fagales	europe	angio	50.62552083	15.08957028	ecm	81	749	203030	11	375	0.615384615	0.461538462
SYCH15	9	87	0	0	0	0	5	27	0.555555556	0.310344828	SYCH	36	Pinus_ponderosa	1845	Pinus	Pinaceae	Pinales	namerica	gymno	50.62572861	15.08806361	ecm	81	749	203030	11	375	0.615384615	0.461538462
TE03	42	1356	1	84	0.023809524	0.061946903	30	1138	0.714285714	0.839233038	TE	37	Pinus_strobus	1785	Pinus	Pinaceae	Pinales	namerica	gymno	49.1857	15.4476	ecm	81	548	75910	6	511	0.875	0.375
TE04	55	1809	14	1354	0.254545455	0.748479823	30	293	0.545454545	0.161967938	TE	37	Quercus_rubra	1799	Quercus	Fagaceae	Fagales	namerica	angio	49.1855	15.4472	ecm	81	548	75910	6	511	0.875	0.375
TE05	50	2893	12	1965	0.24	0.679225717	20	808	0.4	0.27929485	TE	37	Picea_omorica	1906	Picea	Pinaceae	Pinales	europe	gymno	49.1853	15.4456	ecm	81	548	75910	6	511	0.875	0.375

sample_ID	total_sps.sym	total_seq.sym	ecm_sps	ecm_seq	ecm_share_sps.sym	ecm_share.sym	patho_sps	patho_seq	patho_share_sps.sym	patho_share.sym	park	park_nr	pl_species	pl_species_age_cr	pl_genus	pl_family	pl_order	pl_origin_cont	plant_type	latitude	longitude	m_type	mat	map	area	alien.treeSR	altitude	ecm.s	angio.s
TE06	44	553	5	149	0.113636364	0.269439421	18	228	0.409090909	0.412296564	TE	37	Tsuga_canadensis	1812	Tsuga	Pinaceae	Pinales	namerica	gymno	49.1842	15.4491	ecm	81	548	75910	6	511	0.875	0.375
TE07	53	923	4	20	0.075471698	0.021668472	28	795	0.528301887	0.861321777	TE	37	Quercus_rubra	1799	Quercus	Fagaceae	Fagales	namerica	angio	49.1866	15.4492	ecm	81	548	75910	6	511	0.875	0.375
TE08	32	387	3	16	0.09375	0.041343669	17	262	0.53125	0.677002584	TE	37	Pinus_strobus	1785	Pinus	Pinaceae	Pinales	namerica	gymno	49.1863	15.4502	ecm	81	554	75910	6	511	0.875	0.375
TE09	43	714	2	59	0.046511628	0.082633053	25	573	0.581395349	0.802521008	TE	37	Pseudotsuga_menziesii	1830	Pseudotsuga	Pinaceae	Pinales	namerica	gymno	49.1865	15.4496	ecm	81	548	75910	6	511	0.875	0.375
TE10	36	912	1	5	0.027777778	0.005482456	26	853	0.722222222	0.935307018	TE	37	Cercidiphyllum_japonicum	1910	Cercidiphyllum	Cercidiphyllaceae	Saxifragales	asia	angio	49.1864	15.4491	am	81	548	75910	6	511	0.875	0.375
VE01	63	1489	2	6	0.031746032	0.00402955	37	1228	0.587301587	0.824714574	VE	38	Platanus_×_acerifolia	1835	Platanus	Platanaceae	Proteales	hybrid	angio	50.2754	14.32942389	am	94	456	624000	5	180	0	0.75
VE02	36	529	0	0	0	0	26	406	0.722222222	0.767485822	VE	38	Robinia_pseudoacacia	1710	Robinia	Fabaceae	Fabales	namerica	angio	50.27537389	14.32957417	am	94	456	624000	5	180	0	0.75
VE03	44	677	0	0	0	0	27	483	0.613636364	0.713441654	VE	38	Robinia_pseudoacacia	1710	Robinia	Fabaceae	Fabales	namerica	angio	50.27543389	14.32970361	am	94	456	624000	5	180	0	0.75
VE04	37	735	2	54	0.054054054	0.073469388	23	587	0.621621622	0.798639456	VE	38	Ginkgo_biloba	1809	Ginkgo	Ginkgoaceae	Ginkgoales	asia	gymno	50.27727333	14.33039611	am	94	456	624000	5	180	0	0.75
VE06	68	3928	3	1757	0.044117647	0.447301426	37	1319	0.544117647	0.335794297	VE	38	Ginkgo_biloba	1809	Ginkgo	Ginkgoaceae	Ginkgoales	asia	gymno	50.27750972	14.33076	am	94	456	624000	5	180	0	0.75
VE07	60	2628	1	70	0.016666667	0.026636225	42	2345	0.7	0.892313546	VE	38	Sophora_japonica	1835	Sophora	Fabaceae	Fabales	asia	angio	50.27669972	14.33000167	am	94	456	624000	5	180	0	0.75
VE08	62	4897	0	0	0	0	38	4537	0.612903226	0.926485603	VE	38	Catalpa_bignonioides	1785	Catalpa	Bignoniaceae	Lamiales	namerica	angio	50.27701111	14.32859333	am	94	456	624000	5	180	0	0.75
VE09	62	1776	0	0	0	0	35	857	0.564516129	0.482545045	VE	38	Platanus_×_acerifolia	1835	Platanus	Platanaceae	Proteales	hybrid	angio	50.27548861	14.33042167	am	94	456	624000	5	180	0	0.75
VEHE01	50	1134	0	0	0	0	36	992	0.72	0.874779541	VEHE	39	Pterocarya_fraxinifolia	1844	Pterocarya	Juglandaceae	Fagales	asia	angio	49.97540278	17.73606389	am	84	625	36569	9	335	0.333333333	0.777777778
VEHE02	51	528	0	0	0	0	36	453	0.705882353	0.857954545	VEHE	39	Platanus_×_acerifolia	1835	Platanus	Platanaceae	Proteales	hybrid	angio	49.97515694	17.737775	am	84	625	36569	9	335	0.333333333	0.777777778
VEHE04	37	4876	5	4396	0.135135135	0.901558655	20	117	0.540540541	0.023995078	VEHE	39	Quercus_rubra	1799	Quercus	Fagaceae	Fagales	namerica	angio	49.97582778	17.73805	ecm	84	625	36569	9	335	0.333333333	0.777777778
VEHE05	35	471	4	83	0.114285714	0.176220807	20	260	0.571428571	0.552016985	VEHE	39	Abies_cephalonica	1845	Abies	Pinaceae	Pinales	europe	gymno	49.975625	17.73797694	ecm	84	625	36569	9	335	0.333333333	0.777777778
VEHE06	52	1490	1	8	0.019230769	0.005369128	35	1409	0.673076923	0.945637584	VEHE	39	Gleditsia_triacanthos	1785	Gleditsia	Fabaceae	Fabales	namerica	angio	49.97574	17.73657389	am	84	625	36569	9	335	0.333333333	0.777777778
VEHE07	31	691	1	6	0.032258065	0.008683068	23	658	0.741935484	0.952243126	VEHE	39	Picea_pungens	1910	Picea	Pinaceae	Pinales	namerica	gymno	49.97513889	17.73610083	ecm	84	625	36569	9	335	0.333333333	0.777777778
VEHE08	50	1078	0	0	0	0	31	599	0.62	0.555658627	VEHE	39	Magnolia_kobus	1910	Magnolia	Magnoliaceae	Magnoliales	asia	angio	49.97511083	17.73609583	am	84	625	36569	9	335	0.333333333	0.777777778
VEHE09	38	1889	1	9	0.026315789	0.004764426	27	1746	0.710526316	0.924298571	VEHE	39	Liriodendron_tulipifera	1785	Liriodendron	Altingiaceae	Saxifragales	namerica	angio	49.97518278	17.73583778	am	84	625	36569	9	335	0.333333333	0.777777778
VEHE10	54	3067	0	0	0	0	38	2926	0.703703704	0.954026736	VEHE	39	Catalpa_bignonioides	1785	Catalpa	Bignoniaceae	Lamiales	namerica	angio	49.97538083	17.73565389	am	84	625	36569	9	335	0.333333333	0.777777778
VELO01	58	1810	8	213	0.137931034	0.117679558	28	1434	0.482758621	0.792265193	VELO	40	Ginkgo_biloba	1809	Ginkgo	Ginkgoaceae	Ginkgoales	asia	gymno	50.02375	17.03164083	am	80	657	121300	6	380	0.333333333	0.166666667
VELO02	32	907	0	0	0	0	19	722	0.59375	0.796030871	VELO	40	Liriodendron_tulipifera	1785	Liriodendron	Altingiaceae	Saxifragales	namerica	angio	50.02331194	17.03271583	am	80	657	121300	6	380	0.333333333	0.166666667
VELO03	55	2384	18	1935	0.327272727	0.811661074	18	235	0.327272727	0.098573826	VELO	40	Tsuga_canadensis	1812	Tsuga	Pinaceae	Pinales	namerica	gymno	50.023	17.032635	ecm	80	657	121300	6	380	0.333333333	0.166666667
VELO04	41	2421	0	0	0	0	25	2253	0.609756098	0.930607187	VELO	40	Metasequoia_glyptostroboides	1949	Metasequoia	Cupressaceae	Pinales	asia	gymno	50.02069778	17.030565	am	80	657	121300	6	380	0.333333333	0.166666667
VELO05	55	2075	0	0	0	0	36	1930	0.654545455	0.930120482	VELO	40	Thuja_occidentalis	1785	Thuja	Cupressaceae	Pinales	namerica	gymno	50.02279778	17.03014194	am	80	657	121300	6	380	0.333333333	0.166666667
VELO06	55	1120	9	491	0.163636364	0.438392857	27	522	0.490909091	0.466071429	VELO	40	Pinus_ponderosa	1845	Pinus	Pinaceae	Pinales	namerica	gymno	50.02334389	17.02990889	ecm	80	657	121300	6	380	0.333333333	0.166666667
VES01	37	1243	0	0	0	0	25	1172	0.675675676	0.942880129	VES	41	Platanus_occidentalis	1785	Platanus	Platanaceae	Proteales	namerica	angio	49.5317	17.507105	am	91	619	42303	8	275	0.666666667	0.333333333
VES02	57	1173	5	497	0.087719298	0.423699915	31	479	0.543859649	0.408354646	VES	41	Pinus_jeffreyi	1865	Pinus	Pinaceae	Pinales	namerica	gymno	49.53140278	17.50655389	ecm	91	619	42303	8	275	0.666666667	0.333333333
VES04	17	559	2	72	0.117647059	0.128801431	7	408	0.411764706	0.729874776	VES	41	Picea_pungens	1910	Picea	Pinaceae	Pinales	namerica	gymno	49.53129194	17.50576694	ecm	91	619	42303	8	275	0.666666667	0.333333333
VES05	61	2000	0	0	0	0	36	985	0.590163934	0.4925	VES	41	Catalpa_bignonioides	1785	Catalpa	Bignoniaceae	Lamiales	namerica	angio	49.53079389	17.50543083	am	91	619	42303	8	275	0.666666667	0.333333333
VES06	45	2298	6	712	0.133333333	0.309834639	20	619	0.444444444	0.269364665	VES	41	Castanea_sativa	1562	Castanea	Fagaceae	Fagales	europe	angio	49.53018694	17.50568	ecm	91	619	42303	8	275	0.666666667	0.333333333
VES07	51	1187	0	0	0	0	30	954	0.588235294	0.803706824	VES	41	Pinus_strobus	1785	Pinus	Pinaceae	Pinales	namerica	gymno	49.53012778	17.50586778	ecm	91	619	42303	8	275	0.666666667	0.333333333
VES08	65	2095	7	515	0.107692308	0.245823389	42	1406	0.646153846	0.671121718	VES	41	Pinus_ponderosa	1845	Pinus	Pinaceae	Pinales	namerica	gymno	49.53000083	17.50597	ecm	91	619	42303	8	275	0.666666667	0.333333333
VES09	50	1049	0	0	0	0	35	939	0.7	0.895138227	VES	41	Ginkgo_biloba	1809	Ginkgo	Ginkgoaceae	Ginkgoales	asia	gymno	49.53047694	17.50593889	am	91	619	42303	8	275	0.666666667	0.333333333
VES10	44	1716	1	54	0.022727273	0.031468531	26	1493	0.590909091	0.87004662	VES	41	Pseudotsuga_menziesii	1830	Pseudotsuga	Pinaceae	Pinales	namerica	gymno	49.53002278	17.50576278	ecm	91	619	42303	8	275	0.666666667	0.333333333
VJ01	54	1049	1	3	0.018518519	0.002859867	34	685	0.62962963	0.65300286	VJ	42	Liriodendron_tulipifera	1785	Liriodendron	Altingiaceae	Saxifragales	namerica	angio	49.66899389	14.57859139	am	84	524	159084	15	425	0.476190476	0.523809524
VJ02	35	493	1	6	0.028571429	0.012170385	17	259	0.485714286	0.52535497	VJ	42	Phellodendron_amurense	1911	Phellodendron	Rutaceae	Sapindales	asia	angio	49.669735	14.578075	am	84	524	159084	15	425	0.476190476	0.523809524
VJ03	33	234	6	63	0.181818182	0.269230769	15	102	0.454545455	0.435897436	VJ	42	Pseudotsuga_menziesii	1830	Pseudotsuga	Pinaceae	Pinales	namerica	gymno	49.66984889	14.5777225	ecm	84	524	159084	15	425	0.476190476	0.523809524
VJ04	31	371	11	266	0.35483871	0.716981132	7	34	0.225806452	0.091644205	VJ	42	Pseudotsuga_menziesii	1830	Pseudotsuga	Pinaceae	Pinales	namerica	gymno	49.66990083	14.57794417	ecm	84	524	159084	15	425	0.476190476	0.523809524
VJ05	70	1854	12	1226	0.171428571	0.661272923	21	266	0.3	0.143473571	VJ	42	Sophora_japonica	1835	Sophora	Fabaceae	Fabales	asia	angio	49.67012083	14.57880889	am	84	524	159084	15	425	0.476190476	0.523809524
VJ06	49	980	3	55	0.06122449	0.056122449	20	751	0.408163265	0.766326531	VJ	42	Sophora_japonica	1835	Sophora	Fabaceae	Fabales	asia	angio	49.6701325	14.57898222	am	84	524	159084	15	425	0.476190476	0.523809524
VJ07	43	846	6	249	0.139534884	0.294326241	18	456	0.418604651	0.539007092	VJ	42	Picea_omorica	1906	Picea	Pinaceae	Pinales	europe	gymno	49.67067222	14.57946667	ecm	84	524	159084	15	425	0.476190476	0.523809524
VJ08	19	160	4	87	0.210526316	0.54375	7	35	0.368421053	0.21875	VJ	42	Tsuga_canadensis	1812	Tsuga	Pinaceae	Pinales	namerica	gymno	49.67074833	14.57937972	ecm	84	524	159084	15	425	0.476190476	0.523809524
VJ09	47	2128	10	853	0.212765957	0.400845865	20	1193	0.425531915	0.560620301	VJ	42	Fraxinus_pennsylvanica	1835	Fraxinus	Oleaceae	Lamiales	namerica	angio	49.67074972	14.580065	am	84	524	159084	15	425	0.476190476	0.523809524
VJ10	67	938	6	31	0.089552239	0.033049041	22	296	0.328358209	0.315565032	VJ	42	Chamaecyparis_lawsoniana	1859	Chamaecyparis	Cupressaceae	Pinales	namerica	gymno	49.66993917	14.58102472	am	84	524	159084	15	425	0.476190476	0.523809524
VJ11	21	184	4	77	0.19047619	0.418478261	11	83	0.523809524	0.451086957	VJ	42	Thuja_occidentalis	1785	Thuja	Cupressaceae	Pinales	namerica	gymno	49.67004694	14.58107056	am	84	524	159084	15	425	0.476190476	0.523809524
VJ12	62	1740	14	611	0.225806452	0.351149425	22	694	0.35483871	0.398850575	VJ	42	Abies_grandis	1879	Abies	Pinaceae	Pinales	namerica	gymno	49.66924194	14.58139917	ecm	84	524	159084	15	425	0.476190476	0.523809524
VJ13	13	109	3	61	0.230769231	0.559633028	3	14	0.230769231	0.128440367	VJ	42	Abies_grandis	1879	Abies	Pinaceae	Pinales	namerica	gymno	49.66926694	14.58110917	ecm	84	524	159084	15	425	0.476190476	0.523809524
VJ14	80	5464	32	4301	0.4	0.787152269	23	804	0.2875	0.147144949	VJ	42	Castanea_sativa	1562	Castanea	Fagaceae	Fagales	europe	angio	49.66896472	14.58184694	ecm	84	524	159084	15	425	0.476190476	0.523809524
VJ15	50	7205	16	5784	0.32	0.80277585	21	1232	0.42	0.170992366	VJ	42	Castanea_sativa	1562	Castanea	Fagaceae	Fagales	europe	angio	49.66910944	14.58201278	ecm	84	524	159084	15	425	0.476190476	0.523809524
VJ16	73	2927	19	739	0.260273973	0.252476939	23	1510	0.315068493	0.515886573	VJ	42	Acer_palmatum	1835	Acer	Sapindaceae	Sapindales	asia	angio	49.66885472	14.58244222	am	84	524	159084	15	425	0.476190476	0.523809524
VJ17	51	1036	19	683	0.37254902	0.659266409	10	82	0.196078431	0.079150579	VJ	42	Acer_palmatum	1835	Acer	Sapindaceae	Sapindales	asia	angio	49.66908722	14.58032722	am	84	524	159084	15	425	0.476190476	0.523809524
VJ18	76	5899	15	4804	0.197368421	0.814375318	22	239	0.289473684	0.040515342	VJ	42	Sophora_japonica	1835	Sophora	Fabaceae	Fabales	asia	angio	49.66919556	14.58012556	am	84	524	159084	15	425	0.476190476	0.523809524
VJ19	34	634	1	6	0.029411765	0.009463722	21	197	0.617647059	0.310725552	VJ	42	Platanus_×_acerifolia	1835	Platanus	Platanaceae	Proteales	hybrid	angio	49.66975583	14.58002667	am	84	524	159084	15	425	0.476190476	0.523809524
VJ20	46	1143	1	4	0.02173913	0.003499563	25	886	0.543478261	0.775153106	VJ	42	Pinus_strobus	1785	Pinus	Pinaceae	Pinales	namerica	gymno	49.66927944	14.57981472	ecm	84	524	159084	15	425	0.476190476	0.523809524
VJ21	74	2348	12	173	0.162162162	0.073679727	35	1747	0.472972973	0.744037479	VJ	42	Abies_nordmanniana	1845	Abies	Pinaceae	Pinales	asia	gymno	49.66922917	14.57908	ecm	84	524	159084	15	425	0.476190476	0.523809524
VL03	65	1267	3	16	0.046153846	0.012628256	33	999	0.507692308	0.788476717	VL	43	Gleditsia_triacanthos	1785	Gleditsia	Fabaceae	Fabales	namerica	angio	49.70657694	14.89525167	am	86	539	131344	7	350	0.6	0.3
VL04	52	1581	0	0	0	0	33	1379	0.634615385	0.872232764	VL	43	Gleditsia_triacanthos	1785	Gleditsia	Fabaceae	Fabales	namerica	angio	49.70663278	14.89490361	am	86	539	131344	7	350	0.6	0.3
VL05	64	1943	3	12	0.046875	0.006176016	31	1630	0.484375	0.838908904	VL	43	Aesculus_x_carnea	1835	Aesculus	Sapindaceae	Sapindales	hybrid	angio	49.7066075	14.8942769	am	86	539	131344	7	350	0.6	0.3
VL08	57	1549	11	190	0.192982456	0.122659781	32	1256	0.561403509	0.810845707	VL	43	Pinus_strobus	1785	Pinus	Pinaceae	Pinales	namerica	gymno	49.70578889	14.88955667	ecm	87	531	131344	7	350	0.6	0.3
VL09	44	1466	12	1247	0.272727273	0.850613915	11	116	0.25	0.079126876	VL	43	Tsuga_canadensis	1812	Tsuga	Pinaceae	Pinales	namerica	gymno	49.70494278	14.88925083	ecm	87	531	131344	7	350	0.6	0.3
VL10	48	895	5	187	0.104166667	0.208938547	21	485	0.4375	0.541899441	VL	43	Pseudotsuga_menziesii	1830	Pseudotsuga	Pinaceae	Pinales	namerica	gymno	49.70502111	14.88907278	ecm	87	531	131344	7	350	0.6	0.3
VL11	62	2059	19	1505	0.306451613	0.730937348	23	259	0.370967742	0.125789218	VL	43	Abies_concolor	1910	Abies	Pinaceae	Pinales	namerica	gymno	49.70481194	14.88922444	ecm	87	531	131344	7	350	0.6	0.3
VL12	31	2667	16	2359	0.516129032	0.884514436	7	35	0.225806452	0.01312336	VL	43	Pinus_strobus	1785	Pinus	Pinaceae	Pinales	namerica	gymno	49.70601528	14.88619139	ecm	87	531	131344	7	350	0.6	0.3
VL13	36	3184	17	3030	0.472222222	0.951633166	7	68	0.194444444	0.021356784	VL	43	Thuja_occidentalis	1785	Thuja	Cupressaceae	Pinales	namerica	gymno	49.7058325	14.88656167	am	87	531	131344	7	350	0.6	0.3
VL14	54	2688	26	2458	0.481481481	0.914434524	9	66	0.166666667	0.024553571	VL	43	Tsuga_canadensis	1812	Tsuga	Pinaceae	Pinales	namerica	gymno	49.70648778	14.88586222	ecm	87	531	131344	7	350	0.6	0.3
VN02	56	1385	1	3	0.017857143	0.002166065	40	1092	0.714285714	0.788447653	VN	44	Robinia_pseudoacacia	1710	Robinia	Fabaceae	Fabales	namerica	angio	50.14091611	14.57998722	am	90	538	45752	2	250	0	1
VN03	31	661	0	0	0	0	22	441	0.709677419	0.667170953	VN	44	Robinia_pseudoacacia	1710	Robinia	Fabaceae	Fabales	namerica	angio	50.14130417	14.57931583	am	90	538	45752	2	250	0	1
VN04	46	700	1	9	0.02173913	0.012857143	30	523	0.652173913	0.747142857	VN	44	Robinia_pseudoacacia	1710	Robinia	Fabaceae	Fabales	namerica	angio	50.14130556	14.57930556	am	90	538	45752	2	250	0	1
VN05	39	541	0	0	0	0	18	365	0.461538462	0.674676525	VN	44	Aesculus_hippocastanum	1576	Aesculus	Sapindaceae	Sapindales	europe	angio	50.1331	14.57967361	am	89	567	45752	2	250	0	1
VN06	51	1114	0	0	0	0	29	806	0.568627451	0.723518851	VN	44	Aesculus_hippocastanum	1576	Aesculus	Sapindaceae	Sapindales	europe	angio	50.13313583	14.57939889	am	89	567	45752	2	250	0	1
VR01	40	1842	18	1304	0.45	0.707926167	15	146	0.375	0.079261672	VR	45	Pinus_strobus	1785	Pinus	Pinaceae	Pinales	namerica	gymno	49.37944083	14.12670167	ecm	84	575	111941	12	455	0.5625	0.1875
VR02	50	5603	6	209	0.12	0.037301446	21	334	0.42	0.059610923	VR	45	Chamaecyparis_nootkatensis	1863	Chamaecyparis	Cupressaceae	Pinales	namerica	gymno	49.37911917	14.12687667	am	84	575	111941	12	455	0.5625	0.1875
VR03	41	3423	16	3276	0.390243902	0.957055215	13	92	0.317073171	0.026877008	VR	45	Pseudotsuga_menziesii	1830	Pseudotsuga	Pinaceae	Pinales	namerica	gymno	49.37912972	14.12626139	ecm	84	575	111941	12	455	0.5625	0.1875
VR04	31	1212	5	850	0.161290323	0.701320132	13	208	0.419354839	0.171617162	VR	45	Pseudotsuga_menziesii	1830	Pseudotsuga	Pinaceae	Pinales	namerica	gymno	49.37860139	14.12627694	ecm	84	575	111941	12	455	0.5625	0.1875
VR05	49	6076	16	5019	0.326530612	0.826036866	14	743	0.285714286	0.122284398	VR	45	Tsuga_canadensis	1812	Tsuga	Pinaceae	Pinales	namerica	gymno	49.37853361	14.1264175	ecm	84	575	111941	12	455	0.5625	0.1875
VR06	33	743	10	557	0.303030303	0.749663526	16	147	0.484848485	0.197846568	VR	45	Tsuga_canadensis	1812	Tsuga	Pinaceae	Pinales	namerica	gymno	49.37844611	14.12626667	ecm	84	575	111941	12	455	0.5625	0.1875
VR07	55	1358	0	0	0	0	19	978	0.345454545	0.72017673	VR	45	Robinia_pseudoacacia	1710	Robinia	Fabaceae	Fabales	namerica	angio	49.37879222	14.12816833	am	84	575	111941	12	455	0.5625	0.1875

sample_ID	total_sps.sym	total_seq.sym	ecm_sps	ecm_seq	ecm_share_sps.sym	ecm_share.sym	patho_sps	patho_seq	patho_share_sps.sym	patho_share.sym	park	park_nr	pl_species	pl_species_age_cr	pl_genus	pl_family	pl_order	pl_origin_cont	plant_type	latitude	longitude	m_type	mat	map	area	alien.treeSR	altitude	ecm.s	angio.s
VR09	12	46	1	3	0.083333333	0.065217391	5	19	0.416666667	0.413043478	VR	45	Thuja_occidentalis	1785	Thuja	Cupressaceae	Pinales	namerica	gymno	49.37886167	14.1290025	am	84	575	111941	12	455	0.5625	0.1875
VR10	37	436	2	10	0.054054054	0.02293578	10	104	0.27027027	0.23853211	VR	45	Thuja_plicata	1844	Thuja	Cupressaceae	Pinales	namerica	gymno	49.37933444	14.13037111	am	84	575	111941	12	455	0.5625	0.1875
VR12	60	1736	19	1182	0.316666667	0.680875576	16	253	0.266666667	0.145737327	VR	45	Pinus_jeffreyi	1865	Pinus	Pinaceae	Pinales	namerica	gymno	49.37925583	14.13091917	ecm	84	575	111941	12	455	0.5625	0.1875
VR13	19	5578	7	5525	0.368421053	0.990498387	6	27	0.315789474	0.004840445	VR	45	Abies_concolor	1910	Abies	Pinaceae	Pinales	namerica	gymno	49.37996861	14.129415	ecm	84	575	111941	12	455	0.5625	0.1875
VR14	38	8049	11	7810	0.289473684	0.97030687	12	98	0.315789474	0.012175426	VR	45	Abies_concolor	1910	Abies	Pinaceae	Pinales	namerica	gymno	49.37992667	14.12934528	ecm	84	575	111941	12	455	0.5625	0.1875
VR15	54	2168	1	3	0.018518519	0.001383764	29	1923	0.537037037	0.88699262	VR	45	Ginkgo_biloba	1809	Ginkgo	Ginkgoaceae	Ginkgoales	asia	gymno	49.37993222	14.12899694	am	84	575	111941	12	455	0.5625	0.1875
VR16	81	1716	21	596	0.259259259	0.347319347	31	851	0.382716049	0.495920746	VR	45	Picea_pungens	1910	Picea	Pinaceae	Pinales	namerica	gymno	49.37973361	14.12887611	ecm	84	575	111941	12	455	0.5625	0.1875
VR17	55	802	20	426	0.363636364	0.53117207	19	238	0.345454545	0.296758105	VR	45	Sophora_japonica	1835	Sophora	Fabaceae	Fabales	asia	angio	49.37894583	14.12782972	am	84	575	111941	12	455	0.5625	0.1875
VR18	76	5387	4	3466	0.052631579	0.64340078	29	1664	0.381578947	0.308891776	VR	45	Sophora_japonica	1835	Sophora	Fabaceae	Fabales	asia	angio	49.37891472	14.12790417	am	84	575	111941	12	455	0.5625	0.1875
ZAH02	68	4209	13	3368	0.191176471	0.800190069	34	592	0.5	0.140650986	ZAH	46	Abies_concolor	1910	Abies	Pinaceae	Pinales	namerica	gymno	50.63578444	14.52558833	ecm	87	637	101855	6	275	0.454545455	0.272727273
ZAH03	72	1644	13	1040	0.180555556	0.632603406	29	241	0.402777778	0.146593674	ZAH	46	Quercus_palustris	1835	Quercus	Fagaceae	Fagales	namerica	angio	50.63642722	14.52390111	ecm	87	614	101855	6	275	0.454545455	0.272727273
ZAH04	44	1038	1	7	0.022727273	0.006743738	32	540	0.727272727	0.520231214	ZAH	46	Picea_pungens	1910	Picea	Pinaceae	Pinales	namerica	gymno	50.63514	14.523837	ecm	87	614	101855	6	275	0.454545455	0.272727273
ZAH05	44	1667	13	447	0.295454545	0.268146371	20	1009	0.454545455	0.605278944	ZAH	46	Thuja_occidentalis	1785	Thuja	Cupressaceae	Pinales	namerica	gymno	50.63518	14.525253	am	87	637	101855	6	275	0.454545455	0.272727273
ZAH06	33	1331	11	1105	0.333333333	0.830202855	15	129	0.454545455	0.096919609	ZAH	46	Thuja_occidentalis	1785	Thuja	Cupressaceae	Pinales	namerica	gymno	50.63503	14.52514	am	87	637	101855	6	275	0.454545455	0.272727273
ZAH07	62	1454	2	7	0.032258065	0.004814305	39	1074	0.629032258	0.738651994	ZAH	46	Catalpa_bignonioides	1785	Catalpa	Bignoniaceae	Lamiales	namerica	angio	50.63711583	14.52183583	am	87	614	101855	6	275	0.454545455	0.272727273
ZAH09	50	1699	12	678	0.24	0.39905827	24	912	0.48	0.536786345	ZAH	46	Liriodendron_tulipifera	1785	Liriodendron	Altingiaceae	Saxifragales	namerica	angio	50.63600944	14.52260111	am	87	614	101855	6	275	0.454545455	0.272727273
ZAH10	19	721	2	10	0.105263158	0.013869626	11	689	0.578947368	0.955617198	ZAH	46	Picea_pungens	1910	Picea	Pinaceae	Pinales	namerica	gymno	50.63514278	14.52383639	ecm	87	614	101855	6	275	0.454545455	0.272727273
ZAH11	32	384	0	0	0	0	14	209	0.4375	0.544270833	ZAH	46	Picea_pungens	1910	Picea	Pinaceae	Pinales	namerica	gymno	50.63530139	14.52413417	ecm	87	614	101855	6	275	0.454545455	0.272727273
ZAH12	33	453	0	0	0	0	21	288	0.636363636	0.635761589	ZAH	46	Thuja_occidentalis	1785	Thuja	Cupressaceae	Pinales	namerica	gymno	50.63518361	14.52525278	am	87	637	101855	6	275	0.454545455	0.272727273
ZAH13	35	550	2	51	0.057142857	0.092727273	18	313	0.514285714	0.569090909	ZAH	46	Thuja_occidentalis	1785	Thuja	Cupressaceae	Pinales	namerica	gymno	50.63502944	14.52513861	am	87	637	101855	6	275	0.454545455	0.272727273
ZB01	46	964	5	529	0.108695652	0.548755187	21	312	0.456521739	0.323651452	ZB	47	Picea_pungens	1910	Picea	Pinaceae	Pinales	namerica	gymno	49.85819194	13.76252611	ecm	78	528	38510	2	540	0.5	0.5
ZB08	44	692	0	0	0	0	27	373	0.613636364	0.539017341	ZB	47	Ilex_aquifolium	1880	Ilex	Aquifoliaceae	Aquifoliales	europe	angio	49.8578592	13.7624106	am	78	528	38510	2	540	0.5	0.5
ZH01	24	531	0	0	0	0	5	26	0.208333333	0.048964218	ZH	48	Thuja_occidentalis	1785	Thuja	Cupressaceae	Pinales	namerica	gymno	49.96780167	15.41289167	am	94	573	112085	11	220	0.111111111	0.333333333
ZH02	47	1059	2	7	0.042553191	0.006610009	26	898	0.553191489	0.847969783	ZH	48	Picea_pungens	1910	Picea	Pinaceae	Pinales	namerica	gymno	49.96784917	15.41332111	ecm	94	573	112085	11	220	0.111111111	0.333333333
ZH03	30	291	1	5	0.033333333	0.017182131	19	172	0.633333333	0.591065292	ZH	48	Picea_pungens	1910	Picea	Pinaceae	Pinales	namerica	gymno	49.96769306	15.4132625	ecm	94	573	112085	11	220	0.111111111	0.333333333
ZH04	24	302	0	0	0	0	17	270	0.708333333	0.894039735	ZH	48	Chamaecyparis_pisifera	1865	Chamaecyparis	Cupressaceae	Pinales	asia	gymno	49.96766583	15.41334111	am	94	573	112085	11	220	0.111111111	0.333333333
ZH05	32	356	0	0	0	0	23	181	0.71875	0.508426966	ZH	48	Chamaecyparis_pisifera	1865	Chamaecyparis	Cupressaceae	Pinales	asia	gymno	49.96770611	15.41373611	am	94	573	112085	11	220	0.111111111	0.333333333
ZH06	39	488	4	119	0.102564103	0.243852459	22	230	0.564102564	0.471311475	ZH	48	Thuja_occidentalis	1785	Thuja	Cupressaceae	Pinales	namerica	gymno	49.96761139	15.41418417	am	94	573	112085	11	220	0.111111111	0.333333333
ZH07	54	2027	11	1082	0.203703704	0.533793784	21	771	0.388888889	0.380365072	ZH	48	Chamaecyparis_nootkatensis	1863	Chamaecyparis	Cupressaceae	Pinales	namerica	gymno	49.96768583	15.414185	am	94	573	112085	11	220	0.111111111	0.333333333
ZH08	64	1010	5	141	0.078125	0.13960396	32	671	0.5	0.664356436	ZH	48	Juglans_cinerea	1835	Juglans	Juglandaceae	Fagales	namerica	angio	49.96763389	15.41433167	am	94	573	112085	11	220	0.111111111	0.333333333
ZH09	27	280	2	42	0.074074074	0.15	16	203	0.592592593	0.725	ZH	48	Taxodium_distichum	1835	Taxodium	Cupressaceae	Pinales	namerica	gymno	49.96778778	15.41446056	am	94	573	112085	11	220	0.111111111	0.333333333
ZH10	17	237	0	0	0	0	12	95	0.705882353	0.400843882	ZH	48	Catalpa_bignonioides	1785	Catalpa	Bignoniaceae	Lamiales	namerica	angio	49.96754639	15.41470222	am	94	573	112085	11	220	0.111111111	0.333333333
ZH13	55	1769	1	3	0.018181818	0.001695873	32	821	0.581818182	0.464104014	ZH	48	Chamaecyparis_nootkatensis	1863	Chamaecyparis	Cupressaceae	Pinales	namerica	gymno	49.96799389	15.41491	am	94	573	112085	11	220	0.111111111	0.333333333
ZH14	38	391	4	71	0.105263158	0.181585678	8	110	0.210526316	0.281329923	ZH	48	Platanus_×_acerifolia	1835	Platanus	Platanaceae	Proteales	hybrid	angio	49.96805611	15.41470194	am	94	573	112085	11	220	0.111111111	0.333333333
ZH15	75	2406	13	1502	0.173333333	0.624272652	26	517	0.346666667	0.214879468	ZH	48	Platanus_×_acerifolia	1835	Platanus	Platanaceae	Proteales	hybrid	angio	49.96837861	15.41421139	am	94	573	112085	11	220	0.111111111	0.333333333
ZH16	46	645	0	0	0	0	23	379	0.5	0.587596899	ZH	48	Magnolia_acuminata	1844	Magnolia	Magnoliaceae	Magnoliales	namerica	angio	49.96839	15.41391	am	94	573	112085	11	220	0.111111111	0.333333333
ZH17	71	2977	1	3	0.014084507	0.001007726	38	2408	0.535211268	0.808867988	ZH	48	Magnolia_acuminata	1844	Magnolia	Magnoliaceae	Magnoliales	namerica	angio	49.96838611	15.41391167	am	94	573	112085	11	220	0.111111111	0.333333333
ZH18	35	850	2	469	0.057142857	0.551764706	17	212	0.485714286	0.249411765	ZH	48	Metasequoia_glyptostroboides	1949	Metasequoia	Cupressaceae	Pinales	asia	gymno	49.96812139	15.41379444	am	94	573	112085	11	220	0.111111111	0.333333333
ZH19	66	1169	2	11	0.03030303	0.009409752	40	944	0.606060606	0.807527802	ZH	48	Metasequoia_glyptostroboides	1949	Metasequoia	Cupressaceae	Pinales	asia	gymno	49.96848833	15.41341778	am	94	573	112085	11	220	0.111111111	0.333333333
ZH20	44	933	0	0	0	0	30	583	0.681818182	0.624866024	ZH	48	Ginkgo_biloba	1809	Ginkgo	Ginkgoaceae	Ginkgoales	asia	gymno	49.96858861	15.41360083	am	94	573	112085	11	220	0.111111111	0.333333333

Table S3

sample_id	park	group	lattude	longitude	cores	mat	map	alttude	alien.treeSR
AZ	AZ	alien	49.46153	13.15816	5	78.71429	811.8571	515	6
BH	BH	alien	49.71004	16.97666	6	90	607	320	13
BLUD	BLUD	alien	49.93917	16.92964	5	87	682	300	7
CK	CK	alien	48.81094	14.30832	1	85	594	550	3
CR	CR	alien	49.90001	15.93895	10	89	603.1429	284	15
DB	DB	alien	49.77902	14.18013	2	86	463	365	5
DO	DO	alien	50.45511	14.15844	5	94.6	485	150	5
HL	HL	alien	49.05163	14.4407	11	88.76923	637.9231	440	17
HN	HN	alien	50.22426	15.6675	4	87	706.4444	295	5
HT	HT	alien	49.53184	12.94057	6	86.66667	724.1667	380	4
CHO	CHO	alien	50.51294	13.44393	3	81	620	480	2
JE	JE	alien	49.74387	14.7867	4	82	613	410	8
KA	KA	alien	49.98055	15.34537	5	93.27273	582.7273	225	6
KD	KD	alien	50.25158	13.3699	4	88	550	320	2
KK	KK	alien	50.15854	15.45133	5	91	589.1429	240	6
KNO	KNO	alien	50.38421	13.17314	4	85.375	611	300	7
KO	KO	alien	49.77741	14.65782	7	86.76923	593.5385	375	10
KP	KP	alien	50.33061	15.27236	8	89.90909	659.2727	225	16
KROM	KROM	alien	49.30431	17.39279	4	98	561.3333	190	15
KYN	KYN	alien	50.00414	12.60544	11	71	720	600	7
KZ	KZ	alien	49.66971	13.52987	7	85.72727	570.4545	390	11
LDN	LDN	alien	50.40757	14.44493	4	93.57143	550.7143	170	4
LI	LI	alien	48.80227	16.80793	1	103	482.3846	170	13
LIB	LIB	alien	50.40475	14.04746	11	94	481	165	5
LL	LL	alien	50.20311	14.83534	5	93.4	573.6	210	3
LO	LO	alien	50.28746	15.02536	2	90.27273	614.2727	240	10
ME	ME	alien	50.19511	14.52212	4	93	489	200	9
NH	NH	alien	48.79106	14.78231	2	83.33333	699	510	5
NOSV	NOSV	alien	49.03369	17.80022	4	94	601.4286	310	6
OR	OR	alien	49.51163	14.17127	8	88	493.4706	375	11
PASK	PASK	alien	49.73123	18.29471	3	91	794	250	5
PB	PB	alien	50.21218	14.43761	5	89	534	270	5
PL	PL	alien	50.56012	14.20112	1	90	520	225	9
PR	PR	alien	49.9937	14.55737	11	87.72414	553.8276	325	18
SL	SL	alien	49.918	15.80878	8	89.36364	548	287	19
SYCH	SYCH	alien	50.62613	15.0911	8	80.66667	758.6667	375	11
TE	TE	alien	49.18581	15.44845	7	81	548.75	511	6
VE	VE	alien	50.27627	14.32986	8	94	456	180	5
VEHE	VEHE	alien	49.9754	17.73668	3	84	625	335	9
VELO	VELO	alien	50.02282	17.03127	2	80	657	380	6
VES	VES	alien	49.53067	17.50601	6	91	619	275	8
VJ	VJ	alien	49.66966	14.57997	10	84	524	425	15
VL	VL	alien	49.70587	14.89002	6	86.7	533.4	350	7
VN	VN	alien	50.13795	14.57954	5	89.6	549.6	250	2

VR	VR	alien	49.3792	14.12804	9	84	575	455	12
ZAH	ZAH	alien	50.63558	14.52423	5	87	624.4545	275	6
ZB	ZB	alien	49.85803	13.76247	1	78	528	540	2
ZH	ZH	alien	49.96797	15.41394	2	94	573	220	11
BHF	BH	forest	49.7123	17.02388	20	89	629	320	13
BHP	BH	park	49.70551	16.98616	20	89	629	320	13
HLF	HL	forest	49.05169	14.50788	20	88	641	440	17
HLP	HL	park	49.05313	14.43778	20	88	641	440	17
HTF	HT	forest	49.54517	12.98832	20	85	761	380	4
HTP	HT	park	49.53365	12.93887	20	85	761	380	4
JEF	JE	forest	49.74848	14.79794	20	82	613	410	8
JEP	JE	park	49.743	14.78805	20	82	613	410	8
KAF	KA	forest	49.96906	15.32218	20	94	587	225	6
KAP	KA	park	49.98342	15.34121	20	94	587	225	6
KDF	KD	forest	50.20771	13.37996	20	85	570	320	2
KDP	KD	park	50.2472	13.3652	20	85	570	320	2
KKF	KK	forest	50.15385	15.43864	20	91	614	240	6
KKP	KK	park	50.15995	15.44885	20	91	614	240	6
KNOF	KNO	forest	50.37642	13.13903	20	86	613	300	7
KNOP	KNO	park	50.38448	13.17895	20	86	613	300	7
KOF	KO	forest	49.77885	14.67295	20	86	602	375	10
KOP	KO	park	49.77814	14.66	20	86	602	375	10
KPF	KP	forest	50.31914	15.25112	20	89	672	225	16
KPP	KP	park	50.33185	15.27944	20	89	672	225	16
KROMF	KROM	forest	49.21243	17.42177	20	98	560	190	15
KROMP	KROM	park	49.30705	17.38873	20	98	560	190	15
KYNF	KYN	forest	49.98333	24.151	20	71	757	600	7
KYNP	KYN	park	50.00689	12.60918	20	71	757	600	7
KZF	KZ	forest	49.69368	13.45881	20	84	608	390	11
KZP	KZ	park	49.66989	13.53447	20	84	608	390	11
LDNF	LDN	forest	48.81451	16.83774	20	103	483	170	13
LDNP	LDN	park	48.80785	16.81387	20	103	483	170	13
LIF	LI	forest	50.40574	14.12116	20	94	478	165	5
LIP	LI	park	50.4037	14.05044	20	94	478	165	5
LOF	LO	forest	50.29072	15.02011	20	91	603	240	10
LOP	LO	park	50.28624	15.02512	20	91	603	240	10
NHF	NH	forest	50.20057	14.5284	20	84	717	510	5
NHP	NH	park	48.93982	14.78313	20	84	717	510	5
NOSVF	NOSV	forest	48.79258	14.78377	20	94	604	310	6
NOSVP	NOSV	park	49.02627	17.79483	20	94	604	310	6
ORF	OR	forest	49.03364	17.80165	20	87	501	375	11
ORP	OR	park	49.50024	14.17425	20	87	501	375	11
PASKF	PASK	forest	49.51174	14.15912	20	91	794	250	5
PASKP	PASK	park	49.7236	18.26511	20	91	794	250	5
PBF	PB	forest	49.73038	18.2963	20	89	534	270	5
PBP	PB	park	50.21714	14.43797	20	89	534	270	5

PLF	PL	forest	50.21259	14.43933	20	90	520	225	9
PLP	PL	park	50.56828	14.17693	20	90	520	225	9
PRF	PR	forest	50.55901	14.20306	20	87	559	287	18
PRP	PR	park	50.01789	14.53584	20	87	559	287	18
VEF	VE	forest	49.99113	14.53971	20	94	457	335	5
VEP	VE	park	50.34986	14.33048	20	94	457	335	5
VEHEF	VEHE	forest	49.96813	17.78878	20	84	625	380	9
VEHEP	VEHE	park	49.97497	17.73993	20	84	625	380	9
VELOF	VELO	forest	49.97599	16.9944	20	80	657	180	6
VELOP	VELO	park	50.02232	17.03278	20	80	657	180	6
VESF	VES	forest	49.53292	17.50355	20	91	619	275	8
VESP	VES	park	49.53041	17.50652	20	91	619	275	8
VJF	VJ	forest	49.70035	14.59905	20	84	524	425	15
VJP	VJ	park	49.66841	14.5822	20	84	524	425	15
VLF	VL	forest	49.71669	14.82945	20	87	531	350	7
VLP	VL	park	49.70518	14.88627	20	87	531	350	7
VNF	VN	forest	50.12929	14.57683	20	90	535	250	2
VNP	VN	park	50.13615	14.58438	20	90	535	250	2
ZAHF	ZAH	forest	50.61905	14.54055	20	87	614	275	6
ZAHP	ZAH	park	50.63724	14.52216	20	87	614	275	6
ZBF	ZB	forest	49.83139	13.80455	20	78	528	540	2
ZBP	ZB	park	49.85797	13.76248	20	78	528	540	2
ZHF	ZH	forest	49.97533	15.35662	20	94	577	220	11
ZHP	ZH	park	49.96635	15.41627	20	94	577	220	11

Supplementary Table S3: Native assemblage metadata for park and forest plots (n = 76 composite samples): assemblage type (alien, park-native or forest-native), canopy cover (%), environmental covariates, GPS.

Table S4

Pathogen relatve richness (sps)

```
patho_share_sps.sym ~ plant_type + m_type + pl_origin_cont +

    scale(mat) + scale(map) + scale(area) +
scale(alien.treeSR) +
    scale(altitude) + scale(ecm.s) + scale(angio.s) + scale(pl_species_age_cr) +

    (1 | park_nr)

                         npar    AIC
LRT   Pr(Chi)
<none>                        3532.2

plant_type                  1 3535.0  4.854
0.0275785 *
m_type                      1 3593.7 63.515 1.592e-
15 ***
pl_origin_cont              3 3541.5 15.327
0.0015572 **
scale(mat)                  1 3557.6 27.384 1.668e-
07 ***
scale(map)                  1 3532.6  2.370
0.1236620
scale(area)                 1 3533.1  2.877
0.0898566 .
scale(alien.treeSR)         1 3532.1  1.895
0.1685852
scale(altitude)             1 3537.4  7.256
0.0070670 **
scale(ecm.s)                1 3530.4  0.200
0.6549256
scale(angio.s)              1 3531.8  1.596
0.2064106
scale(pl_species_age_cr)    1 3543.4 13.235 0.0002748 ***

---

Signif. codes:  0 ‘***’ 0.001 ‘**’ 0.01 ‘*’ 0.05 ‘.’ 0.1 ‘ ’ 1
```

Pathogen relatve abundance (seq)

```
patho_share.sym ~ plant_type + m_type + pl_origin_cont + scale(mat) +

    scale(map) + scale(area) + scale(alien.treeSR) + scale(altitude) +

    scale(ecm.s) + scale(angio.s) + scale(pl_species_age_cr) +

    (1 | park_nr) + (1 | sample_ID)

                         npar    AIC
LRT   Pr(Chi)
<none>                        7206.0

plant_type                  1 7204.6  0.618
0.431739
m_type                      1 7248.8 44.789 2.194e-
11 ***
pl_origin_cont              3 7204.5  4.533
0.209343
scale(mat)                  1 7208.1  4.103
0.042819 *
scale(map)                  1 7205.9  1.926
0.165165
scale(area)                 1 7204.5  0.487
0.485463
scale(alien.treeSR)         1 7206.4  2.478
0.115453
scale(altitude)             1 7204.0  0.029
0.865786
scale(ecm.s)                1 7205.9  1.897
0.168440
scale(angio.s)              1 7204.8  0.886
0.346683
scale(pl_species_age_cr)    1 7214.5 10.575  0.001146 **

---

Signif. codes:  0 ‘***’ 0.001 ‘**’ 0.01 ‘*’ 0.05 ‘.’ 0.1 ‘ ’ 1
```

Supplementary Table S4: Robustness of pathogen GLMMs to AM fungal under-detection: results after excluding AM fungal taxa from denominators (Hypothesis 2). For each model, we report the formula, weights/denominator definition, likelihood-ratio χ^2, df, P, and AIC. Results mirror the main models in Table 2.

Table S5

Contngency table: plant_type × pl_origin_cont

plant_type	asia	europe	hybrid	namerica
angio	46	26	38	128
gymno	41	6	0	235

Contngency table: m_type × pl_origin_cont

m_type	asia	europe	hybrid	namerica
am	79	6	38	174
ecm	8	26	0	189

3-way table: plant_type × m_type × pl_origin_cont

plant_type	m_type	asia	europe	hybrid	namerica
angio	am	46	6	38	112
angio	ecm	0	20	0	16
gymno	am	33	0	0	62
gymno	ecm	8	6	0	173

metric	value
Chi-squared test (plant_type × origin)	p < 2.2e-16
Cramer's V (plant_type × origin)	0.3902
Note	Sparse/zero cells exist (e.g., hybrid occurs only in AM hosts; several plant_type × m_type × origin combinatons are absent).

Analysis	Response term	Dataset	Df	R2	Chi2/F	p-value	
H1 PERMANOVA (adonis2, by="margin")	plant_type	All origins (incl. hybrid)	1	0.005466	F=3.2262	0.001	
H1 PERMANOVA (adonis2, by="margin")	pl_origin_cont:m_type	All origins (incl. hybrid)	2	0.004783	F=1.4117	0.005	
H1 PERMANOVA (adonis2, by="margin")	plant_type	No-hybrid	1	0.005975	F=3.2650	0.001	
H1 PERMANOVA (adonis2, by="margin")	pl_origin_cont:m_type	No-hybrid	2	0.005206	F=1.4222	0.004	
GLMM Chi² and P are likelihood-rato tests from single -term deleton (drop1)							
H2 GLMM (binomial; ECM hosts only)	pl_origin_cont	All origins (incl. hybrid)	2	NA	Chi2=19.0031	7.47E-05	Efect unchanged when excluding hybrid; fxed efects: Europe=0.0167, N. America=0.4915 (vs Asia ref.)
H2 GLMM (binomial; ECM hosts only)	pl_origin_cont	No-hybrid	2	NA	Chi2=19.0031	7.47E-05	Same as baseline
H2 GLMM (binomial; all alien trees)	pl_origin_cont	All origins (incl. hybrid)	3	NA	Chi2=14.848	0.001951	Signal driven by hybrid category (see sensitvity)
H2 GLMM (binomial; all alien trees)	pl_origin_cont	No-hybrid	2	NA	Chi2=4.041	0.1326	Contnental origin efect not robust without hybrid
Interacton test: LRT comparing models with vs without m_type:pl_origin_cont.							
H2 GLMM interacton test	m0 vs m1 (add m_type:pl_origin_cont)	All origins (incl. hybrid)	2	NA	Chi2=2.943	0.2296	Model matrix rank defcient due to sparse combinatons
H2 GLMM interacton test	m0_nh vs m1_nh (add m_type:pl_origin_cont)	No-hybrid	2	NA	Chi2=1.6738	0.433	Interacton not supported

Supplementary Table S5: Contingency tables (χ^2, Cramér’s V) and no-hybrid sensitivity analyses addressing partial dependence among biogeographical origin, mycorrhizal type and plant group.

Table S6

=== FULL PERMANOVA TABLE (final model) ===

Term	df	pseudoF	R2	P
PCNM1	1	6.051812	0.010267670	0.103
PCNM2	1	2.661173	0.004515018	0.009
PCNM3	1	2.418555	0.004103386	0.415
PCNM4	1	2.417241	0.004101157	0.034
PCNM5	1	1.315970	0.002232710	0.731
PCNM6	1	1.447126	0.002455233	0.006
PCNM7	1	1.884560	0.003197396	0.004
PCNM9	1	2.037612	0.003457068	0.191
PCNM10	1	1.652467	0.002803621	0.027
PCNM11	1	2.058188	0.003491979	0.022
PCNM12	1	2.307513	0.003914989	0.002
PCNM13	1	3.306069	0.005609168	0.018
PCNM14	1	1.587676	0.002693694	0.491
PCNM15	1	2.199764	0.003732179	0.224
PCNM16	1	1.844334	0.003129147	0.749
PCNM17	1	1.747792	0.002965352	0.001
PCNM18	1	1.769153	0.003001593	0.040
PCNM19	1	1.934484	0.003282098	0.010
PCNM20	1	1.443146	0.002448481	0.672
PCNM21	1	2.075987	0.003522176	0.043
PCNM22	1	1.589630	0.002697010	0.012
PCNM23	1	1.808425	0.003068223	0.048
PCNM35	1	1.793492	0.003042888	0.092
PCNM37	1	1.864917	0.003164068	0.001
PCNM38	1	1.718205	0.002915153	0.347
PCNM39	1	2.200192	0.003732906	0.001
PCNM42	1	1.760886	0.002987568	0.005
PCNM43	1	1.611220	0.002733639	0.010
PCNM45	1	1.030905	0.001749061	0.300
PCNM46	1	1.551904	0.002633003	0.010
PCNM48	1	1.831497	0.003107368	0.001
pl_species_age_cr	1	1.858908	0.003153873	0.002
plant_type	1	3.100804	0.005260909	0.001
mat	1	2.636189	0.004472629	0.011
map	1	1.705252	0.002893178	0.216
area	1	2.781418	0.004719029	0.199
ecm.s	1	1.705191	0.002893074	1.000
angio.s	1	1.821656	0.003090671	0.368
alien.treeSR	1	1.950974	0.003310076	0.330

BETADISPER

```
--- betadisper: Origin ---
grp
namerica     asia   hybrid   europe
     363       87
38       32

Group mean distance ± SE to centroid:
    Group  MeanDist
SE
     asia 0.5502569
0.007997926
   europe 0.5927874 0.008270479
   hybrid 0.5348285 0.011240563
 namerica 0.5859341 0.003380442

Permutation test (blocked by park):

Permutation test for homogeneity of
multivariate dispersions
Blocks:  env$park_nr
Permutation: free
Number of permutations: 999

Response: Distances
           Df  Sum Sq  Mean Sq      F N.Perm
Pr(>F)
Groups      3 0.16669 0.055563 12.881    999
0.001 ***
Residuals 516 2.22573 0.004313
---
Signif. codes:  0 '***' 0.001 '**' 0.01 '*'
0.05 '.' 0.1 ' ' 1

--- betadisper: Mycorrhizal type --
-
grp
 am ecm
 297
 223

Group mean distance ± SE to centroid:
 Group  MeanDist          SE
    am 0.5564807 0.004045726
   ecm 0.5984416 0.003921904

Permutation test (blocked by park):

Permutation test for homogeneity of
multivariate dispersions
Blocks:  env$park_nr
```

```
 pl_origin_cont:m_type   2 1.425202 0.004836074
0.004
             Residual 474       NA 0.804201325
NA
                Total 519       NA
1.000000000    NA
```

=== PCoA variance explained (%) ===

```
Axis 1: 7.46%
Axis 2: 3.70%
Axis 3: 3.47%
```

```
Permutation test for adonis under reduced model
Marginal effects of terms
Blocks:  env$park_nr
Permutation: free
Number of permutations: 999

adonis2(formula = frm, data = env_combined,
permutations = perm, by = "margin")
                  Df SumOfSqs        R2      F
Pr(>F) Variable
pl_origin_cont     3  2.35348 0.0132207 2.4992  0.001
8
pl_species_age_cr  1  0.98559 0.0055366 3.1247
0.001       9
m_type             1  3.00347 0.0168720 9.6489  0.001
5
plant_type         1  2.23204 0.0125385 7.1343
0.001      10
mat                1  1.12102 0.0062973 3.5572
0.218       7
map                1  0.66153 0.0037161 2.0929
0.109       6
area               1  0.86614 0.0048656 2.7439
0.535       3
ecm.s              1  1.12325 0.0063099 3.5643  0.309
4
angio.s            1  0.67024 0.0037651 2.1206
0.530       2
alien.treeSR       1  0.64366 0.0036158 2.0361
0.199       1

        Variable         Comparison        F
R2 p_value
1 pl_origin_cont     asia vs europe 2.942618
0.022165731   0.001
2 pl_origin_cont   asia vs namerica 2.924465
0.005975744   0.001
3 pl_origin_cont   europe vs hybrid 2.075421 0.024973161   0.001
4 pl_origin_cont europe vs namerica 2.254563 0.005259035   0.001
5 pl_origin_cont hybrid vs namerica 2.220730 0.005067228   0.001
6 pl_origin_cont     asia vs hybrid 1.413468 0.010161972   0.005
```

myco type

```
  Variable Comparison        F         R2
p_value
1   m_type  am vs ecm 9.648908 0.01687203   0.001
```

plant type

```
    Variable     Comparison        F         R2
p_value
1 plant_type angio vs gymno 7.134303 0.01253849   0.001
```

```
Permutation: free
Number of permutations: 999

Response: Distances
           Df  Sum Sq  Mean Sq      F N.Perm
Pr(>F)
Groups      1 0.22426 0.224258 52.793    999
0.001 ***
Residuals 518 2.20040 0.004248
---
Signif. codes:  0 ‘***’ 0.001 ‘**’ 0.01 ‘*’
0.05 ‘.’ 0.1 ‘ ’ 1
```

--- betadisper: Plant group ---

```
grp
gymno angio
  282
238
Group mean distance ± SE to centroid:
 Group  MeanDist          SE
 angio 0.5629146 0.004592943
 gymno 0.5879672 0.003869710

Permutation test (blocked by park):

Permutation test for homogeneity of
multivariate dispersions
Blocks:  env$park_nr
Permutation: free
Number of permutations: 999

Response: Distances
           Df  Sum Sq  Mean Sq      F N.Perm
Pr(>F)
Groups      1 0.08101 0.081008 17.657    999
0.001 ***
Residuals 518 2.37651 0.004588
---
Signif. codes:  0 ‘***’ 0.001 ‘**’ 0.01 ‘*’
0.05 ‘.’ 0.1 ‘ ’ 1
```

Supplementary Table S6: Full marginal PERMANOVA results for drivers of alien-tree fungal community composition (biogeographical origin, mycorrhizal type, plant group, residence time, environmental covariates; spatial PCNMs; blocked by park; i.e., Hypothesis 1).

Table S7

Phylum	No. of SHs	%
Ascomycota	2571	45.2
Basidiomycota	1132	19.9
**Fungi incertae sedis**	755	13.3
Rozellomycota	459	8.1
Chytridiomycota	441	7.8
Glomeromycota	139	2.4
Mortierellomycota	76	1.3
Mucoromycota	39	0.7
Olpidiomycota	18	0.3
Kickxellomycota	14	0.2

Supplementary Table S7: Taxonomic composition of unassigned SHs. Counts and percentages of SHs lacking a confident trophic-guild assignment under conservative criteria, summarized by phylum (and top classes/families/genera). The unassigned set is dominated by Ascomycota and Basidiomycota, with substantial representation of lineages marked incertae sedis at lower ranks, consistent with current gaps in trait coverage and taxonomy. These taxa were excluded from guild-based proportions to avoid misclassification.

Table S8

Full model

```
Permutation test for adonis under reduced model
Marginal effects of terms
Blocks:  env$ParkID
Permutation: free
Number of permutations: 999

adonis2(formula = D ~ cores_sc + MAT_sc + MAP_sc + Alt_sc + PCNM2 + group, data = env, permutations = perm, by = "margin")
          Df SumOfSqs      R2      F Pr(>F)
MAP_sc     1    0.486 0.01286 1.7595  0.001 ***
group      2    3.335 0.08833 6.0410  0.001 ***
MAT_sc     1    0.396 0.01048 1.4338  0.002 **
PCNM2      1    0.497 0.01317 1.8010  0.002 **
cores_sc   1    0.480 0.01272 1.7399  0.023 *
Alt_sc     1    0.366 0.00970 1.3272  0.088 .
Residual 106   29.257 0.77498
Total    113   37.752 1.00000
---
Signif. codes:  0 ‘***’ 0.001 ‘**’ 0.01 ‘*’ 0.05 ‘.’ 0.1 ‘ ’ 1
```

AdjR2 = 0.1680

Pair-wise PERMANOVA (Bonferroni)

```
 group1 group2         F p_raw p_adj
  alien   park 11.999832 1e-04 3e-04
  alien forest 15.817709 1e-04 3e-04
   park forest  2.112766 1e-04 3e-04
```

Supplementary Table S8: Blocked PERMANOVA comparing fungal community composition across park-alien, park-native, and forest-native assemblages (including Bonferroni-adjusted pairwise tests; i.e., Hypothesis 3).

Table S9

ECM relatve richness

Model: glm, binomial

cbind(ecm_R, ecm_RF) ~ group + scale(mat) + scale(map) + scale(altitude) + scale(ecm.s) + (1 | ParkID) + (1 | sample_ID)

	npar	AIC	LRT	Pr(Chi)	
<none>		797.50			
group	2	826.50	33.000	6.825e-08	***
scale(mat)	1	795.57	0.068	0.7938	
scale(map)	1	798.10	2.601	0.1068	
scale(altitude)	1	797.89	2.396	0.1217	
scale(ecm.s)	1	813.09	17.595	2.733e-05	***

contrast	odds.ratio	SE	df	null	z.ratio	p.value	
Alien vs Others	0.384	0.0545	Inf	1	-6.749	<.0001	*

contrast	odds.ratio	SE	df	null	z.ratio	p.value	
alien / park	0.393	0.0652	Inf	1	-5.625	<.0001	*
alien / forest	0.375	0.0618	Inf	1	-5.948	<.0001	*
park / forest	0.954	0.1624	Inf	1	-0.278	0.9582	

ECM relatve abundance

Model: glm, binomial

cbind(ecm_A, ecm_AF) ~ group + scale(mat) + scale(map) + scale(altitude) + scale(ecm.s) + (1 | ParkID) + (1 | sample_ID)

	npar	AIC	LRT	Pr(Chi)	
<none>		1612.9			
group	2	1609.2	0.3049	0.85861	
scale(mat)	1	1611.1	0.2346	0.62811	
scale(map)	1	1616.0	5.1584	0.02313	*
scale(altitude)	1	1611.3	0.3696	0.54322	
scale(ecm.s)	1	1627.7	16.7605	4.241e-05	***

contrast	odds.ratio	SE	df	null	z.ratio	p.value
Alien vs Others	1.18	0.407	Inf	1	0.479	0.6316

contrast	odds.ratio	SE	df	null	z.ratio	p.value
alien / park	1.251	0.507	Inf	1	0.553	0.8449
alien / forest	1.112	0.441	Inf	1	0.268	0.9611
park / forest	0.889	0.364	Inf	1	-0.288	0.9554

PATHOGENS relatve richness

Model: glm, binomial

cbind(patho_R, patho_RF) ~ group + scale(mat) + scale(map) + scale(altitude) + scale(ecm.s) + (1 | ParkID) + (1 | sample_ID)

	npar	AIC	LRT	Pr(Chi)	
<none>		757.08			
group	2	825.62	72.537	< 2.2e-16	***
scale(mat)	1	757.55	2.470	0.1160	
scale(map)	1	755.11	0.023	0.8789	
scale(altitude)	1	755.21	0.129	0.7192	
scale(ecm.s)	1	773.41	18.328	1.859e-05	***

contrast	odds.ratio	SE	df	null	z.ratio	p.value	
Alien vs Others	2.47	0.21	Inf	1	10.611	<.0001	*

contrast	odds.ratio	SE	df	null	z.ratio	p.value	
alien / park	2.10	0.211	Inf	1	7.396	<.0001	*
alien / forest	2.90	0.301	Inf	1	10.218	<.0001	*
park / forest	1.38	0.156	Inf	1	2.825	0.0131	*

PATHOGENS relatve abundance

Model: glm, binomial

cbind(patho_A, patho_AF) ~ group + scale(mat) + scale(map) + scale(altitude) + scale(ecm.s) + (1 | ParkID) + (1 | sample_ID)

	npar	AIC	LRT	Pr(Chi)	
<none>		1515.5			
group	2	1540.0	28.5343	6.366e-07	***
scale(mat)	1	1515.1	1.6309	0.2016	
scale(map)	1	1514.0	0.5372	0.4636	
scale(altitude)	1	1513.5	0.0465	0.8294	
scale(ecm.s)	1	1533.0	19.4759	1.019e-05	***

contrast	odds.ratio	SE	df	null	z.ratio	p.value	
Alien vs Others	3.25	0.76	Inf	1	5.050	<.0001	*

contrast	odds.ratio	SE	df	null	z.ratio	p.value	
alien / park	2.18	0.599	Inf	1	2.851	0.0121	*
alien / forest	4.84	1.301	Inf	1	5.877	<.0001	*
park / forest	2.22	0.612	Inf	1	2.887	0.0109	*

Supplementary Table S9: Binomial GLMM outputs for ECM and pathogen relative richness and abundance across tree assemblages (alien vs park-native vs forest-native) with environmental covariates and random effects (Hypothesis 3).
